# Divalent Intermediates in Lanthanide-Based Photocatalysts: Spectroscopic Characterization and Reactivity

**DOI:** 10.1021/acs.inorgchem.4c03926

**Published:** 2024-12-23

**Authors:** Monika Tomar, Anders Thapper, Andreas Orthaber, K. Eszter Borbas

**Affiliations:** Department of Chemistry, Ångström Laboratory, Uppsala University, 75120 Uppsala, Sweden

## Abstract

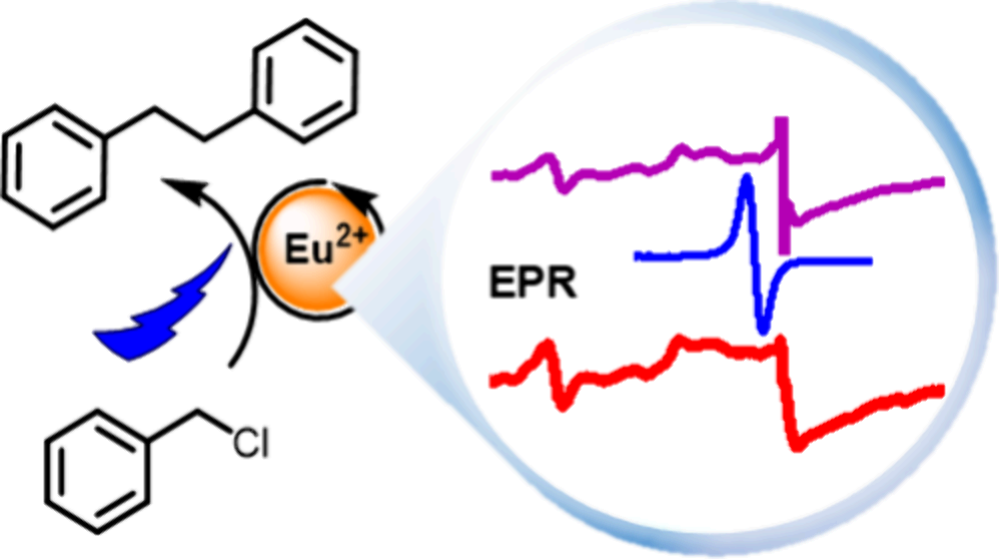

The reduction of stable trivalent lanthanide species (Ln(III)) by the excited states of organic chromophores is the basis of photocatalytic divalent lanthanide-mediated reduction reactions. While indirect evidence of the photochemical formation of the reactive Ln(II) species is abundant, direct spectroscopic evidence of their presence is scarce. Here, nine chromophores with absorptions covering the near UV and visible ranges were systematically investigated in the presence of Ln(III) ions to evaluate their ability to reduce Eu(III) upon excitation with visible light to the catalytically active Eu(II) species. Irradiated mixtures of Eu(III) and the chromophores were characterized using UV–vis absorption and emission and EPR spectroscopy. Several of the chromophore-Eu(III) combinations were competent photocatalysts in the presence of N,N-diisopropylethylamine or Zn terminal reductants. These results demonstrate that a variety of visible-absorbing chromophores can efficiently generate reactive Eu(II) from Eu(III) to catalyze Ln(II)-mediated reduction reactions.

## Abstract 2

Photoinduced electron transfer from a variety of organic chromophores to Eu(III) yielded Eu(II). Mixtures of Eu(OTf)_3_ and the chromophores were irradiated with blue light and were then characterized using UV−vis absorption and emission spectroscopy. EPR spectroscopy showed the presence of Eu(II) in the irradiated mixtures. The reactivities of the Eu(III)-chromophore combinations were explored in catalytic settings, and several new active photocatalysts were identified.

## Introduction

The most stable oxidation state of the lanthanides (Ln) is +3. With the notable exceptions of Ce(IV) oxidants^1,2^ and Sm(II) reductants,^3^ Ln(II) and Ln(IV) compounds have only recently gained attention. Small-molecule complexes of all nonradioactive Ln(II) ions are now known,^4−11^ as are several Ce(IV),^1,12−15^ and a handful of Tb(IV)^16−19^ and Pr(IV)^20,21^ ones. These exciting studies have opened up new avenues of application and highlighted the role of redox processes in altering the physical properties of Ln(III) species.^9,22,23^

Ln(II) complexes can be synthesized from Ln(III) precursors. Eu(II) is the most stable Ln(II) because of its half-filled 4f^7^ electronic configuration,^24^ and can be obtained by the reduction of Eu(III) with Zn.^25^ Stronger reductants, e.g., KC_8_ is needed to access Gd(II) and Tb(II), the least stable Ln(II) ions.^4^ The harshness of these conditions limits the scope of useful Ln(III) precursors and places constraints on applications relying on Ln(II)/Ln(III) interconversions. Photochemical reductions by excited-state chromophores allow for the use of milder conditions than those relying on ground-state chemical reductants.^26,27^ Such an approach has environmental benefits as some of the energy required to access the product comes from light.

**Figure 1:**
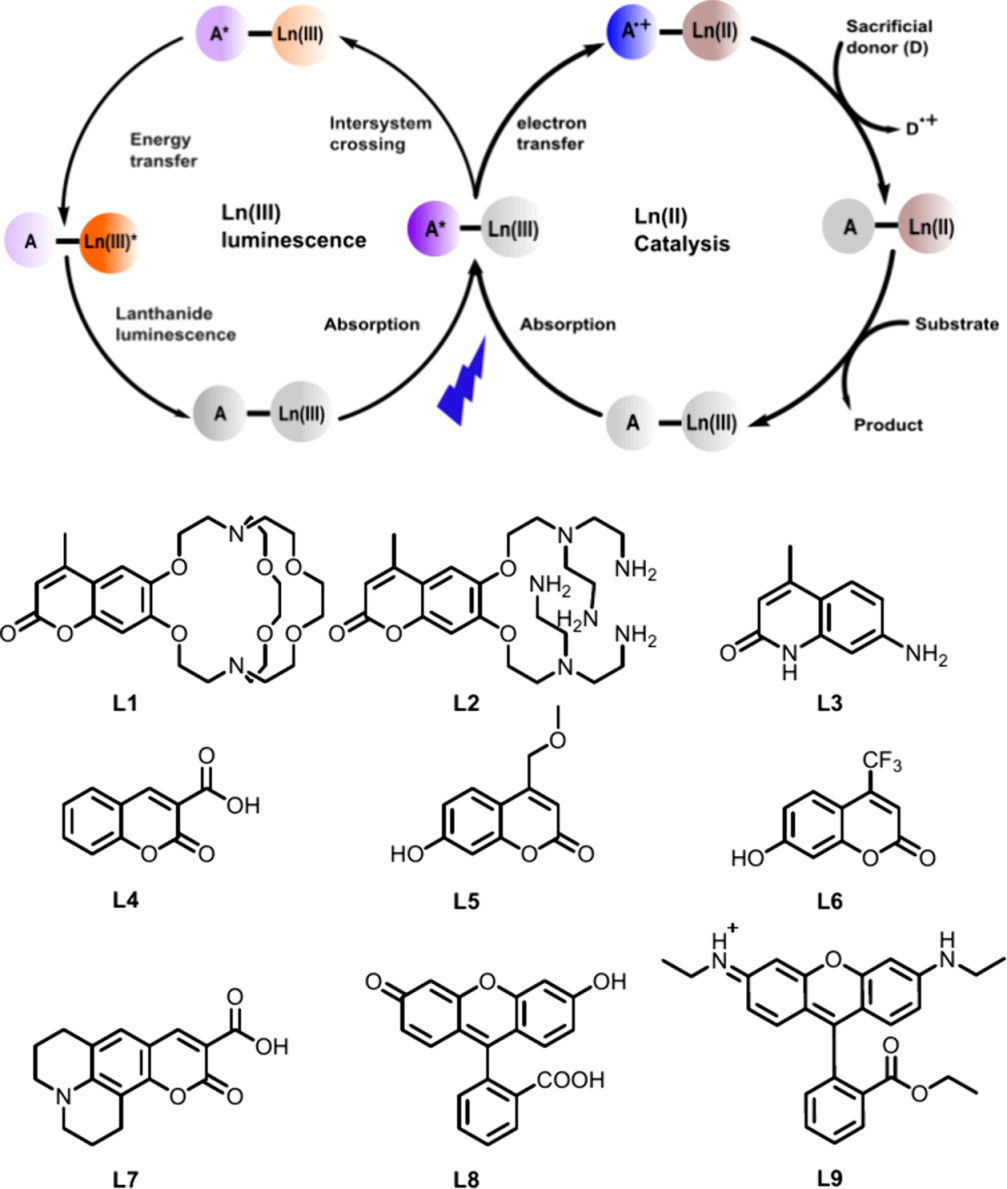
Proposed catalytic cycle for Ln(II)-mediated reductions as a parallel process to Ln(III) luminescence sensitization (top), and **L1**–**L9** chromophores investigated here for Ln(II)-generation (bottom).

Photochemical Ln(II) generation is a possibility for several Ln(III)-chromophore (**LnL**) combinations.^27−32^ Most Ln(III) ions are luminescent; emissions are due to 4f–4f transitions, and can be sensitized via energy transfer from a light-harvesting antenna.^33−36^ Competing electron transfer to the more reducible Ln(III) centers (Ln = Eu, Sm, Yb) is also possible,^28−30,37^ in some cases resulting in dramatic Ln(III) emission quenching (Figure 1).^38^ The transiently formed Ln(II) species has been harnessed in organic synthesis.^26,27,39,40^ Substrates ranging from benzyl and aryl halides to P=O, C=X, and X=X bonds can be reduced with catalytic amounts of **LnL** upon visible light irradiation. However, while an abundance of indirect evidence supports Ln(II) formation via reduction by chromophore excited states, direct spectroscopic evidence for the presence of Ln(II) in such systems is scarce.^27,39^

Antennae capable of donating electrons in their excited states and easily reducible Ln(III) that can accept electrons are necessary to initiate Ln(II) catalysis. Photocatalysts **LnL1**, **LnL2** and **LnL3** (Figure 1) could perform many of the reactions previously mediated by stoichiometric Sm(II)-reagents with excellent yield and selectivity, however, their absorptions are mostly in the near UV.^27^ Chromophores that absorb visible light well have excited state reduction potentials that are still sufficiently negative to reduce a variety of Ln(III) ions (e.g., Ln = Eu, Yb, Sm).^30^ The use of lower-energy light would enable the reduction of sensitive substrates and, as a long-term goal, the use of solar energy to drive the reactions.

Here, we investigated photoinduced electron transfer (PeT) to Ln(III) (Ln = Eu, Sm) from the excited chromophores **L4**–**L9** (Figure 1). **L** absorb strongly in the 300–550 nm range and have versatile electronic, steric, and coordination properties. Mixtures of **L** and Ln(OTf)_3_ were studied using IR, NMR, absorption and emission spectroscopy, and cyclic voltammetry. The irradiated mixtures were characterized using UV–vis absorption and emission spectroscopy, and the EPR spectroscopic fingerprints of the photochemically generated Eu(II) ions were also obtained. Finally, the **L**-Eu(III) combinations (**EuL**) were evaluated as photocatalysts. Taken together, these results show that a broad range of visible-absorbing chromophores yield reactive Ln(II) intermediates and are thus suitable components of Ln-based photocatalysts for divalent lanthanide-mediated reduction reactions.

## Results and Discussion

### Choice of Chromophores

Nine chromophores (**L1**–**L9**) with absorptions ranging from the UV (**L1**–**L4**) region to the visible (**L5**–**L9**) region were selected. With the exception of **L1** and **L2**(27) (which are readily synthesized on 100–700 mg scales) all **L** are commercially available. **L** displays a variety of Ln(III)-coordinating properties. Complexes of multidentate **L1** and **L2** can be synthesized and isolated.^27^ Here, we used simple mixtures of **L** and Ln(OTf)_3_ in DMF. DMF solvates Ln(III) efficiently,^41,42^ therefore at most only weak interactions were expected between **L** and Ln(III). The absence of ground-state **L** and Ln(III) association does not preclude excited-state quenching leading to Eu(II).^43^

### Spectroscopy

**Figure 2:**
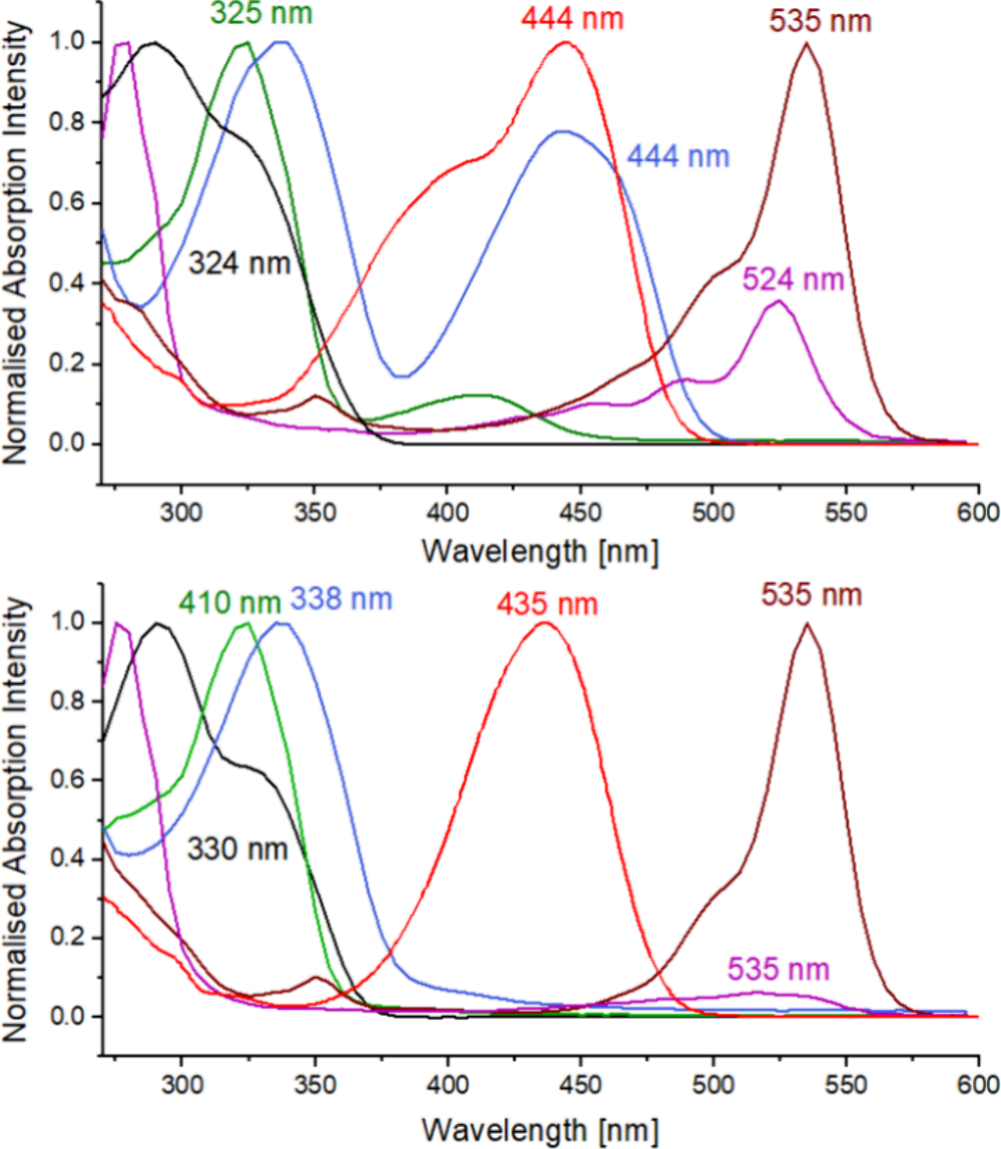
Normalized UV–vis absorption spectra of the new chromophores (top) and chromophore-Eu(OTf)_3_ combinations (1:1, bottom); **L4** (black), **L5** (green), **L6** (blue), **L7** (red), **L8** (purple), and **L9** (brown) in DMF; [**L4**–**L8**] = 17 μM, [**L9**] = 3.4 μM.

The photophysical properties of **L1**–**L9** were evaluated in DMF using UV–vis absorption and steady-state and time-resolved emission spectroscopy (Tables 1, 2, and Figures 2, S1–S26). DMF was used as a solvent as it solubilizes every **L**, and was previously used in Ln(II) photocatalysis.^27^ The absorption spectrum of **L4** is in the UV region with only a small tailing into the visible part (λ_max_ = 290 and 324 nm). **L5**–**L9** all have significant absorptions in the visible region. Their lowest-energy absorptions are located at λ_max_ = 412, 444, 444, 524, and 535 nm, respectively. At the output wavelength of the blue LED commonly used in photoreactors (λ_em_ = 450 nm) **L5**, **L7**, and **L9** absorb strongly, **L6** and **L8** moderately well, while absorptions of **L1**–**L4** at this wavelength are due to their UV-located bands tailing into the visible. Notably, even the weak absorptions of **L1**–**L3** have been suitable for visible-light photocatalysis.

**Table 1:** Photophysical Properties of **L1**-**L9** in DMF

L	ε_450_ [M^–1^cm^–1^]^a^	λ_em_ [nm]^b^	*E*_T_ [cm^–1^]^c^	*E*_ox_^L^ (V vs NHE)^d^	Δ*G*_PeT_^S^ [eV]	Δ*G*_PeT_^T^ [eV]
**L1**		412^e^	19,100	1.10 (vs Fc/Fc^+^)^e^	–0.83	–0.03^f^
**L2**		418^e^	19,100	1.10 (vs Fc/Fc^+^)^e^	–0.92	–0.15^f^
**L3**		385^e^				
**L4**		416	17,950	1.27	–1.37	–0.51
**L5**	10,800	464	18,400	1.62	–0.594	–0.06
**L6**	650	498	17,400	1.78	–0.375	–0.09
**L7**	21.700	493	16,750	1.26	–0.952	–0.36
**L8**	820	545	15,800	1.56	–0.103	0.36
**L9**	7760	562	16,650	1.35	–0.056	–0.10

a ε calculated at λ = 450 nm.

b Calculated from emission spectra, excited at λ_ex_ = 325, 325, 337, 435, 490, and 499 nm for **L4**, **L5**, **L6**, **L7**, **L8**, and **L9**, respectively, [**L4–L8**] = 17 μM, [**L9**] = 3.4 μM in DMF.

c Determined from the emission spectra of a mixture of **L** and Gd(OTf)_3_ recorded at 77 K.

d Where antenna oxidation was irreversible under the experimental conditions the oxidation potential is used.

e Taken from ref (27).

f Calculated from data reported in Ref (27).

**Table 2:** Fluorescence Quantum Yields of **L1**–**L9** with and without Ln(III) Salts in DMF

L	Φ_L_ [%]^a^	Φ_L_^Gd^ [%]^a^	Φ_L_^Eu^ [%]^a^	Φ_L_^Sm^ [%]^a^
**L1**^b^		8.1	7.6	6.6
**L2**^b^		7.2	1.5	4.3
**L3**				
**L4**	0.033	0.076	c	c
**L5**	11	12	11	9.8
**L6**	30	36	34	40
**L7**	94	89	69	62
**L8**				
**L9**	83	99	90	92

a Determined using an external reference, see ESI for details.

b Ref (27).

c Not determined due to low emission intensity. Φ_L_^Ln^: dye-based emission in the presence of 1 equiv of Ln(III) ions at [**L**] = [Ln(III)] = [**L4**] = 10 μM, [**L5**] = 7 μM, [**L6**] = 15 μM, [**L7**] = 2 μM, [**L9**] = 1.67 μM.

The absorption spectra of **L1**-**L9** were recorded in 3–17 μM solutions in the presence of equimolar quantities of Eu(OTf)_3_ (Figures 2, S6, S8, S10, S12, S14, S16, S18–S23). The addition of Eu(III) decreased the absorption intensity of **L8** and increased that of **L7**, and peaks at λ_abs_ = 410 nm, λ_abs_ = 444 nm and λ_abs_ = 395 nm disappeared with the emergence of peaks at 378, 405, and 435 nm in the case of **L5**, **L6,** and **L7**, respectively. The impact of Ln(III) binding on the absorption spectra of **L4** and **L9** was small (Figures S18 and S23). In less-solvating acetonitrile-*d*_3_^41^ the loss of the carboxylic proton and shift in the ^1^H NMR spectra of **L4** and **L7** in the presence of Eu(III) ions were consistent with **L**-Eu(III) association (Figures S120–S124). Solid-state structure analysis of single crystals of **L4**-Eu(III) revealed the formation of a coordination polymer. The Eu(III) center is surrounded by four **L4**-ligands in three different coordination modes: a) one 1κ^2^(O, O’) bidentate coordination via the carboxylate, b) one 1κ^1^(O):2κ^1^(O”) bidentate coordination involving the carboxylate and the carbonyl, and c) two μ-1κ^1^(O): 2κ^1^(O’): 2κ^1^(O”) bridging ligands via the carboxylate and the carbonyl site. The direct coordination environment of the Eu(III) center is saturated with further water molecules giving a distorted tricapped trigonal prismatic coordination environment (Figure S125) Additional water molecules link two of the 1D-polymer strains together resulting in a 1D double-stranded coordination polymer (Figure S126). Observed differences in the IR spectra (recorded as KBr pellets) with and without Ln(OTf)_3_ include the 3000–3600 cm^–1^ region, the C=O stretching vibrations at 1550–1750 cm^–1^, and the carboxylic acid C–O stretch at 1100–1300 cm^–1^ (Figures S105–S109, Table S2).^44^

Steady-state fluorescence spectra were recorded in the absence and presence of Ln(III) ions (Ln = Gd, Eu, and Sm). **L1**–**L9** were fluorescent upon excitation into λ_max_. Emission maxima were observed at λ_em_ = 412, 418, 385, and 416 nm for **L1**-**L4** respectively, at λ_em_ = 394 and 464 nm for **L5**, at λ_em_ = 428 and 498 nm for **L6**, and at λ_em_ = 493, 545, and 562 nm, for **L7**–**L9**, respectively (Table 1). Fluorescence quantum yields (Φ_L_) were determined at [**L**] = [Ln(III)] using quinine sulfate as the reference for **L4**–**L6**, and coumarin 153 for **L7** (Table 2). Values for **L1** and **L2** were reported previously. Under these conditions, Φ_L_ were 11, 30, 94, and 83% for **L5**, **L6**, **L7,** and **L9**. All Ln(III) ions can quench the first singlet excited state (S_1_) by increasing the rate of intersystem crossing via a heavy atom effect. Photo- and redox-active Ln(III) ions (e.g., Eu or Sm) can additionally quench S_1_ via energy- and electron transfer, decreasing Φ_L_ compared to what is seen in the presence of nonphotoactive and redox-inactive Gd(III). The separation of the effects of energy and electron transfer is not possible with the methods discussed here.^45^ At higher **L** and Ln(III) concentrations the fluorescence quantum yield was lower (Figures S62–S67), which is consistent with the quenching of the **L** excited state by Ln(III).

The energies of the lowest triplet excited states (*E*_T_) of **L** were determined from the low-temperature luminescence spectra in the presence of Gd(OTf)_3_. The first triplet excited state (T_1_) of **L1** and **L2** were at 19100 cm^–1^ (Figures S24 and S25), those of **L4**, **L5**, **L6**, **L7**, **L8**, and **L9** were less energetic, and were located between 18,400 (**L5**) and 15,800 cm^–1^ (**L8**, Table 1). The addition of Eu(OTf)_3_ quenched **L4** fluorescence and yielded characteristic Eu(III) luminescence (Figure S7) due to energy transfer from **L4** to Eu(III). The fluorescence intensities of **L5**–**L9** were greatly reduced in the presence of Eu(III). Eu(III) emission was not seen even under Ar, which suggests that S_1_-mediated energy transfer was not responsible for the lower Φ_L_ under these conditions.

The fluorescence emission of **L7** was monitored in the presence of increasing amounts of Ln(III). The **L7** fluorescence intensity increased until ∼0.7 equiv of Eu(III) was added to the solution, after which the emission was gradually quenched. In DMF at the λ_ex_ = 440 nm wavelength, the absorption of a 1:1 mixture of Eu(III) and **L7** was 26% more intense than for the corresponding **L7** solution, which explains the initial increase in fluorescence. In acetonitrile, no such changes in absorption occur, and titration of **L7** with Gd(OTf)_3_, Eu(OTf)_3_, and Sm(OTf)_3_ (Figures S27–S34) gradually decreased the fluorescence intensity of **L7** by 41, 70, and 79%, respectively. A solution of **L7** was titrated with Gd(OTf)_3_ (Figures S32 and S33). The obtained Stern–Volmer plot was not linear, suggesting some ground-state association between **L7** and Gd(III). A similar titration with Sm(OTf)_3_ produced a curve upwardly deviating from the linear, which is consistent with both static and dynamic quenching occurring in the system (Figures S30 and S31). Very weak Eu(III) emission and no Sm(III) emission were observed by luminescence spectroscopy even under Ar (Figures S35 and S36); thus, energy transfer does not appear to be efficient from **L7** to Eu(III) or Sm(III).

### Electrochemistry

**Figure 3:**
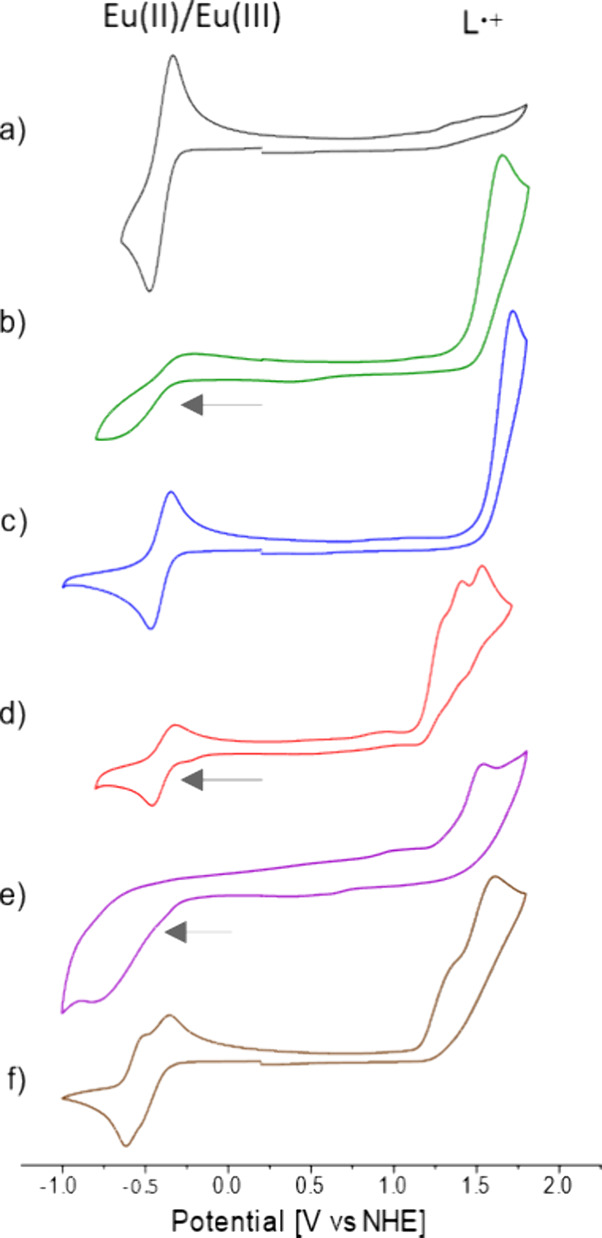
(a) Cyclic voltammograms of (a) Eu(OTf)_3_ + **L4** (black line), (b) Eu(OTf)_3_ + **L5** (green line), (c) Eu(OTf)_3_ + **L6** (blue line), (d) Eu(OTf)_3_ + **L7** (red line), (e) Eu(OTf)_3_ + **L8** (purple line), and (f) Eu(OTf)_3_ + **L9** (brown line) [**L**] = [Eu(OTf)_3_] = 5 mM in DMF (0.1 M TBAPF_6_), GC working electrode, Pt wire counter electrode, Ag/AgCl reference electrode, and reported vs NHE.

The ground state oxidation potentials of **L4**-**L9** (*E*_ox_^L^(**L**/**L**^+^·)) were determined in DMF using cyclic voltammetry in the absence and presence of Eu(OTf)_3_ (5 mM, 1.0 equiv) (Figures S95–S99). Values are reported vs NHE. At 100 mV/s scan rate all **L** except **L4** underwent irreversible oxidation within the solvent window. **L4** did not show significant oxidation in the scanned range. **L7** showed multiple oxidation events at 1.26, 1.38, and 1.50 V vs NHE. In acetonitrile, **L7** underwent a single reversible oxidation at 1.31 V vs NHE, while the oxidation of **L5** remained irreversible in this solvent, and shifted 0.21 V anodically (Figures S100–S103). The Ln(III)/Ln(II) reduction potential (*E*_red_^Ln^(Eu^II^/Eu^III^)) is strongly dependent on the coordination environment of the ion.^11,29,38,46^ Ligand binding can change *E*_red_^Ln^(Eu^II^/Eu^III^) compared to a solvated ion.^30^ The *E*_red_^Ln^(Eu^II^/Eu^III^) values were determined in the presence of 1.0 equiv **L4**-**L9** (Figure 3) as −0.48, −0.60, −0.47, −0.46, −0.76, and −0.61 vs NHE, respectively. These values are comparable to what has been reported for *E*_red_^Ln^(Eu^II^/Eu^III^) in DMF,^47,48^ and are consistent with at most weak Eu-**L** interactions in this strongly solvating medium.

The driving forces for PeT from the S_1_ and T_1_ states of **L4**-**L9** were calculated from *E*_red_^Ln^(Eu^II^/Eu^III^), *E*_ox_^L^(**L**/**L**^+^·), and *E*_S_ (S_1_ energy) or *E*_T_ using eq 1. All of the S_1_ states were sufficiently reduced to yield Eu(II). The possible exception is **L9** with Δ*G*_PeT_ close to 0 eV or even >0 eV due to the error of the measurements. **L4** and **L7** have T_1_ states that may be able to reduce Eu(III) (Table 1). *E*_red_^Ln^(Sm^II^/Sm^III^) was −0.85 V vs NHE in the presence of **L7**, and thus Sm(III) photoreduction by **L7** is possible.
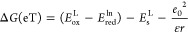
1

### Photochemical Ln(II) Generation

Next, the presence of Eu(II) was directly probed in irradiated mixtures of Eu(III) and **L**. Eu(II) is paramagnetic,^8^ and its photophysical and electrochemical properties depend on its environment.^11,46,49^ Unlike Ln(III) ions that have weak f–f absorptions in the near UV and visible range, Ln(II) species display broad absorptions due to MLCT or 4f–5d transitions^50^ Many Ln(II) complexes are luminescent^51^ in solution^10,49,52−54^ or the solid state;^55,56^ the luminescence lifetimes in solution are in the low ns to μs range.^57^ Eu(II) is EPR active. Mixtures of Eu(OTf)_3_ and **L** (**L4**–**L9**, 0.3 mM) were irradiated for up to 1.5 h in the presence of *N*,*N*-diisopropyl ethylamine (5.7 mM DIPEA), the role of the latter was to regenerate **L** and thus allow for the build-up of Eu(II) by closing down back electron transfer from Eu(II) to **L**^+·^. The UV–vis absorption and emission and EPR spectra of the irradiated mixtures were then recorded.

### UV–vis Absorption and Emission Spectroscopy

Eu(II) complexes absorb in the UV–vis region, and luminescence is often located in the visible. Emission is highly sensitive to the ligand, solvents, and counterions.^58,53,59^ As an example, Eu(OTf)_2_ emits at λ_em_ = 445 nm in THF and at λ_em_ = 483 nm in dimethoxyethane (DME), the corresponding values for EuI_2_ are λ_em_ = 440 and λ_em_ = 516 nm.^53^ Absorption maxima are minimally affected by the solvent although the impact on molar absorption coefficients can be substantial.^53^ The absorption spectrum of Eu(OTf)_2_ in DMF at a concentration of 1 mM (Figure S68) shows a broad band with a maximum at λ_em_ = 333 nm; this value did not change in the presence of added acetate. Excitation at λ_ex_ = 335 nm yielded a broad band with two maxima at λ_em_ = 437 and 464 nm (Figure S69). In the presence of additional DIPEA and acetic acid (added as a nonabsorbing mimic to carboxylate-carrying **L**) the emission band became a single broad band with λ_em_ = 460 and (Figures S70 and S71). A mixture of Eu(OTf)_3_ and acetic acid (1 mM each) in CH_3_CN was reduced electrochemically by applying a voltage of −0.35 V (vs Ag/AgNO_3_), and its absorption was followed during the reduction (Figure S90). Strong initial absorption at λ_abs_ = 279 nm with a shoulder at λ_abs_ = 360 nm decreased during the reaction, and two new bands centered at λ_abs_ = 320 nm (with a shoulder at 370 nm), and at λ_abs_ = 433 nm emerged. The former matches the reported λ_abs_ of Eu(OTf)_2_ in DME and THF^53^ and is similar to the absorption seen in DMF. Excitation at λ_ex_ = 370 nm yielded characteristic Eu(III) luminescence along with a broad emission band centered at λ_em_ = 462 nm assigned to Eu(II) (Figure S91), which is again similar to the value obtained in DMF.

Mixtures of Eu(OTf)_3_ or Sm(OTf)_3_ and **L** (**L4-L9**) (1:1, 0.3 mM in DMF) were irradiated in the presence of DIPEA (5.7 mM) (Figures S72–S89). At this concentration, changes in the chromophore absorptions close to their maxima could not be discerned. Therefore, observations were restricted to less intense features. Irradiation of a mixture of **L4** and Eu(OTf)_3_ with blue light for up to 30 min resulted in the emergence of a small shoulder centered at ∼400 nm (Figure S72). **SmL4** showed similar changes, although the shape of the shoulder was slightly different (Figure S75). Direct excitation of Eu(III) at λ_abs_ = 393 nm indicated that [Eu(III)] decreased during the irradiation experiment by ∼70% of its original value (Figure S73). However, the opening of the cuvette and the addition of benzyl bromide to the mixture did not restore the original Eu(III) emission. Therefore, at least some of the Eu(III) luminescence decrease is likely due to the accumulation of the visible-absorbing species that may not sensitize Eu(III) emission.

When **L5** was irradiated in the presence of Eu(III) for up to 30 min, the absorption spectrum of the sample remained the same (Figure S76). Continued irradiation for an additional 1 h resulted in the emergence of a small shoulder at λ_abs_ = 442 nm. The emission spectrum of the sample contained only a band with λ_em_ = 412 and 460 nm, the same wavelength as the **L5** fluorescence emission in the absence of Eu(OTf)_3_ (Figure S77). The EPR spectrum of **EuL5** was consistent with low levels of Eu(II) formation, and substantial chromophore-based photocatalytic reactivity was seen (vide infra).

The absorption of **EuL6** decreased in the 370–450 nm range to a shoulder centered at 400 nm after 15 min of irradiation. This change was reversed by the opening of the cuvette to the air, which returned most of the original spectrum (Figure S78). The absorption of **SmL6** underwent a somewhat similar but irreversible change (Figure S80). The emission spectrum (λ_ex_ = 360 nm) showed a structured band with two maxima at λ_em_ = 444 and 497 nm (Figure S79). The emission was different from that of the starting mixture but essentially identical to that of **SmL6** after irradiation (Figure S81), and therefore not due to Eu(II). Sm(III) luminescence appeared in the **SmL6** solution after irradiation (Figure S81), which is consistent with **L6** undergoing a (possibly Ln(II)-promoted) change to a derivative that can sensitize Sm(III) emission.

The irradiation of **EuL7** changed the 300–350 nm range of its absorption spectrum (Figure S82). Excitation at λ_ex_ = 330 nm or λ_ex_ = 360 nm yielded intense fluorescence, which was accompanied by typical Eu(III) luminescence (Figure S83). The proportion of the broad fluorescence band and the Eu(III) emission increased with time for both excitation wavelengths and the intensity of the Eu(III) emission decreased. While a diminished Eu(III) luminescence is consistent with Eu(III) reduction to Eu(II), it can also be the result of other processes, such as Eu(III) precipitation or the degradation of an initially present chromophore that sensitized Eu(III) luminescence.

**Figure 4:**
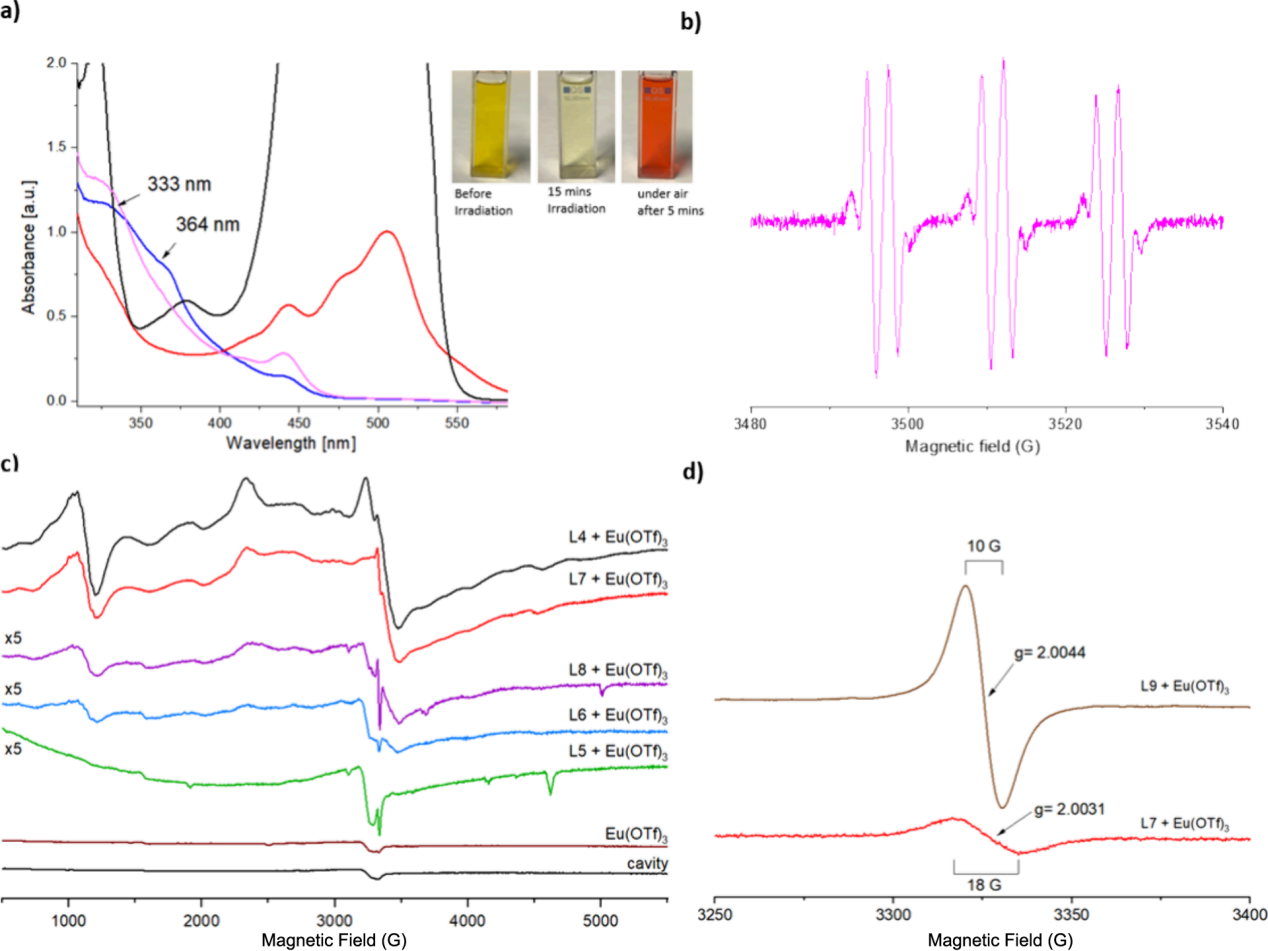
Photochemical Eu(II) formation. (a) Absorption spectra of a mixture of **L8** and Eu(OTf)_3_ (0.3 mM in DMF) in the presence of DIPEA (5.7 mM) before irradiation (black), after 15 min irradiation (blue), after 30 min irradiation (pink), and exposure to air (red). The spectrum is truncated to better show the lower-intensity 333 and 364 nm-bands. (b) EPR spectrum of a mixture of **L7**, Eu(OTf)_3_ and PBN (*N*-tert-butyl-α-phenylnitrone, 1 mM in DMF) after 12 h of irradiation at room temperature. *T* = 293 K, microwave power: 2 mW, modulation amplitude: 1 G. (c) EPR-signals from equimolar mixtures of Eu(OTf)_3_ and **L** (1 mM in DMF) after irradiation for 30–60 min with blue LED. Spectra from **L5** and Eu(OTf)_3_ (green), **L6** and Eu(OTf)_3_ (blue), and **L8** and Eu(OTf)_3_ (purple) are multiplied by 5 for clarity. *T* = 10 K, microwave power: 2 mW, Modulation amplitude: 19.5 G. (d) Organic radicals in the EPR samples of **L9** and Eu(OTf)_3_ (brown), and **L7** and Eu(OTf)_3_ (red) recorded in DMF after irradiation for 30 min with blue LED. *T* = 10 K; microwave power: 2 μW; modulation amplitude: 3 G.

**EuL8** absorbed strongly at λ_abs_ = 506 nm before irradiation (Figures 4a and S86); after 15 min, the solution became a very light color and the 506 nm peak disappeared. Two small new peaks at λ_abs_ = 333 nm and λ_abs_ = 364 nm emerged; the latter decreased upon further irradiation. These changes were partially reversible, and some visible absorption was recovered upon the opening of the cuvette to air. Notably, the absorption spectrum of **L8** is sensitive not only to the presence of Eu(III) but also to DIPEA (Figure S22). A mixture of Eu(OTf)_3_ and **L8** absorbs only minimally in the visible, while a ternary mixture containing DIPEA absorbs strongly. The consumption of DIPEA and conversion to a weaker base or to a nonbasic product under the photochemical reaction is a possible reason for the incomplete recovery of the **L8** absorption. Additionally, the dianion of **L8** (fluorescein) is known to decompose when excited by λ_ex_ = 400 nm light by loss of an electron and decarboxylation.^60^ An analogous pathway may operate in the presence of an electron acceptor (Eu(III)), yielding decarboxyfluorescein.

Several **L** species were sufficiently reductive in their excited states to generate Sm(II) from Sm(III). In the presence of Sm(OTf)_3_ the absorptions of both **L6** and **L7** decreased (Figures S80 and S84). Most of this intensity loss was irreversible, i.e., the original absorption spectra were not restored when the irradiated samples were opened to the air. The initial Sm(II) irradiation product is a strong reductant and may react with the chromophores, thus altering the chromophore-associated absorptions.

### EPR Spectroscopy

PeT to Eu(III) from a photoexcited coumarin yields two EPR active species, Eu(II) and the chromophore radical cation. Both have been observed at low temperatures in the case of **L1**.^27^**L4**–**L9** were irradiated with a blue LED in the presence of an Eu(III) salt, and the EPR spectra of the products were recorded (Figures 4b–d, S92–S94). Solutions containing equimolar quantities of Eu(OTf)_3_ and the appropriate **L** were prepared (1 mM in DMF) in a glovebox. The sample was irradiated for 30–60 min, and the reaction mixture was plunged into liquid N_2_ immediately after irradiation. The EPR spectra were collected at 10 K. The samples containing both Eu(III) and **L** (except **L5**) showed broad EPR signals after irradiation with a transition at *g* = 2.0 and features from 500 to 5500 G. The intensity of the signal was lower in the case of **L6** and **L8** (Figures 4c and S92) and higher for **L9** (Figure S93). These signals were attributed to Eu(II) and are expected to be similar in all the samples irrespective of the chromophores.^27^ This Eu(II) EPR signal disappeared when a Eu(II)-quencher (benzyl bromide) was added to the solution. No EPR signal was observed for **L5** + Eu(OTf)_3_ (Figure 4c).

The samples also display EPR signals from organic-type radicals at *g* = 2.003–4, the strength and shape of which vary significantly (Figure S94). For example, this signal is ∼10 G wide (peak-to-through) in the sample containing **L9** and Eu(OTf)_3_, while the only other relatively strong radical signal, from the sample containing **L7** and Eu(OTf)_3_, is wider, ∼18 G (Figures 4d and S94). This means that the organic radicals originate from different species in the samples, which is consistent with the signals originating from different electron donors (i.e., **L9** or **L7**). Additionally, when the EPR spectrum of Eu(OTf)_3_ was recorded in the absence of **L** under similar conditions, no EPR signals were observed, suggesting that Eu(III) in the other samples was reduced to Eu(II) by the excited states of **L** (Figure 4c). *N*-tert-butyl-α-phenylnitrone (PBN), which itself is EPR inactive, can form a stable radical species in the presence of organic radicals. Irradiation of an equimolar solution of **L7** + Eu(OTf)_3_ containing the radical quenchers PBN showed the emergence of an N-based radical from a PBN adduct (Figure 4b).

### Reactivity

**Scheme 1:**
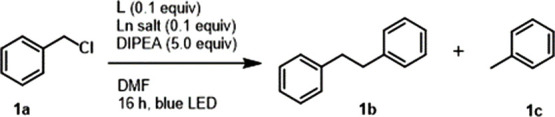
Benzyl Chloride Reduction with **LnL** Photocatalysts

Having shown that Eu(III) can be reduced photochemically by a variety of chromophores, chromophore-Ln(III) combinations were evaluated as photocatalysts. The reactivities of **L4**-**L9**-Ln(III) combinations were explored under previously optimized conditions (Table 3).^27^ Benzyl chloride (**1a**, 1 equiv, 0.087 mmol) in DMF was irradiated for 16 h with blue LED in a solution containing 0.10 equiv of **L**, 0.10 equiv of Eu(OTf)_3_, and 5.0 equiv of DIPEA as the sacrificial electron donor (Scheme 1), and the amount of bibenzyl (**1b**) was determined by GC-MS. Importantly, conditions were not optimized individually for the chromophore-Eu(III) combinations, and no effort was made to identify substrates that each **LnL** was best suited for. Our aim was to identify meaningful differences in reactivity and to provide further evidence for the presence of the Ln(II) intermediate.

**Table 3:**
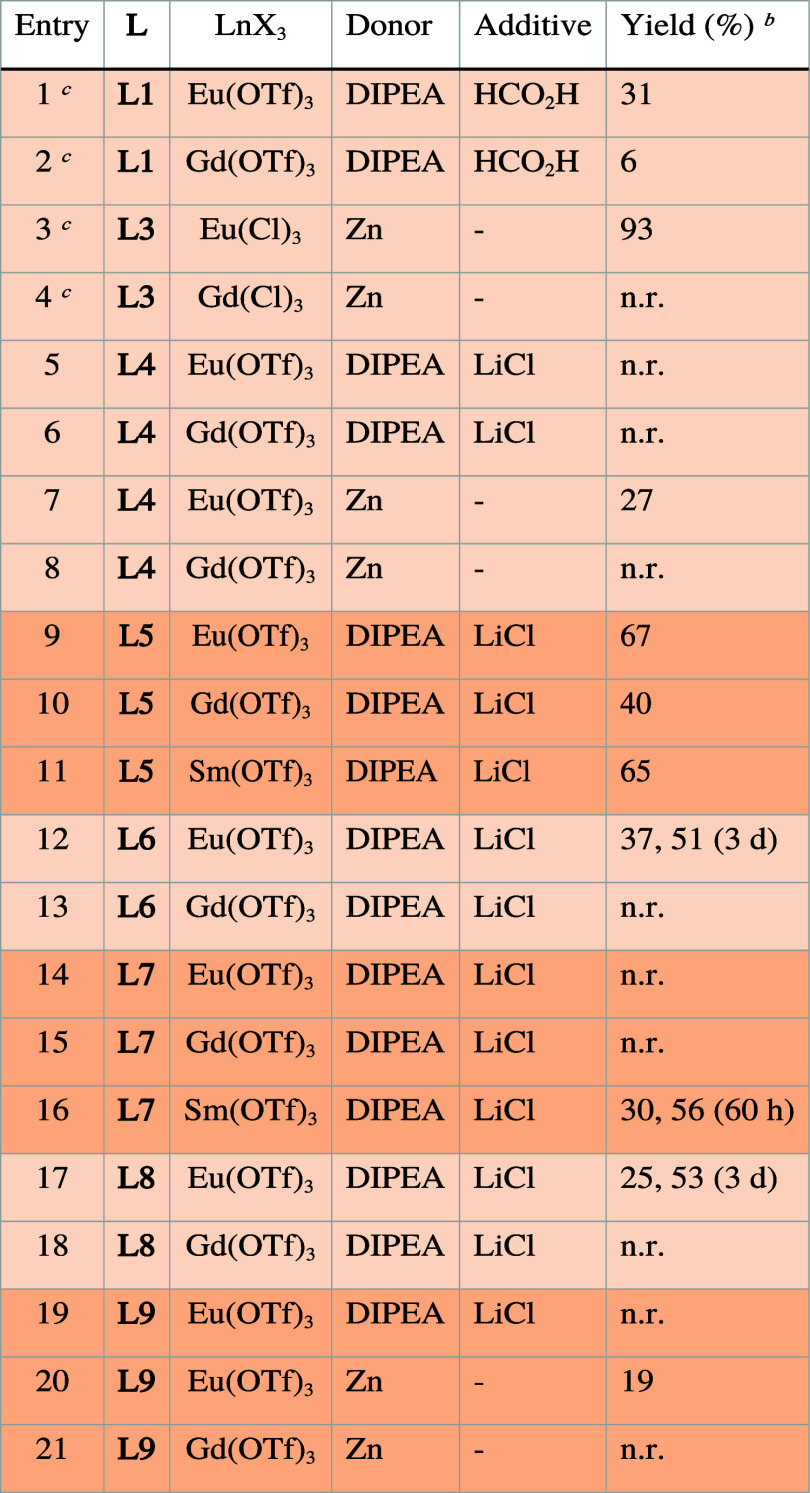
Benzyl Chloride Reduction Using **LnL** without Added Water^a^

a Results are the average of two independent experiments, 5 equiv of LiCl was used unless indicated otherwise. **1a** (1.0 equiv, 0.087 mmol), **L** (0.1 equiv, 0.0087 mmol), Eu(OTf)_3_ (0.1 equiv, 0.0087 mmol), DIPEA (5.0 equiv, 0.435 mmol, 76 μL), LiCl (5.0 equiv, 0.435 mmol), Zn (if applicable, 1.0 equiv, 0.087 mmol), DMF (1 mL).

b GC-MS yield calculated from the calibration curve.

c From ref (27).

**EuL6**, **EuL8**, and **EuL9** reacted slowly under the above conditions, and **EuL4** and **EuL7** were unreactive. **EuL3** has previously yielded toluene selectively with Zn as a sacrificial reductant in 79% yield.^27^**EuL4** and **EuL9** catalyzed **1a** reduction to **1b** with Zn as the sacrificial reductant, although their efficiencies remained modest (entries 7 and 20). The yield with **EuL6** (37%) and **EuL8** (25%) could be increased to 51 and 53%, respectively, by extending the reaction time to 3 days (entries 12 and 17), which suggests that these catalysts are stable under the reaction conditions. Some of the low reactivity is likely due to poor absorption at the excitation wavelength (**L4**, **L6**, and **L8**, Table 1). Additionally, **L4** is a good sensitizer of Eu(III) luminescence (Figure S7), and energy transfer competes with electron transfer. L9* is the least-reducing form of L*. **L4**, **L6**, **L8** and **L9** were unreactive in combination with nonreducible Gd(III), thus the reactions proceeded via the formation of Ln(II). Reactions did not take place in the dark, which shows that the sacrificial donors do not directly reduce Eu(III).

**EuL5** was a good catalyst for **1a** reduction (entry 9), however, control experiments showed that so was **GdL5**. Yields were higher with **EuL5** than with **GdL5** (67% vs 40%, respectively, entries 9, 10). Aminocoumarin photocatalysts can promote the reductions of C=X (X = O, N) and C–Br bonds to effect pinacol couplings and aldehyde α-alkylations.^61^L5* can clearly reduce **1a** directly. The difference in yield suggests that there may be an Eu(II)-mediated pathway operating parallel to the L5*-mediated one. This result highlights the importance of control experiments with nonphotoactive and redox-stable Ln(III) to enable unambiguous assignment of the mechanistically relevant reductant.

Our previous study showed that 20% water was needed to shift the absorption of **L2** into the visible region, which in turn enabled excitation by a blue LED (Figure S3). In the presence of 20% water **L1** and **L2** afforded **1b** in 31 and 56% yield, respectively.^27^ Water can also play important mechanistic roles. Ln(II)–OH_2_ is a proton-coupled electron transfer agent,^26,62−64^ and the binding of water alters the Ln(II) reduction potential.^65^ Therefore, the effect of added water on the reactions was evaluated (Table 4).

**Table 4:**
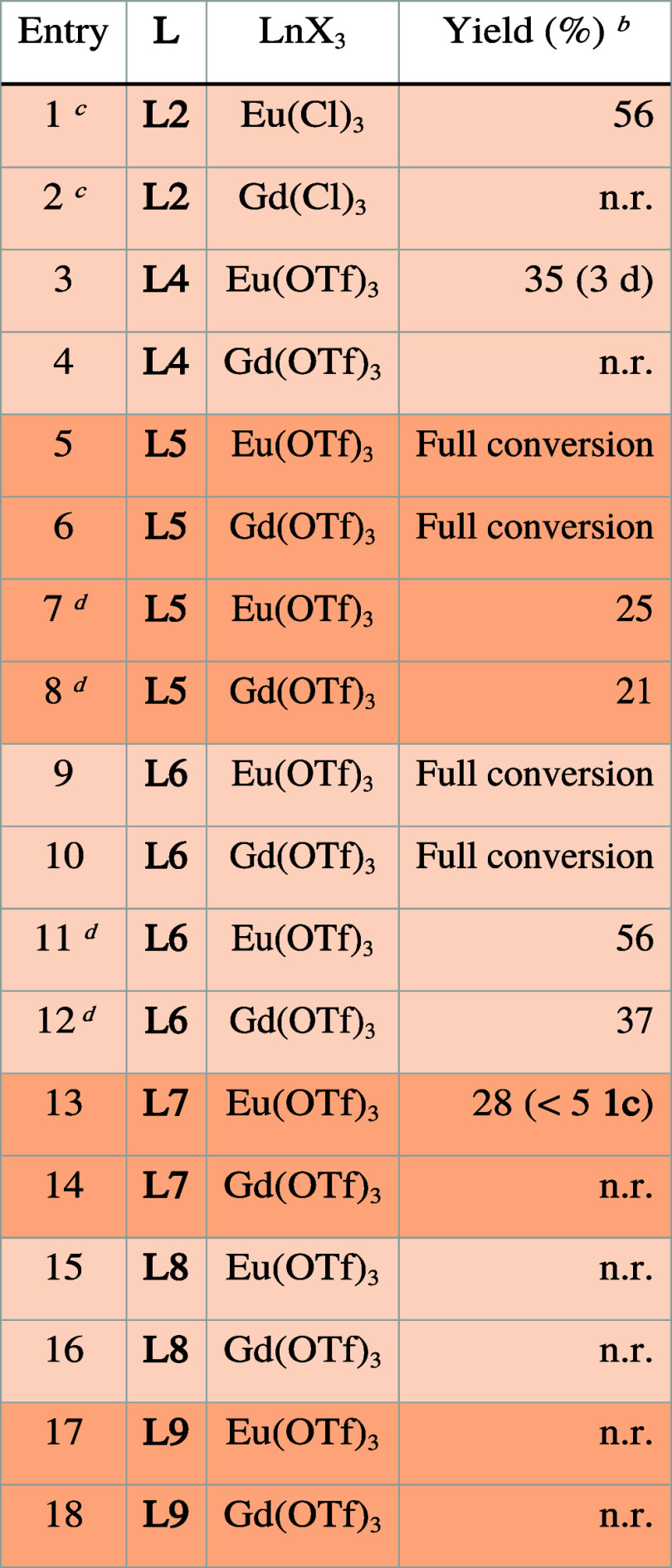
Benzyl Chloride Reduction Using **LnL** with Added Water Volume^a^

a Results are the average of two independent experiments, **1a** (1.0 equiv, 0.087 mmol), **L** (0.1 equiv, 0.0087 mmol), Eu(OTf)_3_ (0.1 equiv, 0.0087 mmol), DIPEA (5.0 equiv, 0.435 mmol), LiCl (5.0 equiv, 0.435 mmol), DMF:water (4:1, 1 mL).

b GC-MS yield calculated from the calibration curve.

c From refs (27, 10) equiv each of DIPEA and LiCl were added.

d Reaction time 5 h.

The reactivities of **EuL8** and **EuL9** were not impacted by water (entries 15–18). **EuL4** afforded 35% **1b** in 3 days (Table 4, entry 3), even with DIPEA as the donor. In the presence of water, both **GdL5** and **GdL6** afforded, like **EuL5** and **EuL6**, complete conversion of **1a** (entries 5, 6, 9, and 10).

**EuL7** afforded **1b** in 28% yield along with less than 5% toluene (entry 13, Table 4) only in the presence of water (Table 4), and **GdL7** was inactive. The absorption spectrum of **EuL7** was identical with and without water; therefore, the role of water is not to enhance light absorption. Interestingly, **SmL7** was active even in the absence of water. However, small amounts of benzyl alcohol were observed in the reaction mixture (Figure S116), and therefore, we cannot exclude that reactivity was facilitated by the water already present in the hygroscopic Sm(III) salt. An interesting observation is the continued activity of **SmL7** under photocatalysis conditions for up to 60 h, which is different from the rapid irreversible change it undergoes in the absence of a substrate.

## Conclusions

The ability of a variety of organic chromophores to photochemically reduce Eu(III) to Eu(II) was investigated. The chromophores were either readily synthesized following previously reported procedures^27^ or were commercially available. Their absorptions ranged from the UV region to >500 nm. Several absorbed well at the output of blue LEDs commonly used in photoreactors. Changes in the UV–vis absorption spectra and in the fluorescence quantum yields of the chromophores upon the addition of reducible Eu(III) vs redox-inert Gd(III), along with cyclic voltammetry data, indicated that in their excited states, several chromophores were sufficiently reducing to transfer an electron to Eu(III), and even to the less reducible Sm(III). The possibility of electron transfer was supported by the calculated driving forces for photoinduced electron transfer. The photochemically formed Eu(II) catalytic intermediate was characterized by EPR spectroscopy: broad EPR signals assigned to Eu(II) were observed at 10 K. UV–vis absorption spectroscopy of the irradiated mixtures of **L** and Eu(III) or Sm(III) showed a variety of outcomes. In the absence of a substrate, the absorption of **EuL6** underwent reversible changes and that of **EuL7** changed only minimally. The absorption and emission spectra of other **EuL** changed irreversibly, as did those of **SmL**.

**EuL** and **SmL** could reduce benzyl chloride. In the presence of a mild stoichiometric reductant (DIPEA or Zn), catalytic amounts of **LnL** could be used. The catalysts remained active for several days. The different chromophore-Ln combinations had significantly different reactivities that ranged from sluggish to good; many of the observations could be explained by differences in chromophore absorption and ease of Eu(II) formation. **LnL5** catalyzed benzyl chloride reduction independent of the lanthanide. These results show that a broad range of readily available light-harvesting chromophores can be paired with reducible lanthanides to obtain efficient photocatalysts for Ln(II)-mediated reduction reactions.

## Experimental Section

### Materials

**EuL1**, **EuL2**, **SmL1** and **SmL2** were synthesized following reported procedures.^27^ All other chemicals were purchased from commercial sources and used as received. DMF and MeCN were obtained from an Inert Puresolv solvent purification system. All solid chemicals were dried under a vacuum overnight before being used in the glovebox.

### General Procedures

^1^H NMR (400 MHz) and ^13^C NMR (100 MHz) spectra were recorded on a JEOL 400 MHz instrument. Chemical shifts were referenced to residual solvent peaks.

### UV–vis Absorption Spectroscopy and Luminescence Spectroscopy

All of the measurements were performed in DMF unless indicated otherwise. Quartz cells with 1 cm optical pathlengths were used for the room temperature measurements. The absorption spectra were recorded on a Varian Cary 100 Bio UV–visible spectrophotometer.

The steady-state emission and excitation spectra, Eu(III) luminescent lifetimes, and time-resolved emission and excitation spectra on the μs-ms time scale were recorded on a Horiba FluoroMax-4P instrument. All emissions were corrected by the wavelength sensitivity (correction function) of the spectrometer. All measurements were performed at room temperature, unless stated otherwise. Lifetimes were recorded 0.05 ms after pulsed excitation at the excitation maxima (λ_ex_) of the ligand by measuring the decay of the main lanthanide emission peak (Eu(III): 615 nm) The increments after the initial delay were adjusted between 0.2–20 μs depending on the lifetime to have a good sampling of the decay. The obtained data were fitted by single and double exponential decay models in OriginPro 9, and the most reliable value was chosen according to the adjusted *R*^2^ value and the shape of the residuals. A relative error of 10% is typically found among a series of measurements of the same sample.

Low temperature measurements were done in quartz capillaries (0.2 cm optical path length) at 77 K in DMF unless otherwise stated by immersion in a liquid N_2_-filled quartz Dewar.

*Caution! Extreme care should be taken in both the handling of the cryogen liquid nitrogen and its use in the Schlenk line trap to avoid the condensation of oxygen from air.*

### Quenching Studies

Quenching experiments were performed using a Horiba FluoroMax-4P spectrophotometer at room temperature in DMF/MeCN. The steady-state emission spectra of **L7** (3.4 μM) were recorded at λ_em_ = 400–700 nm, with λ_ex_ = 440 nm in the presence of increasing amounts of Ln(OTf)_3_, Figures S27, S28, S30, S32). The resulting data were plotted as *I*_0_/*I* (integrated spectra in the λ_em_ = 400–700 nm range) vs the concentration of Ln(OTf)_3_ (μM) (Figures S29, S31, S33).

### Quantum Yield Determination

Quantum yields were determined at room temperature using quinine sulfate (QS) in H_2_SO_4_ 0.05 M (Φ_ref_ = 0.59)^66^ for **L1-L6**, coumarin 153 in EtOH (Φ_ref_ = 0.55)^67^ for **L7**, and rhodamine 6G in EtOH for **L9** ((Φ_ref_ = 0.94)^68^ as a reference. The absorption at the excitation wavelengths was below 0.1 to avoid the inner filter effect, concentrations were [**L**] = [Ln(III)]; [**L4**] = 10 μM, [**L5**] = 7 μM, [**L6**] = 15 μM, [**L7**] = 2 μM, [**L9**] = 1.67 μM. Quantum yields were calculated according to eq S1, with Φ_s_ the quantum yield of the sample, Φ_ref_ the quantum yield of the reference, *I* the integrated corrected emission intensity of the sample (s) and of the reference (ref), *f*_A_ the absorption factor of the sample (s) and of the reference (ref) at the excitation wavelength, and *n* the refractive indexes of the sample (s) and of the reference (ref). The concentrations of the references were adjusted to obtain an absorbance matching with the maxima of the chromophore in a mixture of **L**:Ln(OTf)_3_. For the experiments with serial dilutions, the ratio of the **L** and Ln(OTf)_3_ was kept constant. The excitation wavelength where the absorption factors of the samples and of the reference were the same was chosen (i.e., where the absorptions are identical). The corrected emission spectra of the sample and reference standard were then measured under the same conditions over the spectral range as well as blank samples containing only the solvent. The appropriate blanks were subtracted from their respective spectra, and the antenna fluorescence was separated by fitting the section of the antenna emission exponentially overlapping the lanthanide emission (**L4**). The quantum yields were calculated according to eq S1. The given relative error on the quantum yields (δΦ = ΔΦ/Φ, where ΔΦ is the absolute error) takes into account the accuracy of the spectrometer and of the integration procedure [δ(*I*_s_/*I*_ref_) < 2%], an error of 0.59 ± 0.01 on the quantum yield of the reference QS [δ(Φ_ref_) < 2%], an error on the ratio of the absorption factors [δ(*f*_Aref_/*f*_As_) < 5%, relative to the fixed absorption factor of the reference QS] and an error on the ratio of the squared refractive indexes [δ(*n*_s_^2^/*n*_ref_^2^) < 1%, <0.25% around 1.333 for H_2_O and 1.430 for DMF on each individual refractive index], which sums to a total estimated relative error that should be δΦ_s_ < 10%. A limit value of 10% is thus chosen (see Supporting Information, eq S1).

The quantum yield of **L7** was also determined as follows. Serial dilutions of solutions of C153 and **L7** were prepared keeping *A* < 0.1. The fluorescence emission was recorded as described above, and *I* vs *A* was plotted (Figures S50–S55). The concentrations of the reference were not adjusted to obtain an absorbance matching the maxima of the chromophore in a mixture of **L**:Ln(OTf)_3_. In these experiments, ratios of **L7** and Ln(OTf)_3_ were kept constant.

Fourier transform infrared spectroscopy (FTIR). Measurements were taken on a PerkinElmer Spectrum One instrument. Spectra were recorded on dry samples by making a pellet using KBr with the ligand or complex (100:1). Blank was recorded with only a KBr pellet.

Gas chromatography was performed with mass spectrometry (GC-MS). Photoreactions were monitored by GC-MS (Agilent 7890A GC and 5975 MSD system). Samples were injected using split injection (1 μL injection volume; split ratio: 100:1; 250 °C inlet temperature; flow rate: 120 mL/min). The temperature rate was set to 20 °C/min, resulting in a 12.5 min total run time. He was used as a carrier gas at a flow rate of 1.2 mL/min. The column used was an Agilent 19091S-433:325 °C: 30 m × 250 μm × 0.25 μm (front SS-inlet: He; out: vacuum). Mass spectrometer: source temperature: 230 °C, quad temperature, 150 °C.

### Electrochemistry

Cyclic voltammograms (CV) were obtained at room temperature (∼20 °C) using an AUTOLAB PGSTAT 100 potentiostat or an AUTOLAB PGSTAT 204N potentiostat. The setup was equipped with a 3 mm glassy carbon (GC) working electrode, a Pt wire auxiliary electrode, and Ag/AgCl as a reference electrode. Measurements were done in anhydrous DMF and MeCN with NBu_4_PF_6_ (0.1 M) as the supporting electrolyte. The voltammograms were recorded by scanning first toward more negative potential values (reduction). A step-potential of −0.9 mV was used for 100 mV/s scan rates.

A solution of NBu_4_PF_6_ (0.1 M) in DMF/MeCN (2 mL) was added to the electrochemical cell. The working electrode was polished with 0.05 μm alumina on a polishing pad, washed with water and ethanol, and dried. This was repeated for each new sample. The three electrodes (GC working electrode, platinum wire auxiliary electrode, and Ag/AgCl reference electrode) were inserted into the cell setup followed by argon purging for 10 min, and a background scan was recorded with a scan rate of 100 mV/s, and two sweeps. The complexes were added to the solution (2–5 mM) and purged again for 10 min, and the sample was recorded.

Spectro-electrochemistry was performed in an argon-filled glovebox with a solution of Eu(OTf)_3_ and acetic acid (1.33 mM) in acetonitrile using NBu_4_PF_6_ (0.1 M) as the electrolyte. The three electrodes (carbon (mesh) as a working electrode and counter electrode and Ag/AgNO_3_ as a reference electrode) were used. A potential of −0.35 V (vs Ag/AgNO_3_) was applied for 30 min, and UV was recorded every 30 s during the measurement.

### EPR Spectroscopy

EPR measurements at room temperature were performed using a Bruker EMX Micro spectrometer, equipped with an ER 4119HS resonator. EPR samples were prepared in a 1 mm capillary. EPR parameters: microwave frequency, 9.86 GHz; modulation frequency 100 kHz. EPR measurements at 10 K were performed using a Bruker ESR-500 spectrometer, equipped with an ER 4122SHQ resonator, an ESR900 cryostat, and an Oxford ITC503 temperature controller. EPR parameters: microwave frequency, 9.38 GHz; modulation frequency, 100 kHz. All of the parameters are constant unless indicated otherwise.

### Photoreaction Setup

All reactions were performed in microwave vials equipped with a stirring bar, in a dry glovebox [O_2_ (<0.5 ppm), H_2_O (<0.5 ppm)] with an Ar atmosphere. The vials were charged with **1a** (1.0 equiv, 0.087 mmol), **L** (0.1 equiv, 0.0087 mmol), Eu(OTf)_3_ (0.1 equiv, 0.0087 mmol), DIPEA (5.0 equiv, 0.435 mmol, 76 μL), LiCl (5.0 equiv, 0.435 mmol), Zn (if applicable, 1.0 equiv, 0.087 mmol), and DMF (1 mL), or with 1a (1.0 equiv, 0.087 mmol), L (0.1 equiv, 0.0087 mmol), Eu(OTf)_3_ (0.1 equiv, 0.0087 mmol), DIPEA (5.0 equiv, 0.435 mmol), LiCl (5.0 equiv, 0.435 mmol), and DMF:water (4:1, 1 mL), and were then sealed with an electric black tape. A 40 W blue LED lamp (Kessil A160WE Tuna Blue, λ_max_ = ∼450 nm, set to the highest blue color and intensity) was used for irradiation. Reactions were stirred at 600–1000 rpm. GC–MS yield was determined using a calibration curve prepared from integrated peak areas of **1b** (0.18–1.25 mM), and **1c** (0.04–0.7 mM) solutions. For the full emission spectrum of the A160WE Tuna Blue light source see ref (69).

### Irradiation Experiments

All reactions were performed in quartz cuvettes loaded with Ln(OTf)_3_ (0.3 mM), **L** (0.3 mM), and DIPEA (5.7 mM) and DMF (3 mL) in a glovebox [O_2_ (<0.5 ppm). The vials were sealed with an electric black tape. A 40 W blue LED lamp (Kessil A160WE Tuna Blue, λ_max_ = ∼450 nm, set to the highest blue color and intensity) was used for irradiation for the indicated length of time. The absorption and emission were recorded as described above.

### Eu(OTf)_2_ Preparation

Method 1:^70^ To the stirred solution of EuI_2_ (4 mg, 1.0 equiv) in DMF (1 mL), AgOTf (4.8 mg, 2.0 equiv) was added. The clear dark yellow solution immediately turned into a sandy mixture. Stirring was continued for 20–30 min. The mixture was filtered using a syringe filter to get a pale yellowish solution. 250 μL of this solution was added to DMF to yield a 3 mL solution in a cuvette. DIPEA (260 μL, 0.5 mM) was added dropwise to acetic acid (85 μL, 0.5 mM) in DMF, and the resulting solution was added in the Eu(OTf)_2_-solution in DMF for spectroscopy.

Method 2:^71^ To a stirred solution of Eu(OTf)_3_ (5 mg, 1 equiv) in DMF (1 mL), Zn (22 mg, 40 equiv) was added. The mixture was stirred for 2–3 h. This mixture was filtered using a syringe filter to give a pale solution. A 250 μL sample of this solution was added to DMF to afford a total volume of 3 mL in a cuvette.

### X-ray Crystallography

Single crystals of **L4**-Eu(III) were obtained by slow evaporation of methanol layered with diethyl ether. A suitable crystal was selected and mounted using Fomblin oil on a fiber-loop on an XtaLAB Synergy, Single source (CuKα) diffractometer equipped with a HyPix detector. The crystal was kept at 100.00(10) K during data collection. Using Olex2,^72^ the structure was solved with the SHELXT structure solution program using Intrinsic Phasing and refined with the SHELXL^73^ refinement package using Least Squares minimization.

## Supporting Information Available

Additional photophysical, electrochemical, and NMR spectroscopic characterization, FTIR spectra for **LnL** and GC-MS traces for the reactions in Tables 3 and 4 (PDF)
