# Supplementary material for: Divalent Intermediates in Lanthanide-Based Photocatalysts: Spectroscopic Characterization and Reactivity
Source: Inorg Chem. 2024 Dec 23;64(1):594–605. doi: 10.1021/acs.inorgchem.4c03926 (PMC11734113; doi:10.1021/acs.inorgchem.4c03926)
Supplement: Supplementary file 1 — ic4c03926_si_001.pdf [file ic4c03926_si_001.pdf]

Supporting Information

Divalent intermediates in lanthanide-based photocatalysts: spectroscopic characterization and reactivity

Monika Tomar, Anders Thapper, Andreas Orthaber, K. Eszter Borbas*

Department of Chemistry, Ångström Laboratory, Box 523, Uppsala University, Uppsala, 75120, Sweden.

*eszter.borbas@kemi.uu.se

Table of Contents

1. Materials and methods ... 2

2. Photophysical characterization ... 5

Absorption and emission, and excitation spectra of L and LnL ... 5

Low-temperature spectra of GdL and L ... 17

Quenching experiments ... 20

Quantum yield determinations ... 25

Photophysical characterization of the irradiated Ln(III)-L mixtures ... 36

3. EPR spectroscopy ... 48

4. Electrochemistry ... 50

5. PeT driving force calculations ... 55

6. Photocatalysis ... 56

7. IR spectra ... 57

8. GC-MS traces ... 60

9. ^{1}H NMR spectra ... 66

10. X-ray crystallography ... 69

11. References ... 71

1. Materials and methods

Materials. EuL1, EuL2, **SmL1** and **SmL2** were synthesized following reported procedures.[1] All other chemicals were purchased from commercial sources, and used as received. DMF and MeCN were obtained from an Inert Puresolv solvent purification system. All solid chemicals were dried under a vacuum overnight before being used in the glovebox.

General Procedures. ^{1}H NMR (400 MHz), ^{13}C NMR (100 MHz) spectra were recorded on a JEOL 400 MHz instrument. Chemical shifts were referenced to residual solvent peaks.

UV-Vis absorption spectroscopy and luminescence spectroscopy. All the measurements were performed in DMF unless indicated otherwise. Quartz cells with 1 cm optical pathlengths were used for the room temperature measurements. The absorption spectra were recorded on a Varian Cary 100 Bio UV-Visible spectrophotometer.

The steady-state emission and excitation spectra, Eu(III) luminescent lifetimes, and time-resolved emission and excitation spectra on the µs–ms timescale were recorded on a Horiba FluoroMax-4P instrument. All emissions were corrected by the wavelength sensitivity (correction function) of the spectrometer. All measurements were performed at room temperature unless stated otherwise. Lifetimes were recorded 0.05 ms after pulsed excitation at the excitation maxima (λ_{ex}) of the ligand by measuring the decay of the main lanthanide emission peak (Eu(III): 615 nm) The increments after the initial delay were adjusted between 0.2–20 µs depending on the lifetime to have a good sampling of the decay. The obtained data were fitted by single and double exponential decay models in OriginPro 9, and the most reliable value was chosen according to the adjusted R^2 value and the shape of the residuals. A relative error of 10% is typically found among a series of measurements on the same sample.

Low temperature measurements were done in quartz capillaries (0.2 cm optical pathlength) at 77 K in DMF unless otherwise stated by immersion in a liquid N_2-filled quartz Dewar.

Caution! *Extreme care should be taken both in the handling of the cryogen liquid nitrogen and its use in the Schlenk line trap to avoid the condensation of oxygen from air.*

Quenching studies. Quenching experiments were performed using a Horiba FluoroMax-4P spectrophotometer at room temperature in DMF/MeCN. The steady-state emission spectra of **L7** (3.4 µM) were recorded at λ_{em} = 400–700 nm, with λ_{ex} = 440 nm in the presence of increasing amounts of Ln(OTf)$_3$, Figures S27, S28, S30, and S32). The resulting data were plotted as I_0/I (integrated spectra in the λ_{em} = 400–700 nm range) *vs* the concentration of Ln(OTf)$_3$ (µM) (Figures S29, S31 and S33).

Quantum yield determination. Quantum yields were determined at room temperature using quinine sulfate (QS) in H_2SO_4 0.05 M (Φ_{ref} = 0.59)[2] for **L1**–**L6,** coumarin 153 in EtOH (Φ_{ref} = 0.55)[3] for **L7**, and rhodamine 6G in EtOH for **L9** ((Φ_{ref} = 0.94)[4] as referenced. The absorption at the excitation wavelengths were below 0.1 to avoid inner filter effect, concentrations were [**L**] = [Ln(III)]; [**L4**] = 10 µM, [**L5**] = 7 µM, [**L6**] = 15 µM, [**L7**] = 2 µM, [**L9**] = 1.67 µM. Quantum yields were calculated according to Eq. S1, with Φ_s the quantum yield of the sample, Φ_{ref} the quantum yield of the reference, *I* the integrated corrected emission intensity of the sample (s) and of the reference (ref), f_A the absorption factor of the sample (s) and of the reference (ref) at the excitation wavelength and n the refractive indexes of the sample (s) and of the reference (ref). The concentration of the references were adjusted to obtain an absorbance matching with the maxima of the chromophore in a mixture of **L**:Ln(OTf)$_3$. For the experiments with serial dilutions the ratio of the L and Ln(OTf)$_3$ were kept constant. The excitation wavelength where the absorption factors of the samples and of the reference were the same was chosen (i.e. where the absorptions are identical). The corrected emission spectra of the sample and reference standard were then measured under the same conditions over the spectral range as well as blank samples containing only the solvent. The appropriate blanks were subtracted from their respective spectra and the antenna fluorescence was separated by fitting the section of the antenna emission exponentially overlapping the lanthanide emission (**L4**). The quantum yields were calculated according to Equation S1. The given relative error on the quantum yields ($\delta\Phi = \Delta\Phi/\Phi$, where $\Delta\Phi$ is the absolute error) take into account the accuracy of the spectrometer and of the integration procedure [$\delta(I_s/I_{ref}) < 2\%$], an error of 0.59 ±0.01 on the quantum yield of the reference QS [$\delta(\Phi_{ref}) < 2\%$], an error on the ratio of the

absorption factors [δ(f_{Aref}/f_{As}) < 5%, relative to the fixed absorption factor of the reference QS] and an error on the ratio of the squared refractive indexes [δ(n_s^2/n_{ref}^2) < 1%, < 0.25% around 1.333 for H_2O and 1.430 for DMF on each individual refractive index], which sums to a total estimated relative error that should be δΦ_s < 10%. A limit value of 10% is thus chosen.

$$\Phi_S = \frac{I_S}{I_{ref}} \cdot \frac{f_{Aref}}{f_{As}} \cdot \frac{(n_S)^2}{(n_{ref})^2} \cdot \Phi_{ref} \qquad \text{(S1)}$$

The quantum yield of **L7** was also determined as follows. Serial dilutions of solutions of C153 and **L7** were prepared keeping A < 0.1. The fluorescence emission was recorded as described above, and I *vs* A was plotted (Figures S50–S55). The concentrations of the reference were not adjusted to obtain an absorbance matching with the maxima of the chromophore in a mixture of **L**:Ln(OTf)$_3$. In these experiments ratio of **L7** and Ln(OTf)$_3$ were kept constant.

Fourier transform infrared spectroscopy (FTIR). Measurements were done on a Perkin Elmer Spectrum One instrument. Spectra were recorded on dry samples by making a pellet using KBr with the ligand or complex (100:1). Blank was recorded with only a KBr pellet.

Gas chromatography with mass spectrometry (GC-MS). Photoreactions were monitored by GC-MS (Agilent 7890A GC and 5975 MSD system). Samples were injected using split injection (1 μL injection volume; split ratio: 100:1; 250 °C inlet temperature; flow rate: 120 mL/min). The temperature rate was set to 20 °C/min resulting in a 12.5 min total run time. He was used as a carrier gas at a flow rate of 1.2 mL/min. The column used was an Agilent 19091S-433: 325 °C: 30 m x 250 μm x 0.25 μm (front SS-inlet: He; out: vacuum). Mass spectrometer: Source temperature: 230 °C, Quad-temperature 150 °C.

Electrochemistry. Cyclic voltammograms (CV) were obtained at room temperature (~20 °C) using an AUTOLAB PGSTAT 100 potentiostat, or an AUTOLAB PGSTAT 204N potentiostat. The setup was equipped with a 3 mm glassy carbon (GC) working electrode, a Pt wire auxiliary electrode, and an Ag/AgCl as a reference electrode. Measurements were done in anhydrous DMF and MeCN with NBu_4PF_6 (0.1 M) as the supporting electrolyte. The voltammograms were recorded by scanning first toward more negative potential values (reduction). A step-potential of –0.9 mV was used for 100 mV/s scan rates.

A solution of NBu_4PF_6 (0.1 M) in DMF/MeCN (2 mL) was added to the electrochemical cell. The working electrode was polished with 0.05 μm alumina on a polishing pad, washed with water and ethanol and dried. This was repeated before each new sample. The three electrodes (GC working electrode, platinum wire auxiliary electrode, and Ag/AgCl reference electrode) were inserted into the cell setup followed by argon purging for 10 mins and a background scan was recorded with a scan rate of 100 mV/s, and two sweeps. The complexes were added to the solution (2-5 mM), purged again for 10 mins and the sample was recorded.

Spectro-electrochemistry was performed in an argon-filled glovebox with a solution of Eu(OTf)$_3$ and acetic acid (1.33 mM) in acetonitrile using NBu_4PF_6 (0.1 M) as the electrolyte. The three electrodes (carbon (mesh) as a working electrode and counter electrode, and Ag/$AgNO_3$ as the reference electrode) were used. A potential of –0.35 V (vs Ag/$AgNO_3$) was applied for 30 mins and UV was recorded every 30 s during the measurement.

EPR Spectroscopy. EPR measurements at room temperature was performed using a Bruker EMX Micro spectrometer, equipped with an ER 4119HS resonator. EPR samples were prepared in a 1 mm capillary. EPR parameters: microwave frequency, 9.86 GHz; modulation frequency, 100 kHz. EPR measurements at 10 K were performed using a Bruker ESR-500 spectrometer, equipped with an ER 4122SHQ resonator, an ESR900 cryostat, and an Oxford ITC503 temperature controller. EPR parameters: microwave frequency, 9.38 GHz; modulation frequency, 100 kHz. All the parameters are constant unless indicated otherwise.

Photoreaction setup. All reactions were performed in microwave vials equipped with a stirring bar, in a dry glovebox [O_2 (<0.5 ppm), H_2O (<0.5 ppm)] with an Ar atmosphere. The vials were charged with **1a** (1.0 equiv., 0.087 mmol), **L** (0.1 equiv., 0.0087 mmol), Eu(OTf)$_3$ (0.1 equiv., 0.0087 mmol), DIPEA (5.0 equiv., 0.435 mmol, 76 μl), LiCl (5.0 equiv., 0.435 mmol), Zn (if applicable, 1.0 equiv., 0.087

mmol), and DMF (1 mL), or with **1a** (1.0 equiv., 0.087 mmol), **L** (0.1 equiv., 0.0087 mmol), $Eu(OTf)_3$ (0.1 equiv., 0.0087 mmol), DIPEA (5.0 equiv., 0.435 mmol), LiCl (5.0 equiv., 0.435 mmol), and DMF:water (4:1, 1 mL), and were then sealed with an electric black tape. 40 W blue LED lamp (Kessil A160WE Tuna Blue, λ_{max} = ~ 450 nm, set highest blue color and intensity) was used for irradiation. Reactions were stirred at 600–1000 rpm. GCMS yield determined using a calibration curve prepared from integrated peak areas of **1b** (0.18–1.25 mM), and **1c** (0.04–0.7 mM) solutions. For the full emission spectrum of the A160WE Tuna Blue light source see reference.[5]

Irradiation experiments. All reactions were performed in quartz cuvettes loaded with $Ln(OTf)_3$ (0.3 mM), **L** (0.3 mM) and DIPEA (5.7 mM) and DMF (3 mL) in a glovebox [O_2 (<0.5 ppm). The vials were sealed with an electric black tape. 40 W blue LED lamp (Kessil A160WE Tuna Blue, λ_{max} = ~ 450 nm, set highest blue color and intensity) was used for irradiation for the indicated length of time. The absorption and emission were recorded as described above.

$Eu(OTf)_2$ preparation. Method 1:[6] To the stirred solution of EuI_2 (4 mg, 1.0 equiv.) in DMF (1 mL), AgOTf (4.8 mg, 2.0 equiv.) was added. The clear dark yellow solution turned immediately into a sandy mixture. Stirring was continued for 20–30 mins. The mixture was filtered using a syringe filter to get pale yellowish solution. 250 µL of this solution was added to DMF to yield a 3 mL solution in a cuvette. DIPEA (260 µL, 0.5 mM) was added dropwise to acetic acid (85 µL, 0.5 mM) in DMF, and the resulting solution was added in the $Eu(OTf)_2$-solution in DMF for spectroscopy.

Method 2:[7] To a stirred solution of $Eu(OTf)_3$ (5 mg, 1 equiv.) in DMF (1 mL), Zn (22 mg, 40 equiv.) was added. The mixture was stirred for 2–3 hours. This mixture was filtered using a syringe filter to get a pale solution. A 250 µL sample of this solution was added to DMF to afford a total volume of 3 mL in a cuvette.

X-ray crystallography. Single crystals of **L4**-Eu(III) were obtained by slow evaporation of Methanol layered with diethylether. A suitable crystal was selected and mounted using Fomblin oil on a fiber-loop on a XtaLAB Synergy, Single source (CuKα) diffractometer equipped with a HyPix detector. The crystal was kept at 100.00(10) K during data collection. Using Olex2,[8] the structure was solved with the SHELXT structure solution program using Intrinsic Phasing and refined with the SHELXL[9] refinement package using Least Squares minimization.

2. Photophysical characterization

Absorption and emission, and excitation spectra of L and LnL

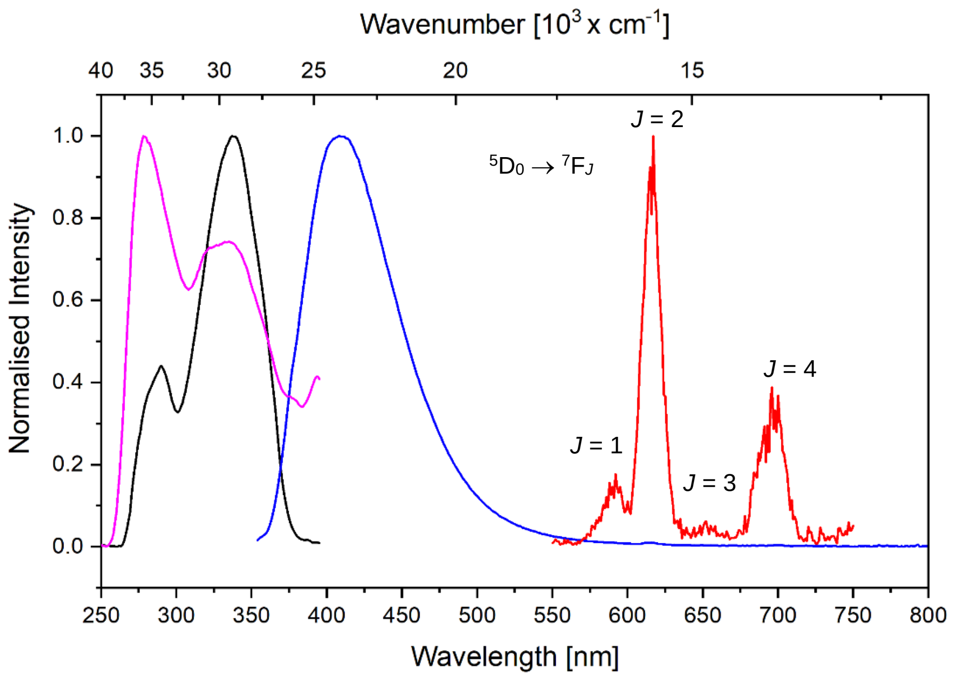

Figure S1. Excitation spectra of ligand fluorescence (black) and Eu(III) emission (magenta) (λ_{em} = 412 nm and 616 nm, respectively), and steady-state and time-resolved emission spectra (blue and red, respectively, λ_{ex} = 339 nm) of **EuL1**, [**EuL1**] = 16.7 µM in DMF).

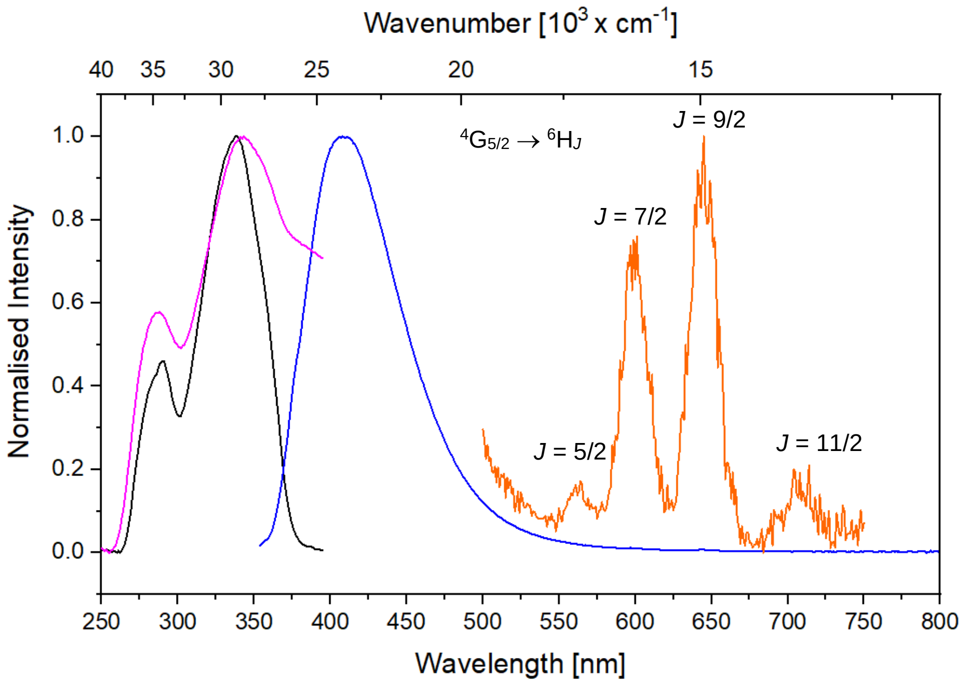

Figure S2. Excitation spectra of ligand fluorescence (black) and Sm(III) emission (magenta) (λ_{em} = 412 nm and 601 nm, respectively), and steady-state and time-resolved emission spectra of **SmL1** at r.t. (blue and orange, respectively, λ_{ex} = 339 nm, [**SmL1**] = 13.6 µM in DMF).

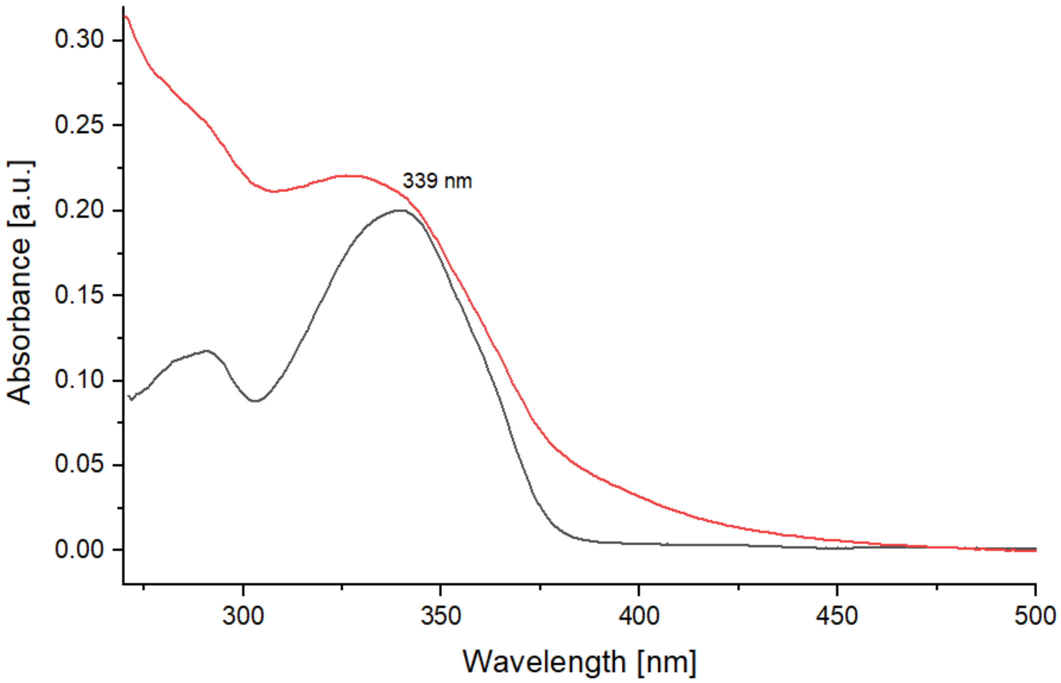

Figure S3. UV-Vis absorption spectra of **EuL2** in DMF (black) and in DMF:H_2O (4:1, red); [**EuL2**] = 58 µM.

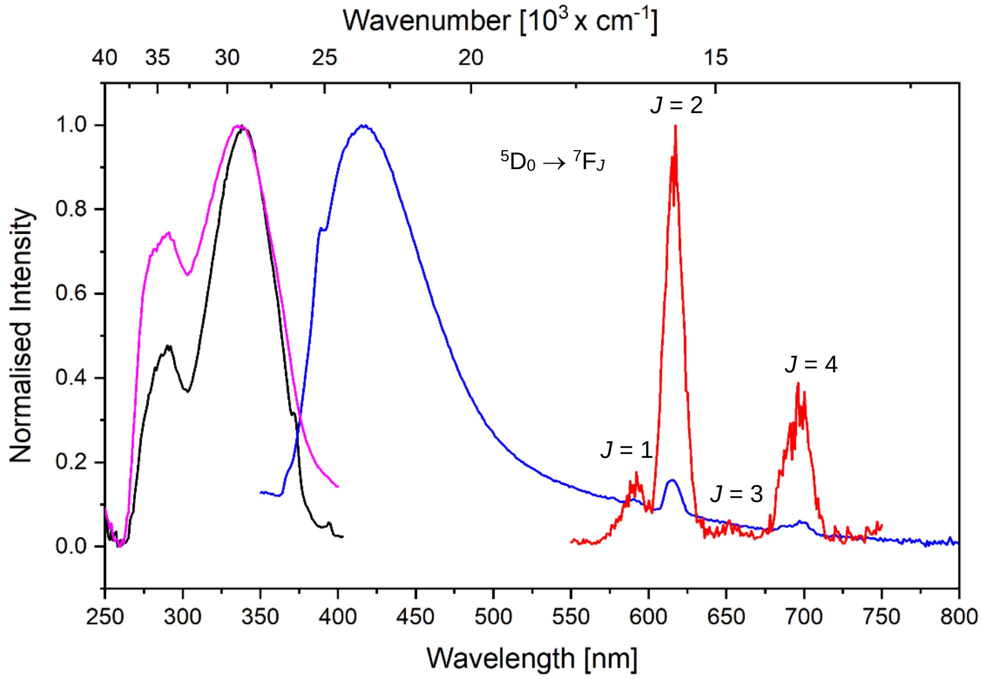

Figure S4. Excitation spectra of ligand fluorescence (black) and Eu(III) emission (magenta) (λ_{em} = 418 nm and 616 nm, respectively), and steady-state and time-resolved emission spectra of **EuL2** ([**EuL2**] = 58 µM in DMF) at r.t. (blue and red, respectively, λ_{ex} = 348 nm).

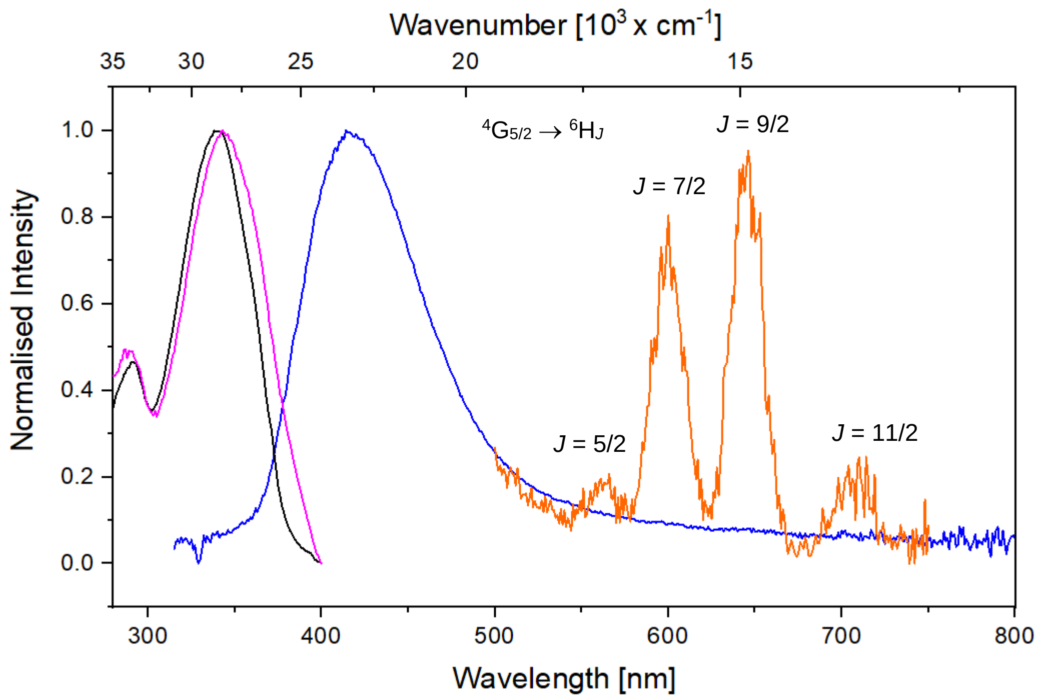

Figure S5. Excitation spectra of ligand fluorescence (black) and Sm(III) emission (magenta) (λ_{em} = 417 nm and 601 nm, respectively) and steady-state (blue) and time-resolved (orange) emission spectra (λ_{ex} = 328 nm and 339 nm, respectively) of **SmL2** ([**SmL2**] = 17 µM in DMF).

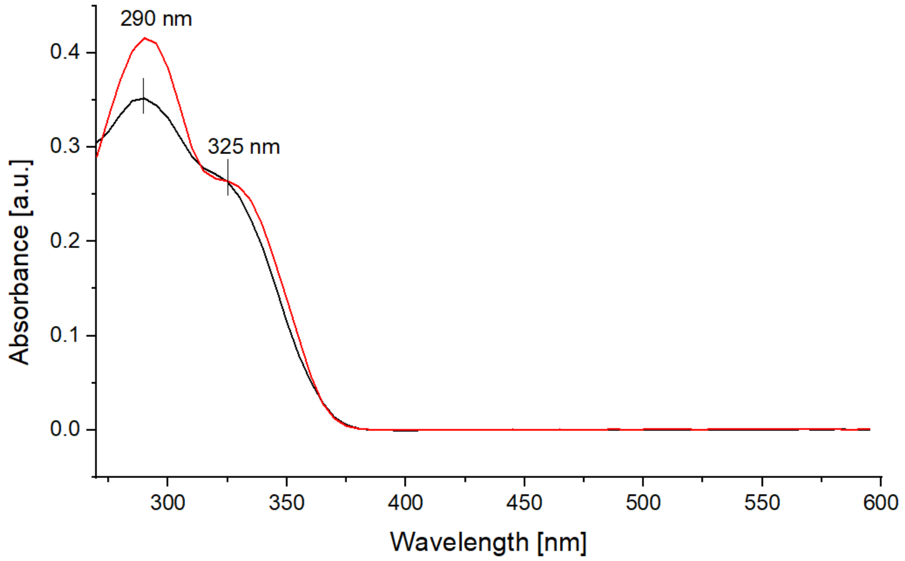

Figure S6. UV-Vis absorption spectra of **L4** (black, 17 µM) and **L4** + Eu(OTf)$_3$ (equimolar amounts, red, 17 µM) in DMF.

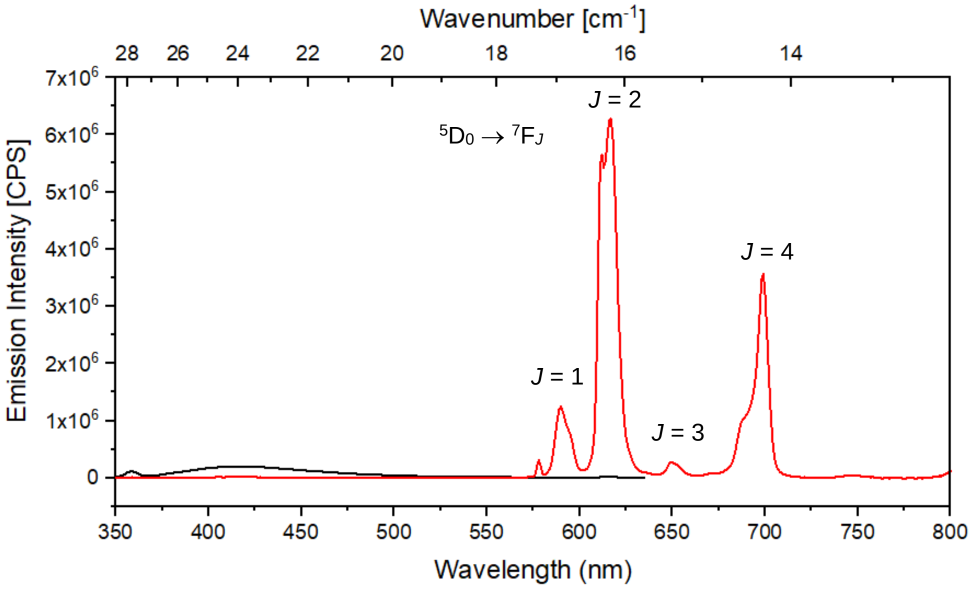

Figure S7. Steady-state emission spectra of **L4** (λ_{ex} = 325 nm, black, 17 µM) and **L4** + $Eu(OTf)_3$ (equimolar amounts, λ_{ex} = 325 nm, red, 17 µM) in DMF.

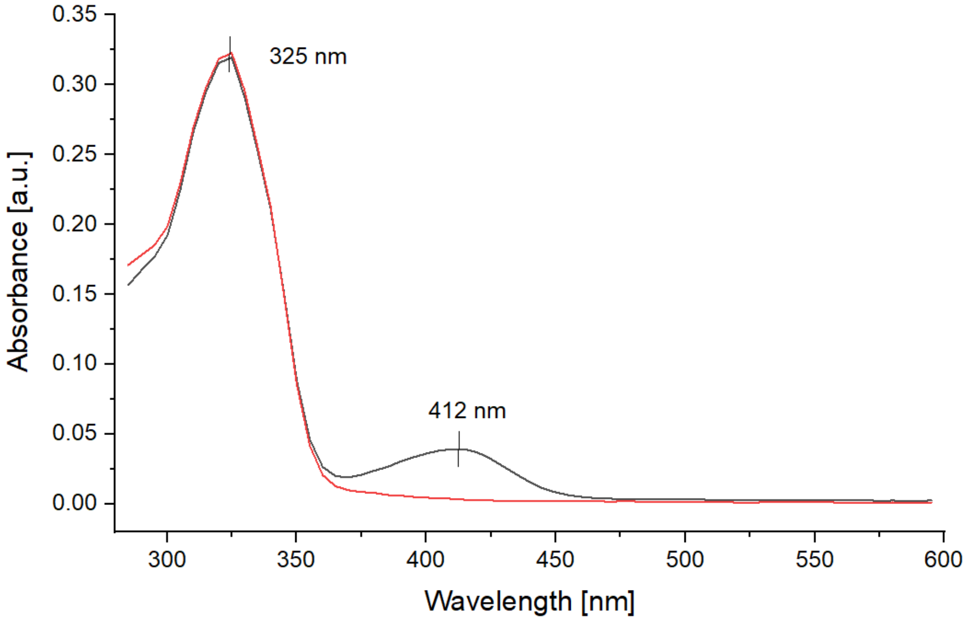

Figure S8. UV-Vis absorption spectra of **L5** (black, 17 µM) and **L5** + $Eu(OTf)_3$ (equimolar amounts, red, 17 µM) in DMF.

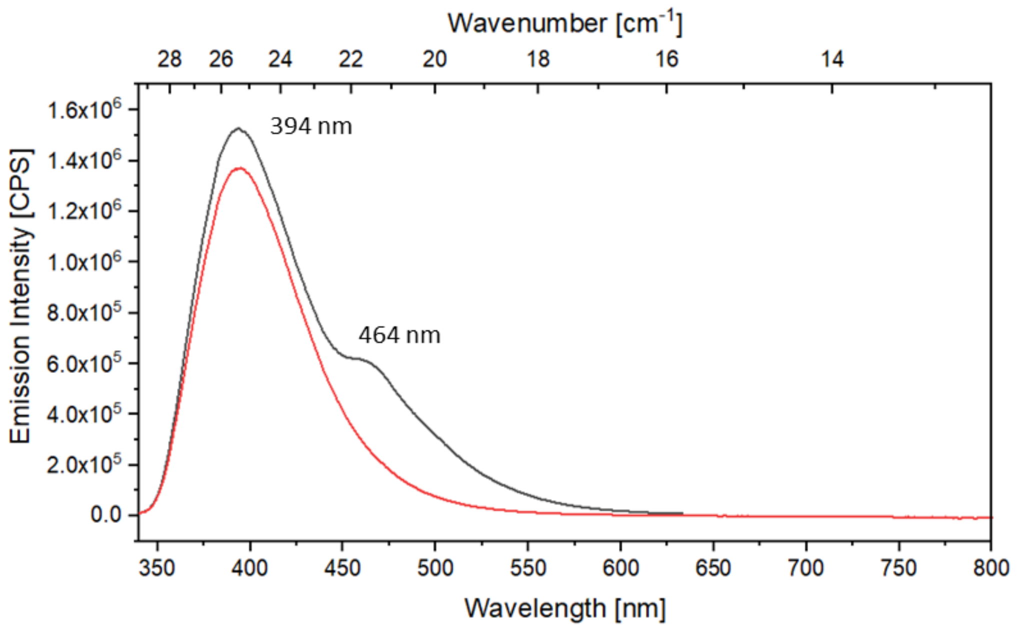

Figure S9. Steady-state emission spectra of **L5** (black, λ_{ex} = 325 nm, 17 µM) and **L5** + $Eu(OTf)_3$ (equimolar amounts, red, λ_{ex} = 325 nm, 17 µM) in DMF.

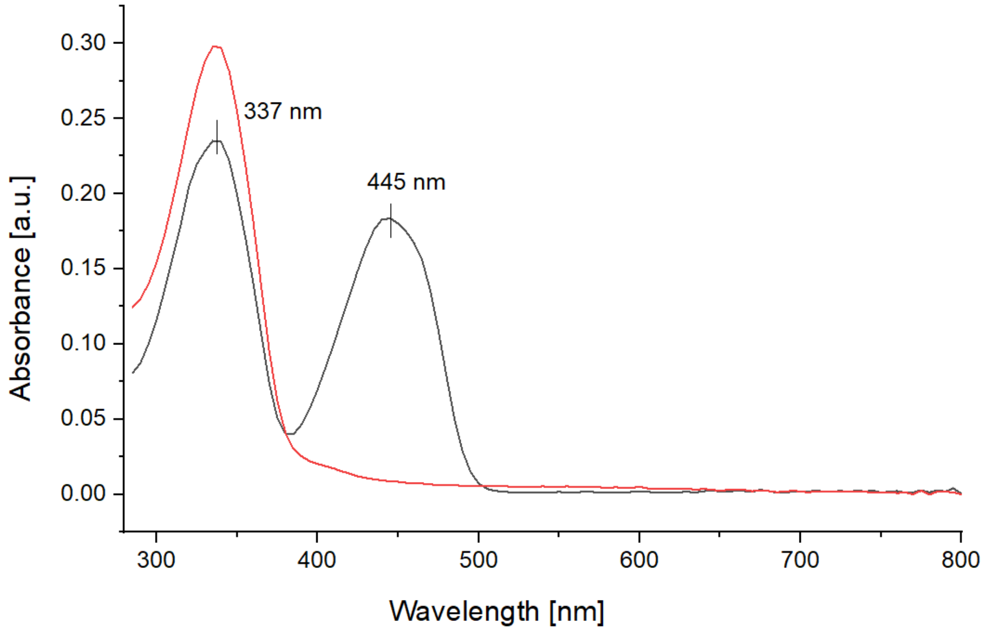

Figure S10. UV-Vis absorption spectra of **L6** (black, 17 µM) and **L6** + $Eu(OTf)_3$ (equimolar amounts, red, 17 µM) in DMF.

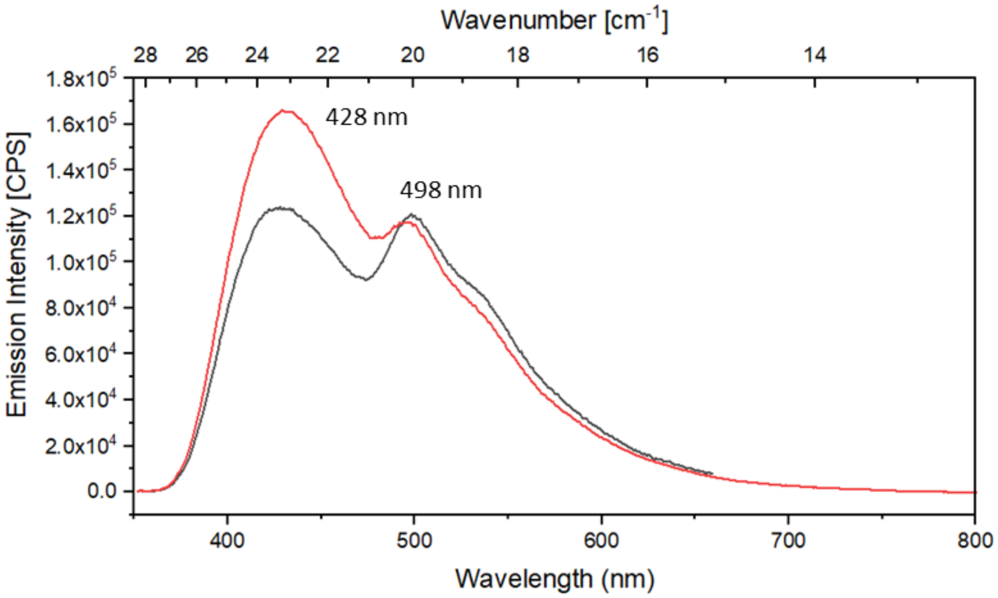

Figure S11. Steady-state emission spectra of **L6** (black, λ_{ex} = 337 nm, 17 µM) and **L6** + $Eu(OTf)_3$ (equimolar amounts, red, λ_{ex} = 337 nm, 17 µM) in DMF.

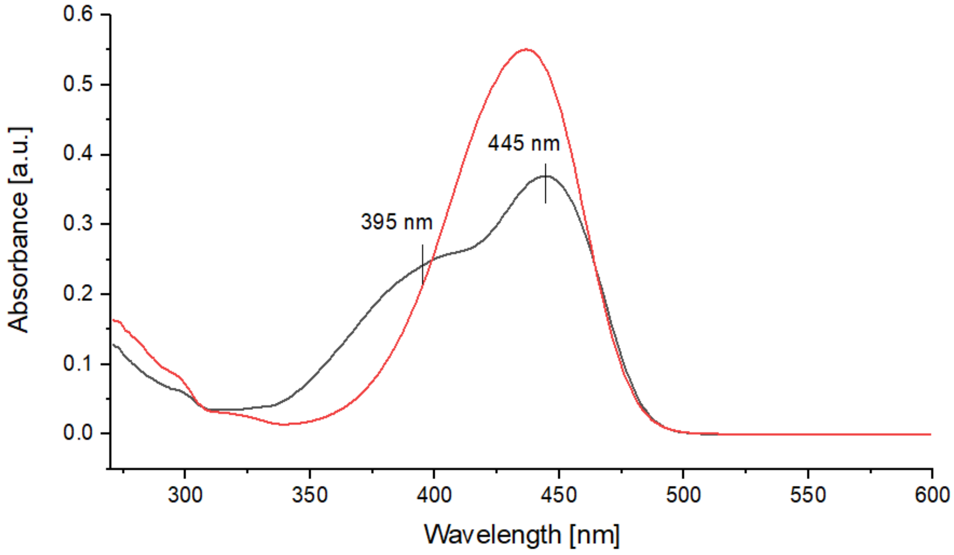

Figure S12. UV-Vis absorption spectra of **L7** (black, 17 µM) and **L7** + $Eu(OTf)_3$ (equimolar amounts, red, 17 µM) in DMF.

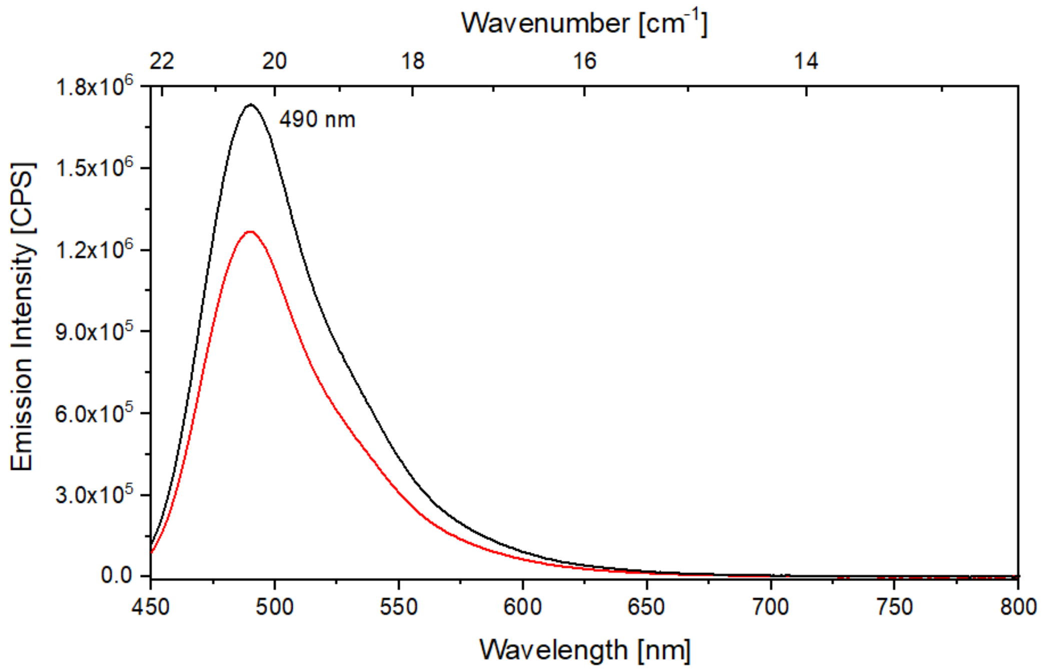

Figure S13. Steady-state emission spectra of **L7** (black, λ_{ex} = 440 nm, 17 µM) and **L7** + $Eu(OTf)_3$ (equimolar amounts, red, λ_{ex} = 440 nm, 17 µM) in DMF.

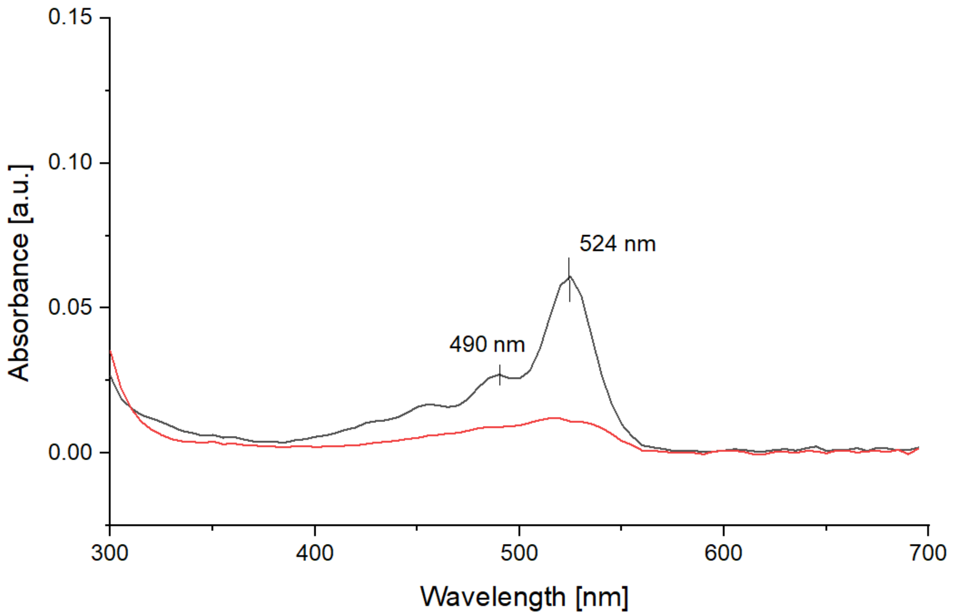

Figure S14. UV-Vis absorption spectra of **L8** (black, 17 µM) and **L8** + $Eu(OTf)_3$ (equimolar amounts, red, 17 µM) in DMF.

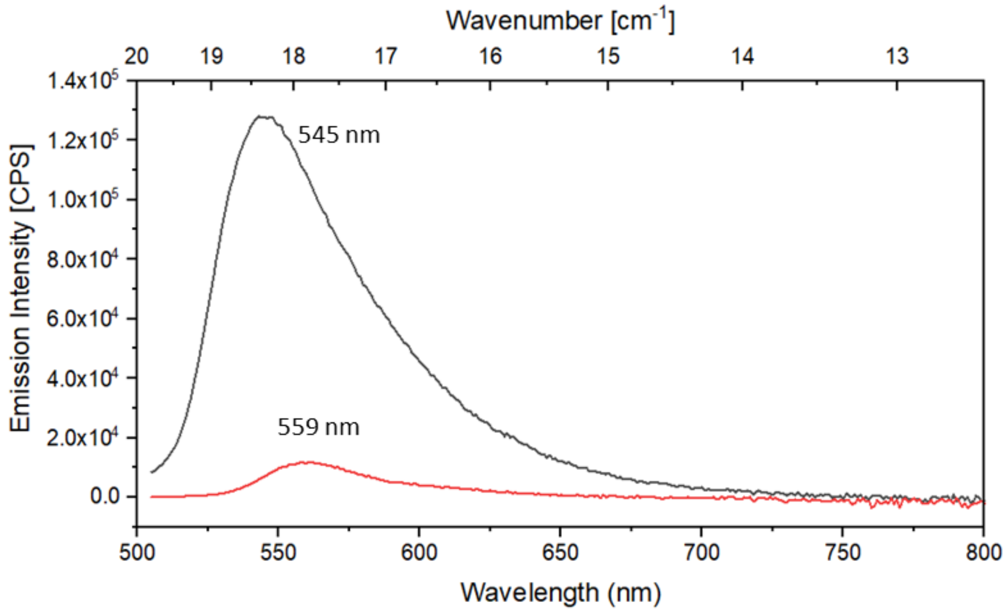

Figure S15. Steady-state emission spectra of **L8** (black, λ_{ex} = 490 nm, 17 µM) and **L8** + $Eu(OTf)_3$ (equimolar amounts, red, λ_{ex} = 490 nm, 17 µM) in DMF.

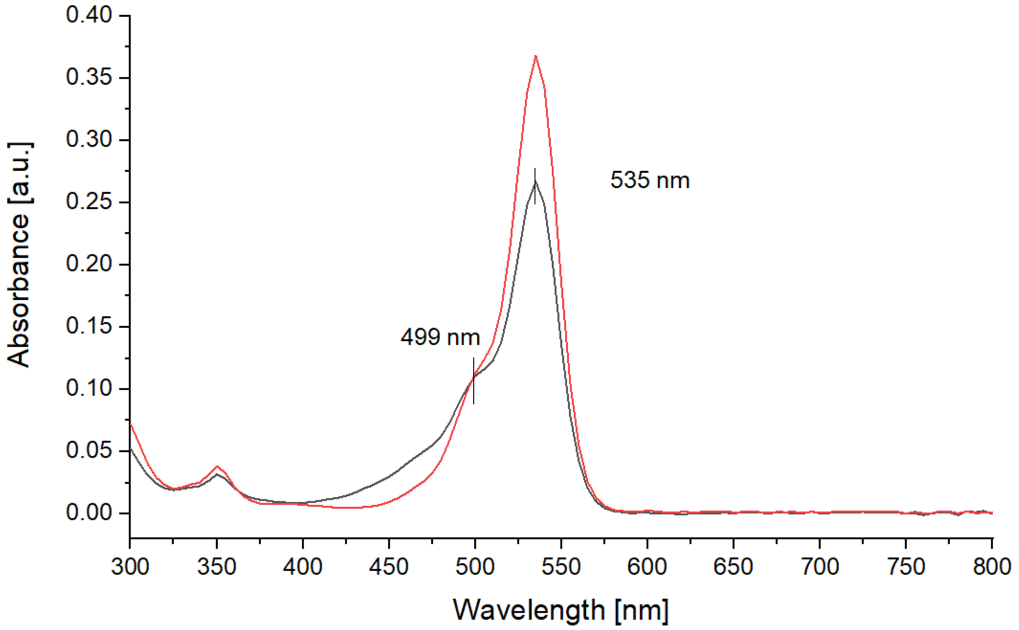

Figure S16. UV-Vis absorption spectra of **L9** (black, 3.4 µM) and **L9** + $Eu(OTf)_3$ (equimolar amounts, red, 3.4 µM) in DMF.

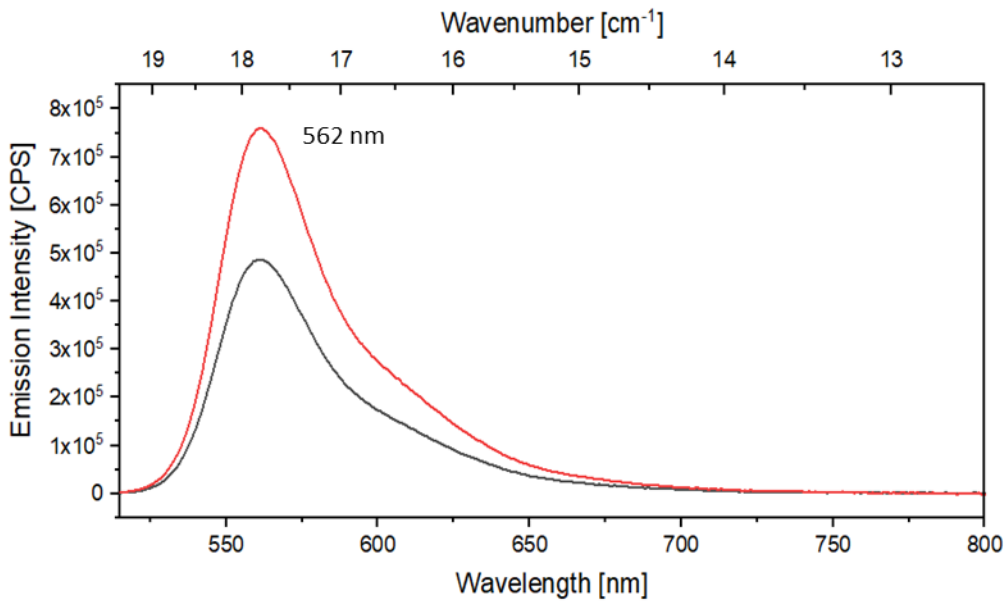

Figure S17. Steady-state emission spectra of **L9** (black, λ_{ex} = 499 nm, 3.4 µM) and **L9** + Eu(OTf)$_3$ (equimolar amounts, red, λ_{ex} = 499 nm, 3.4 µM) in DMF.

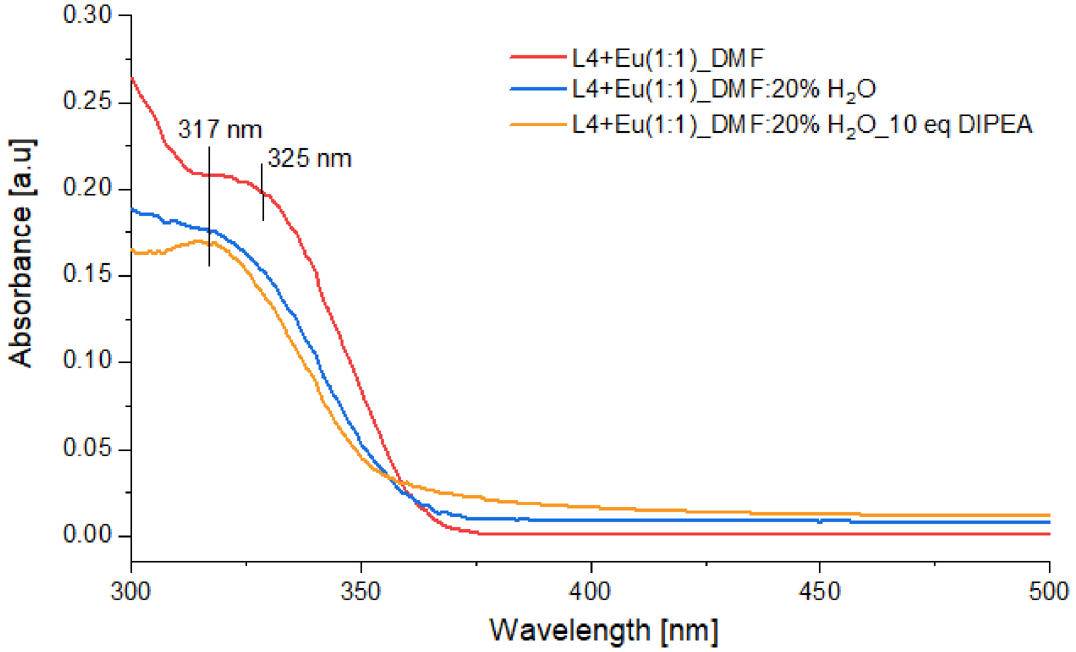

Figure S18. Absorption spectra of **L4** ([**L4**] = 16.5 µM) in the presence of Eu(OTf)$_3$ under various conditions.

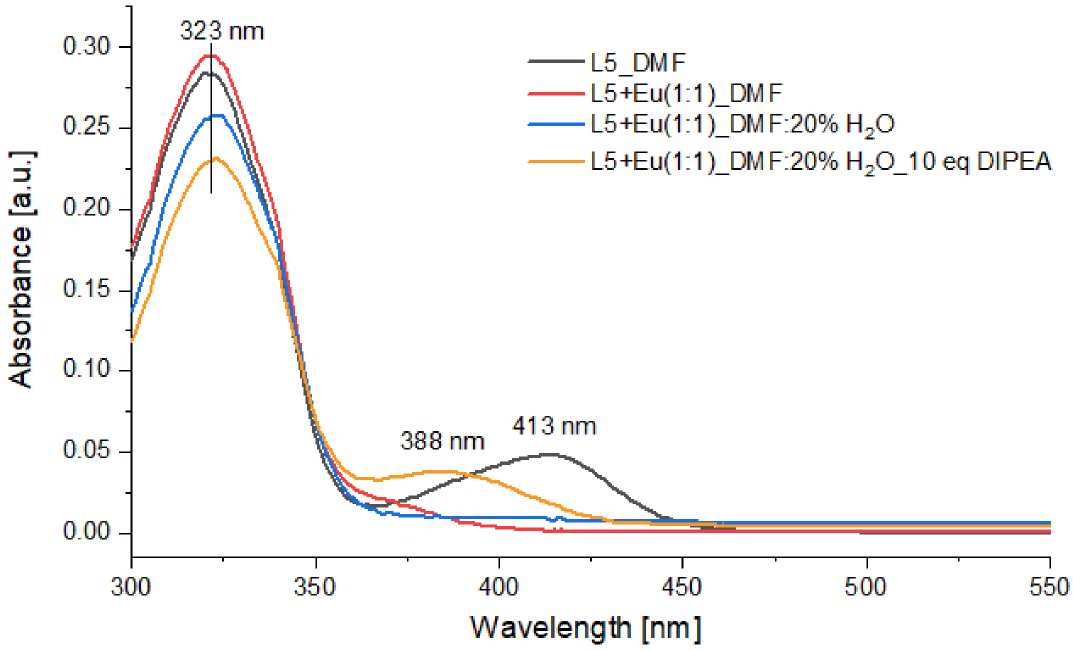

Figure S19. Absorption spectra of **L5** (16.5 µM) in the presence of $Eu(OTf)_3$, $Eu(OTf)_3$ + 20% H_2O, and $Eu(OTf)_3$ + DIPEA + 20% H_2O (10 equiv.).

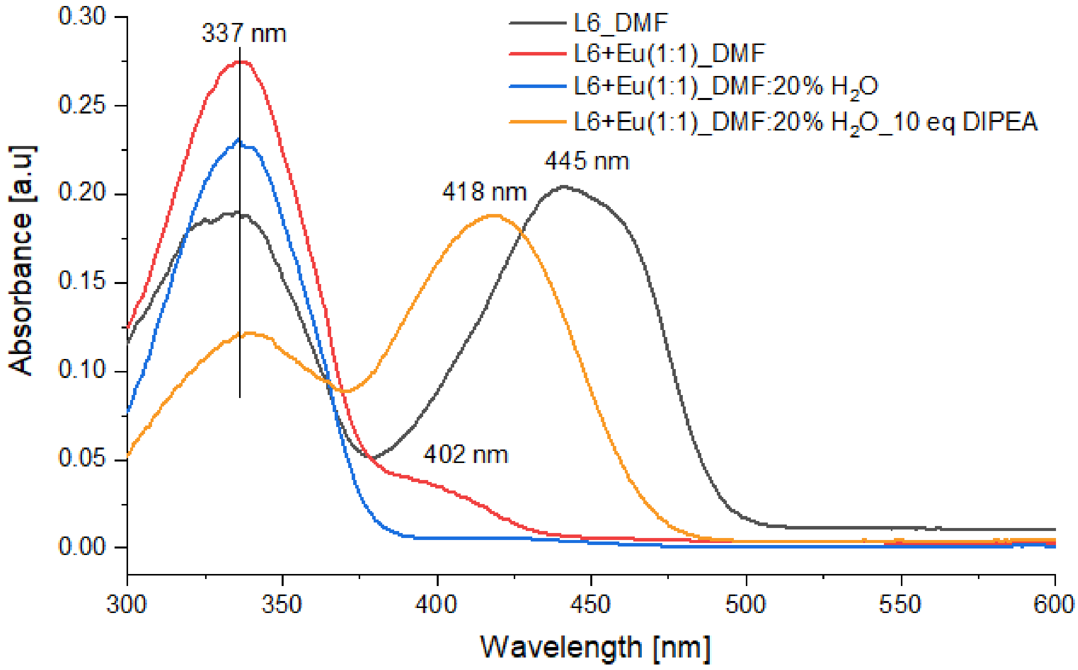

Figure S20. Absorption spectra of **L6** (16.5 µM) in the presence of $Eu(OTf)_3$, $Eu(OTf)_3$ + 20% H_2O, and $Eu(OTf)_3$ + DIPEA + 20% H_2O (10 equiv.).

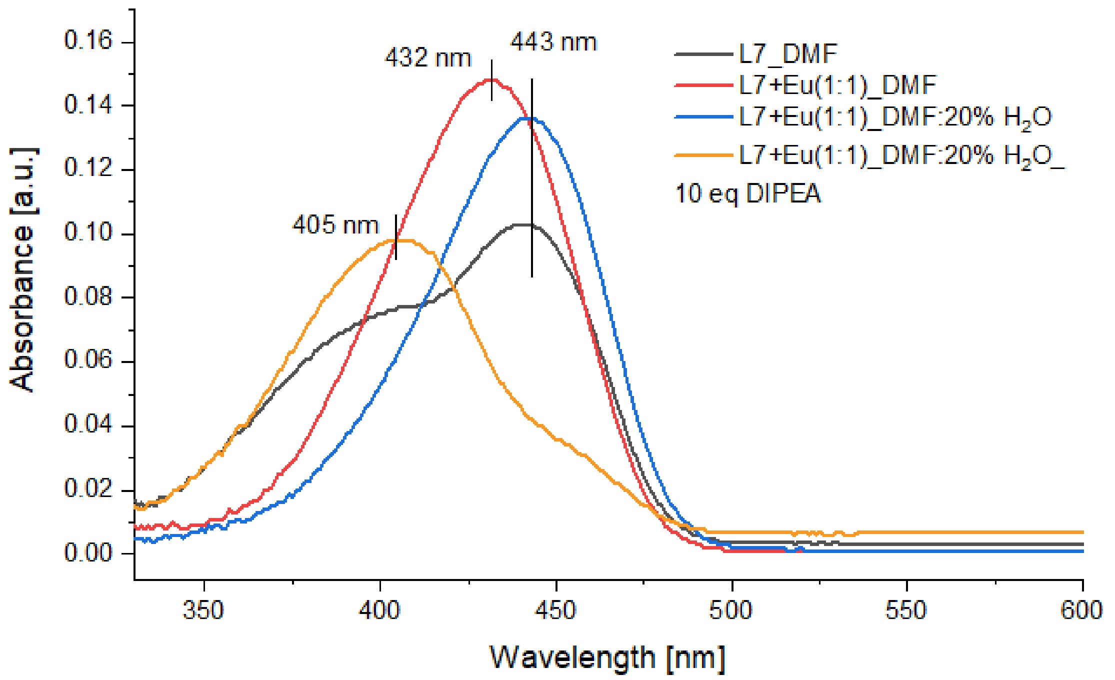

Figure S21. Absorption spectra of **L7** (3.4 µM) in the presence of $Eu(OTf)_3$, $Eu(OTf)_3$ and 20% H_2O, and $Eu(OTf)_3$ and 20% H_2O and DIPEA (10 equiv.).

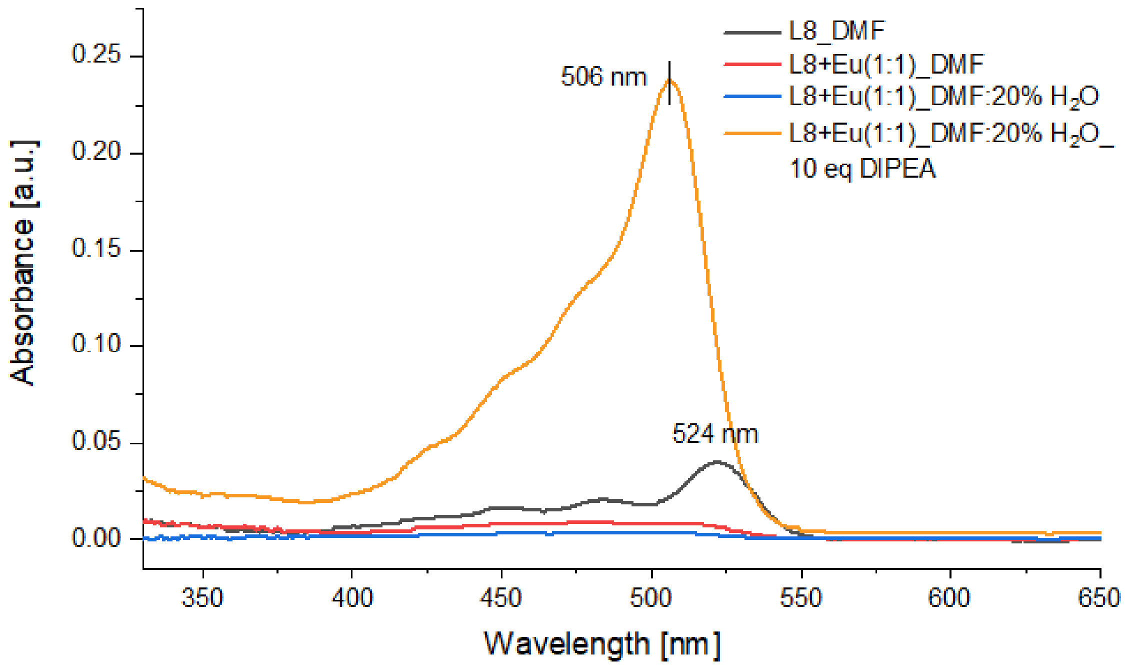

Figure S22. Absorption spectra of **L8** (16.5 µM) in the presence of $Eu(OTf)_3$, $Eu(OTf)_3$ and 20% H_2O, and $Eu(OTf)_3$ and 20% H_2O and DIPEA (10 equiv.).

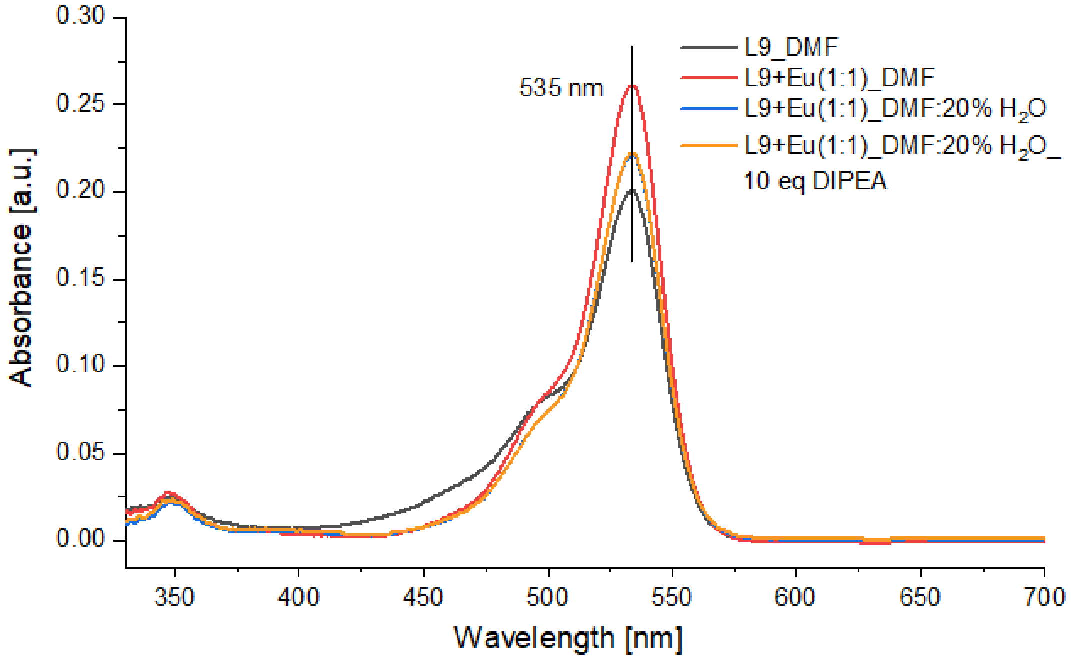

Figure S23. Absorption spectra of **L9** (3.4 µM) in the presence of $Eu(OTf)_3$, $Eu(OTf)_3$ and 20% H_2O, and $Eu(OTf)_3$ and 20% H_2O and DIPEA (10 equiv.).

Low-temperature spectra of GdL and L

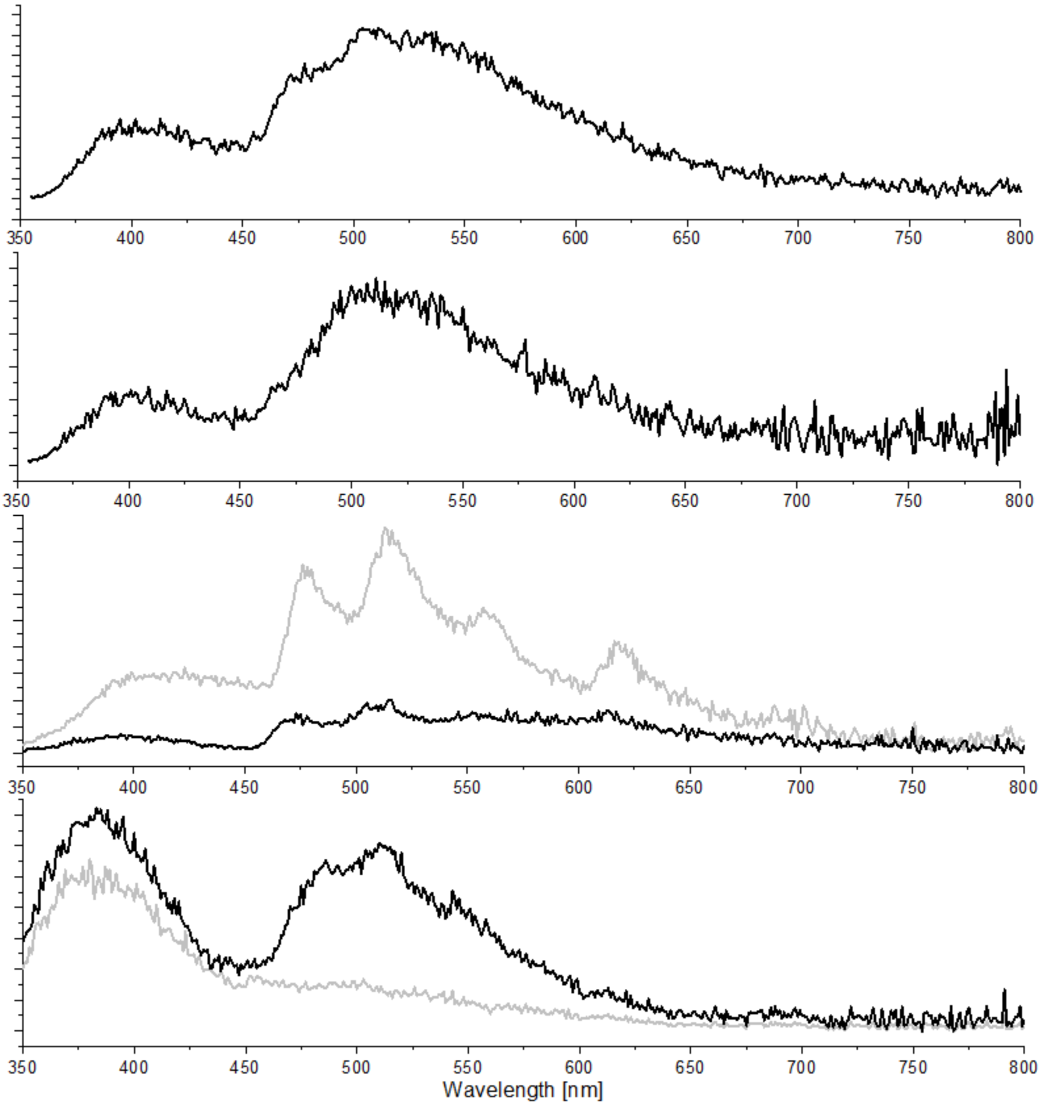

Figure S24. Time resolved emission spectra (50 µs delay) of **GdL1** (1st from top, λ_{ex} = 340 nm), **GdL2** (2nd from top, λ_{ex} = 340 nm), **L4** (grey) and **L4** + Gd(OTf)$_3$ (black, 3rd from top, λ_{ex} = 325 nm), **L5** (grey) and **L5** + Gd(OTf)$_3$ (black, 4th from top, λ_{ex} = 325 nm) at 77 K in DMF.

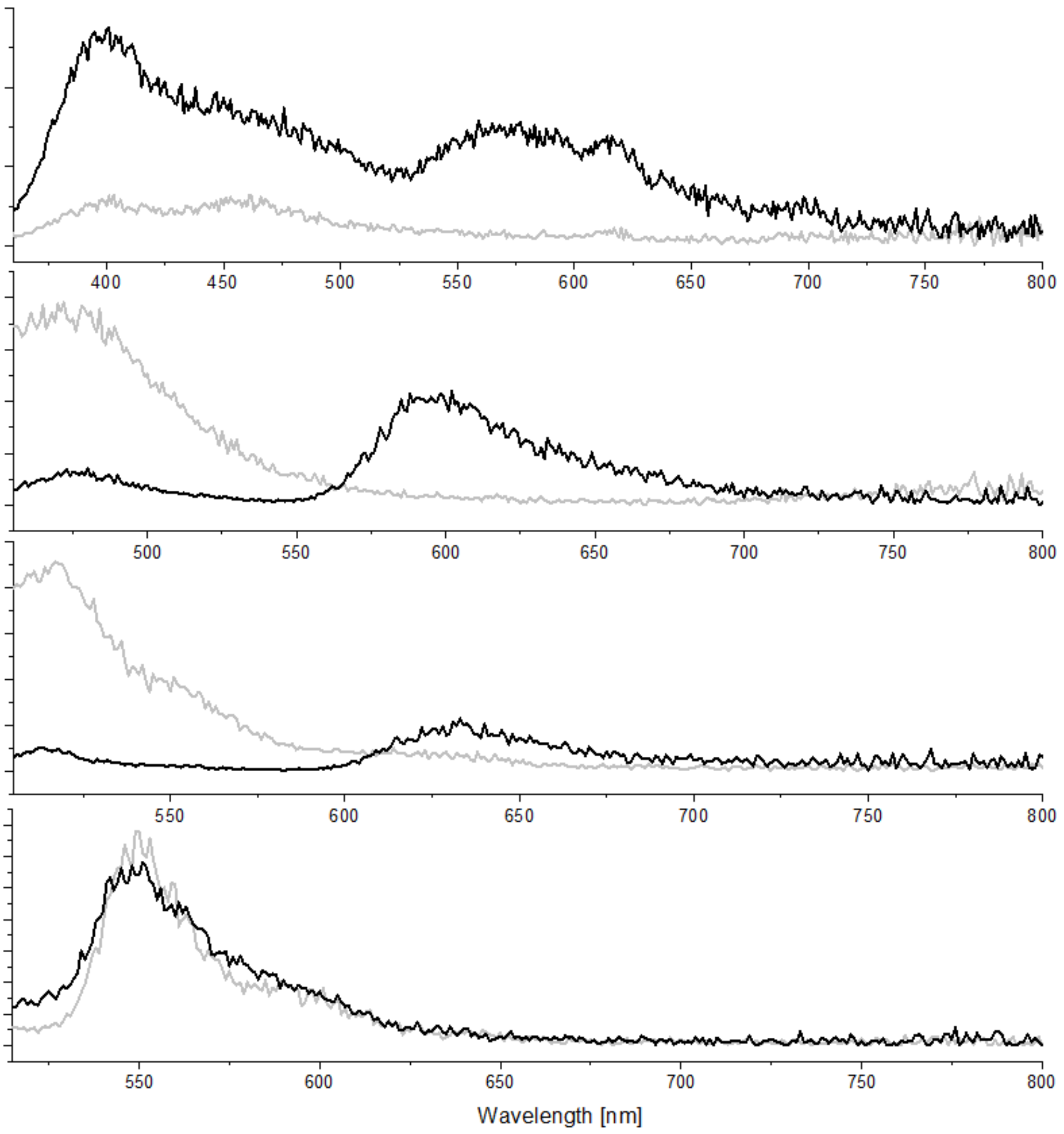

Figure S25. Time resolved emission spectra (50 µs delay) of **L6**–**L9** (grey) and of **L6**–**L9** + $Gd(OTf)_3$ (black) at 77 K in DMF, (from top to bottom: **L6**, λ_{ex} = 337 nm; **L7**, λ_{ex} = 440 nm; **L8**, λ_{ex} = 489 nm; **L9,** λ_{ex} = 499 nm).

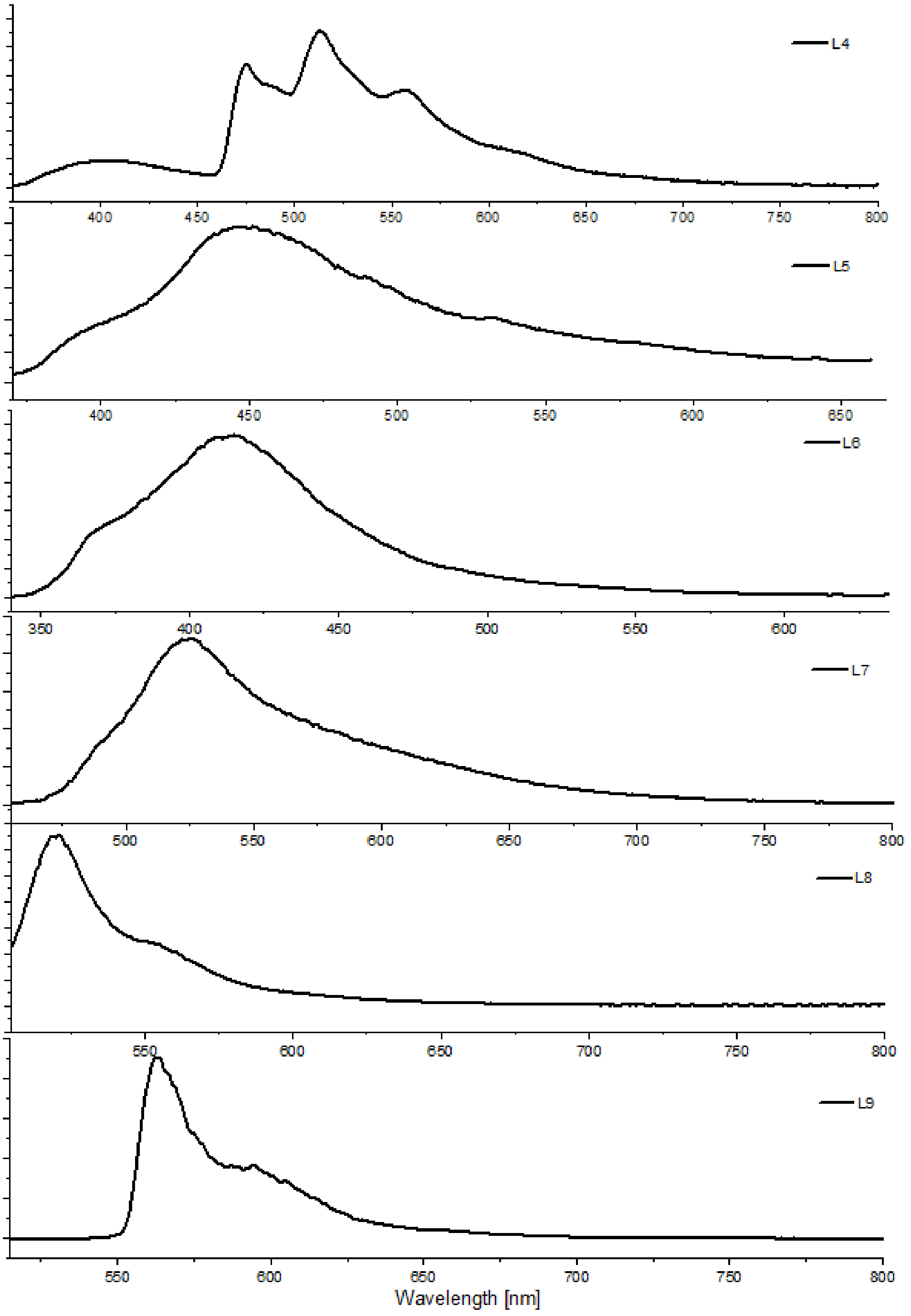

Figure S26. Steady state emission spectra of **L4**–**L9** at 77 K in DMF, (**L4** and **L5** = λ_{ex} = 325 nm; **L6**, λ_{ex} = 337 nm; **L7**, λ_{ex} = 440 nm; **L8**, λ_{ex} = 489 nm; **L9,** λ_{ex} = 499 nm).

Quenching experiments

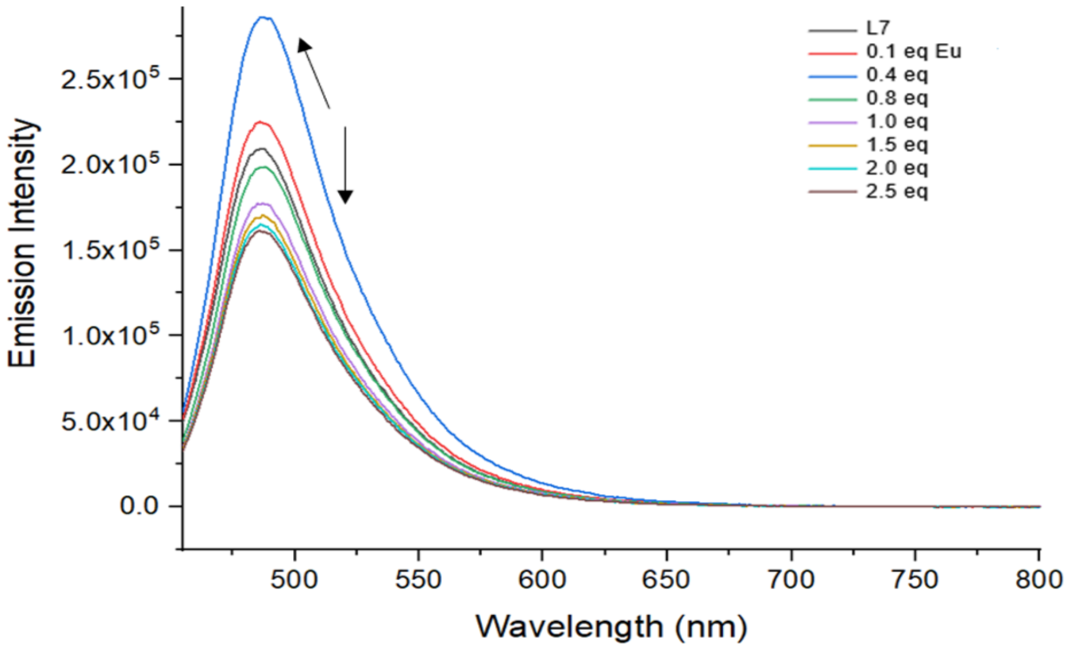

Figure S27. Steady-state fluorescence spectra of **L7** in the presence of increasing amounts of $Eu(OTf)_3$ in DMF; λ_{ex} = 440 nm, [**L7**] = 3.4 µM.

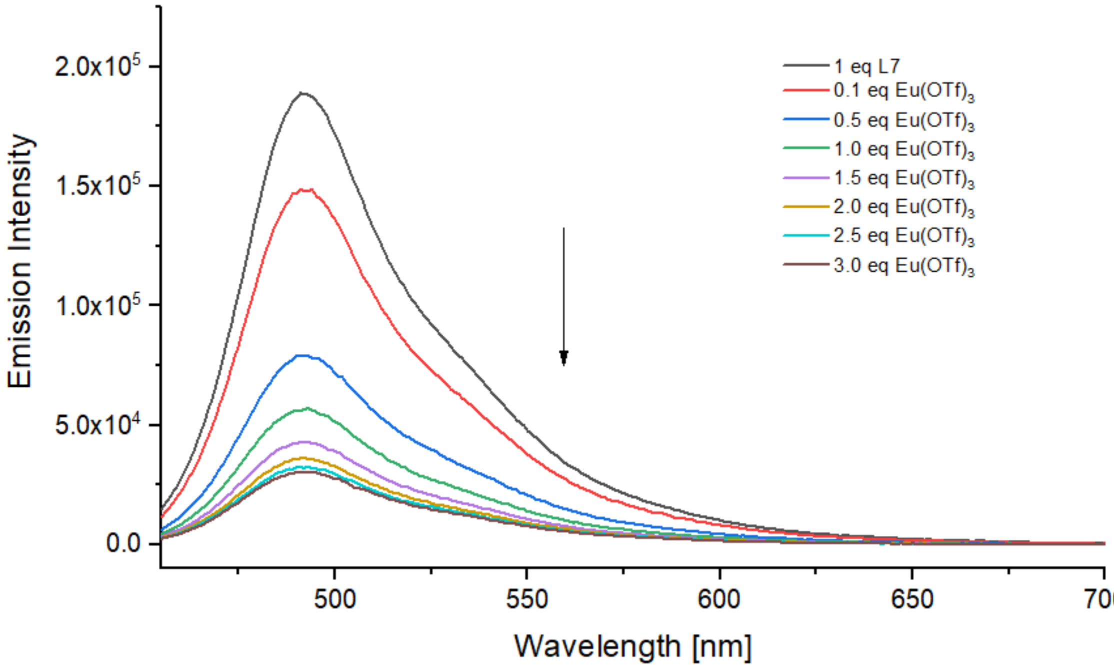

Figure S28. Steady-state fluorescence spectra of **L7** in the presence of increasing amounts of $Eu(OTf)_3$ in MeCN; λ_{ex} = 440 nm, [**L7**] = 3.4 µM.

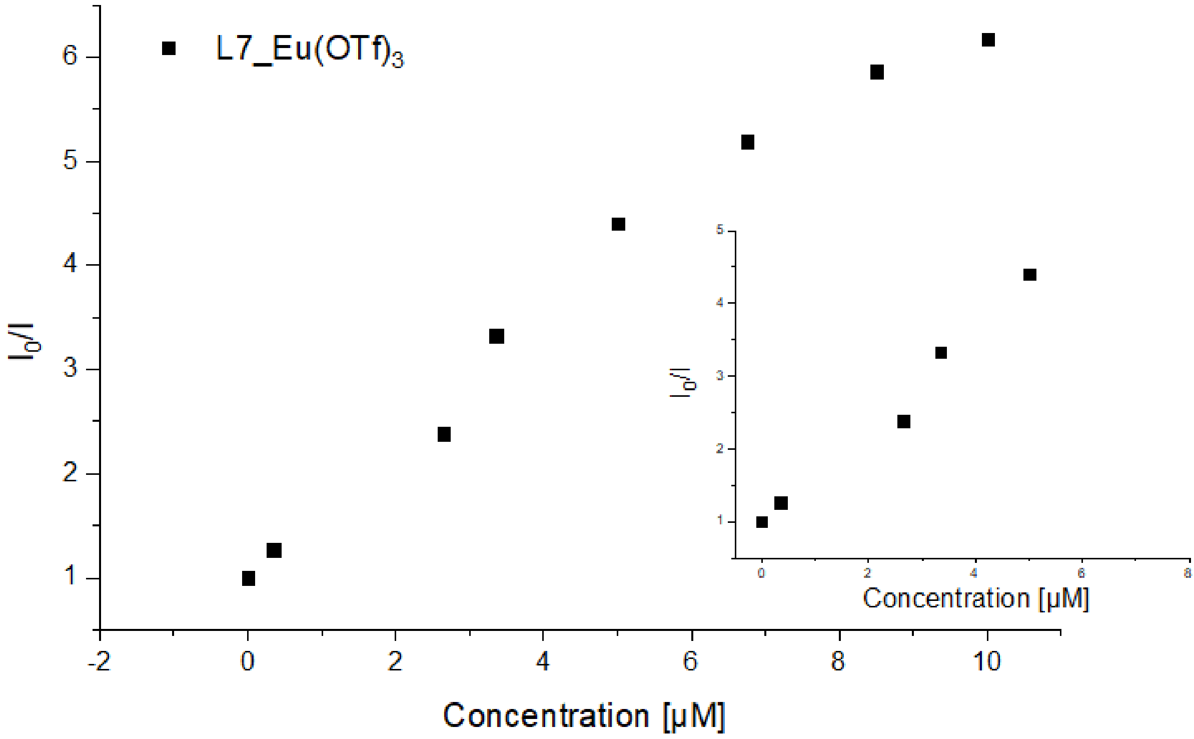

Figure S29 Quenching of **L7** fluorescence by $Eu(OTf)_3$ in MeCN; λ_{ex} = 440 nm, [**L7**] = 3.4 µM.

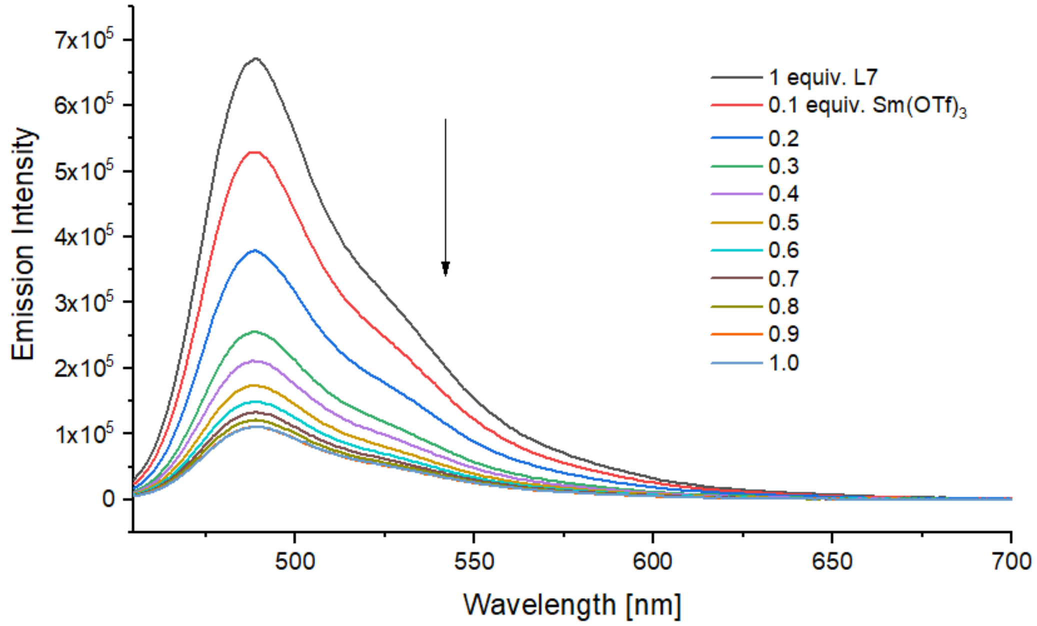

Figure S30. Steady-state fluorescence spectra of **L7** in the presence of increasing amounts of $Sm(OTf)_3$ in MeCN; λ_{ex} = 440 nm, [**L7**] = 3.4 µM.

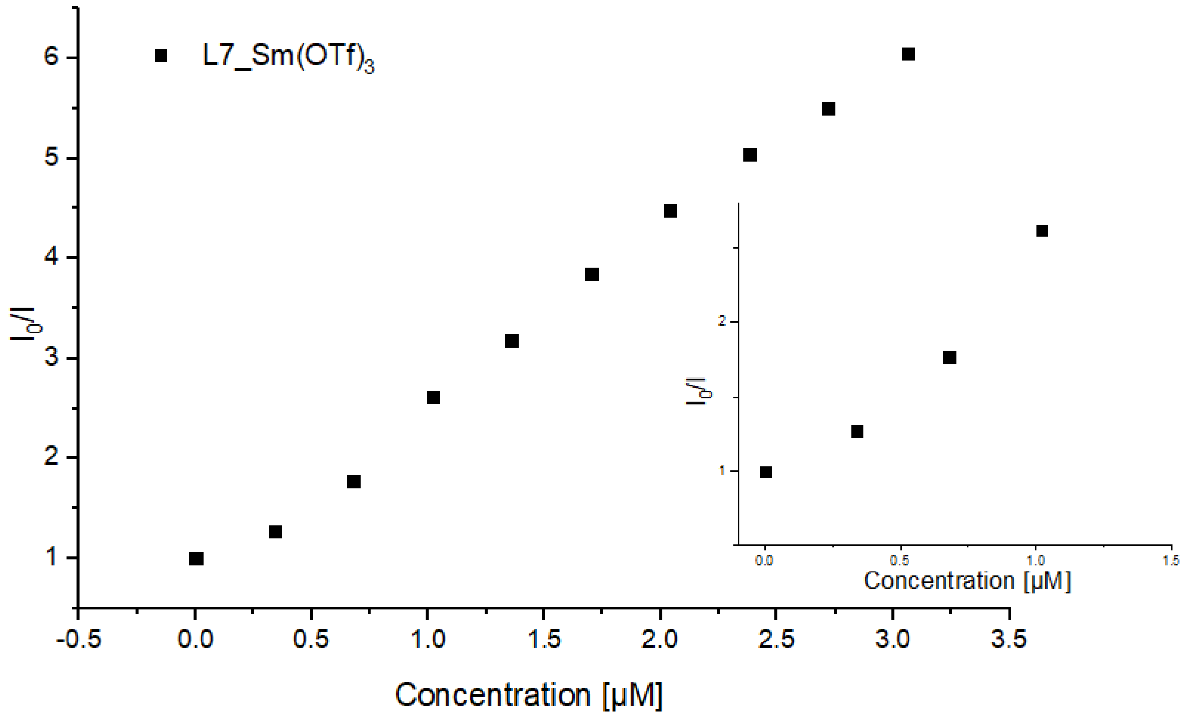

Figure S31. Quenching of **L7** fluorescence by $Sm(OTf)_3$ in MeCN; λ_{ex} = 440 nm, [**L7**] = 3.4 μM.

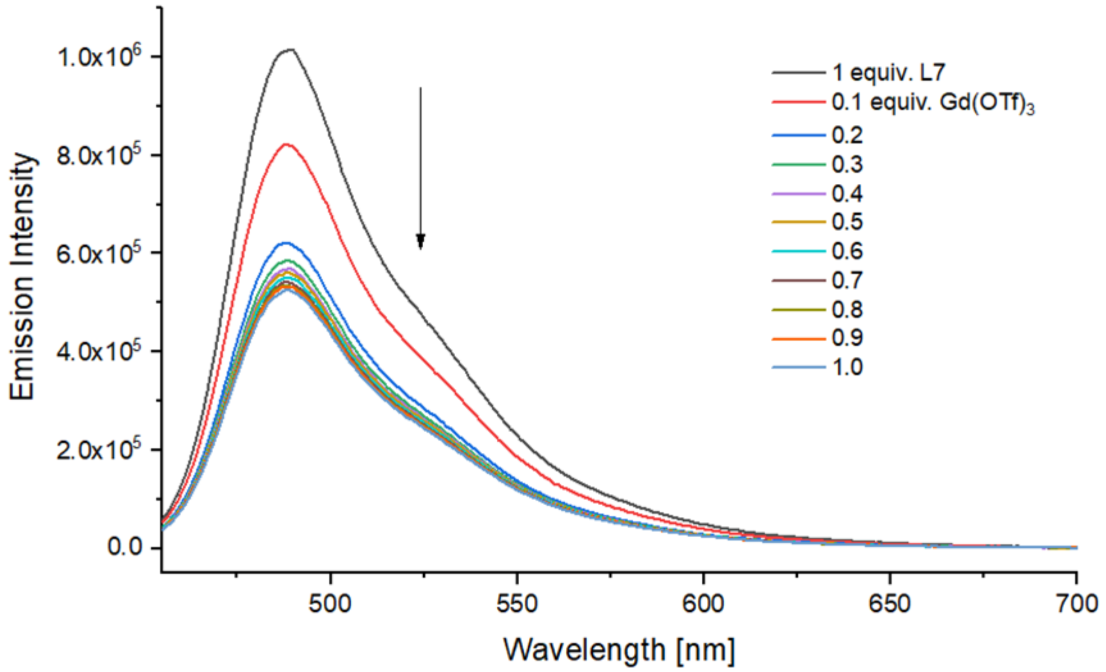

Figure S32. Steady-state fluorescence spectra of **L7** in the presence of increasing amounts of $Gd(OTf)_3$ in MeCN; λ_{ex} = 440 nm, [**L7**] = 3.4 μM.

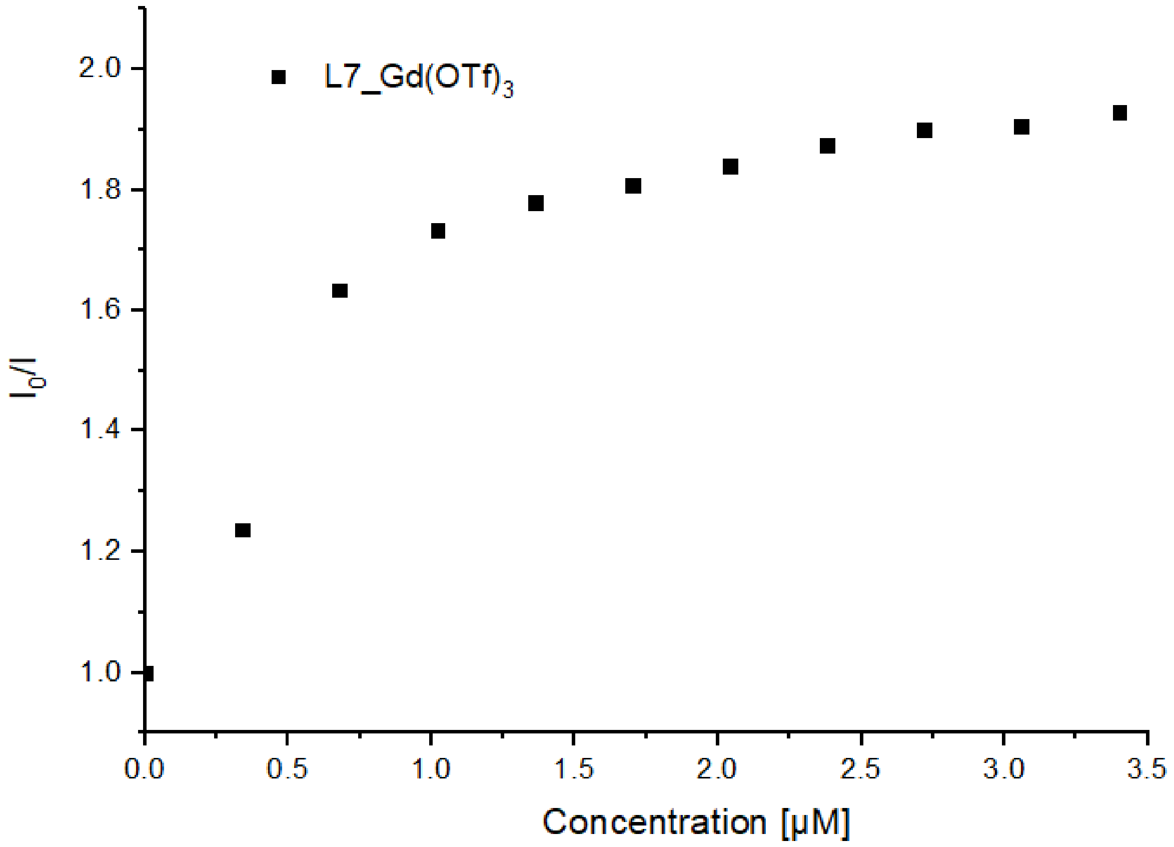

Figure S33. Quenching of **L7** fluorescence by Gd(III) in MeCN; λ_{ex} = 440 nm, [**L7**] = 3.4 μM.

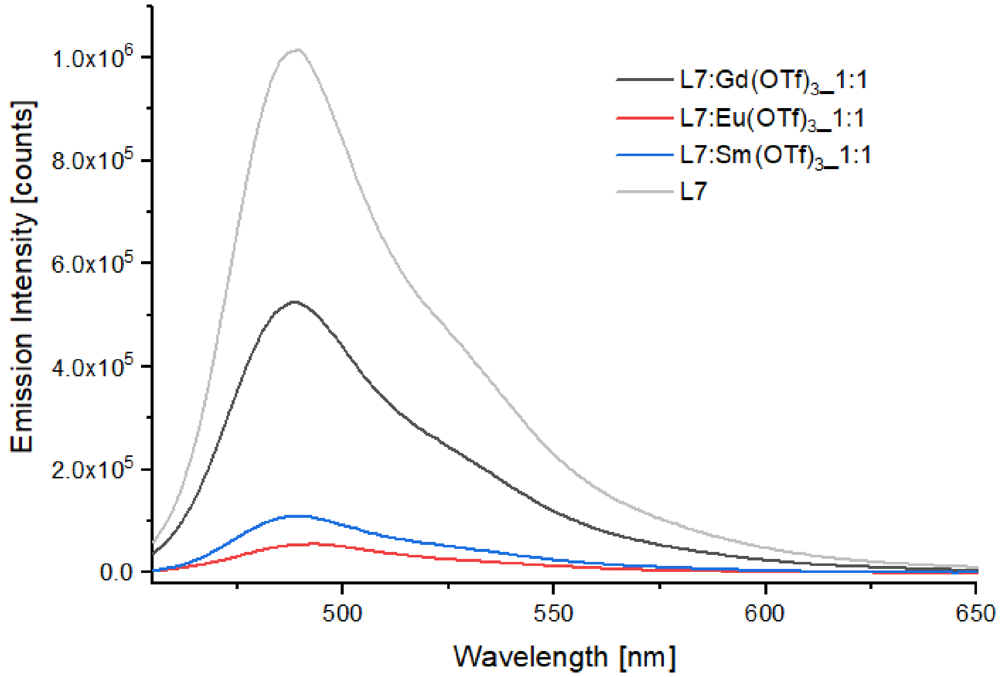

Figure S34. Comparison of steady-state fluorescence spectra of **L7** + $Ln(OTf)_3$ (1:1 ratio) in MeCN; λ_{ex} = 440 nm, [**L7**] = 3.4 μM.

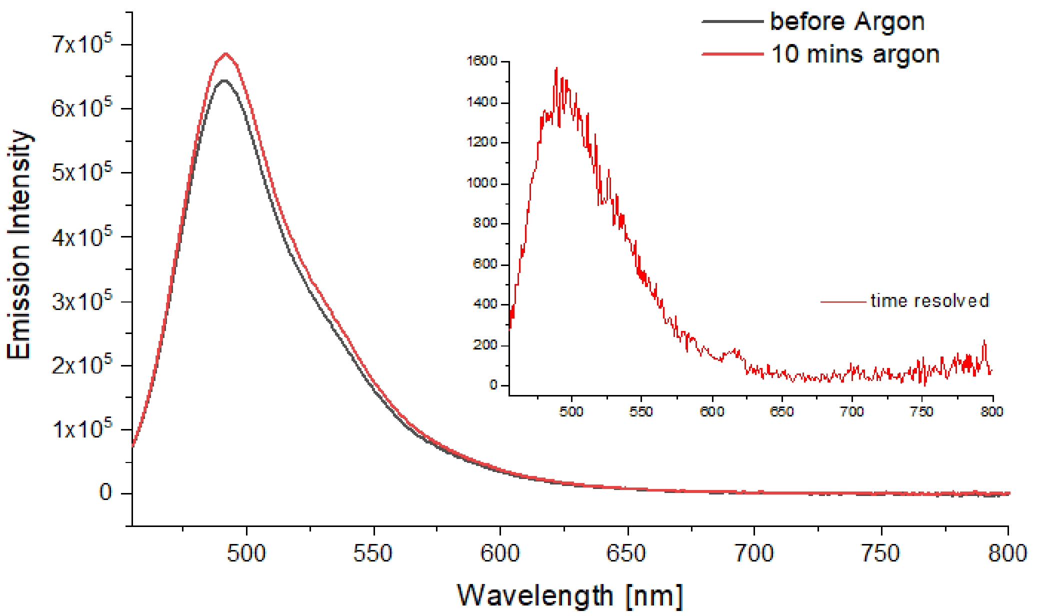

Figure S35. Steady-state and time-resolved fluorescence spectra of **L7** + Eu(OTf)$_3$ (1:1 ratio) before and after a 10 min purge with Ar; DMF, λ_{ex} = 440 nm, [**L7**] = 2 µM.

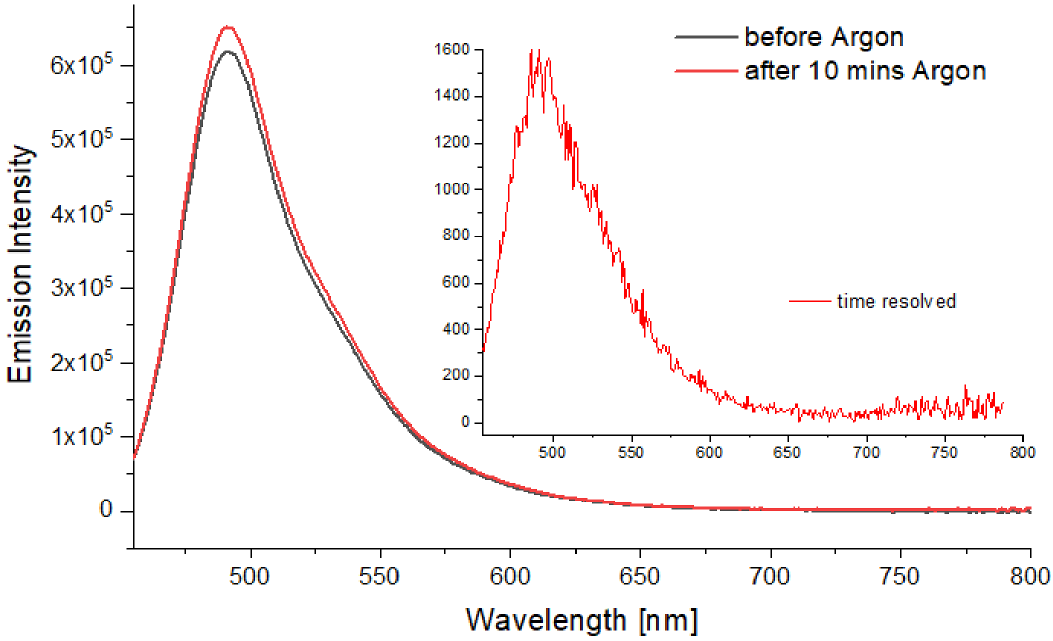

Figure S36. Steady-state and time-resolved fluorescence spectra of **L7** + Sm(OTf)$_3$ (1:1 ratio) before and after a 10 min purge with Ar; DMF, λ_{ex} = 440 nm, [**L7**] = 2 µM.

Quantum yield determinations

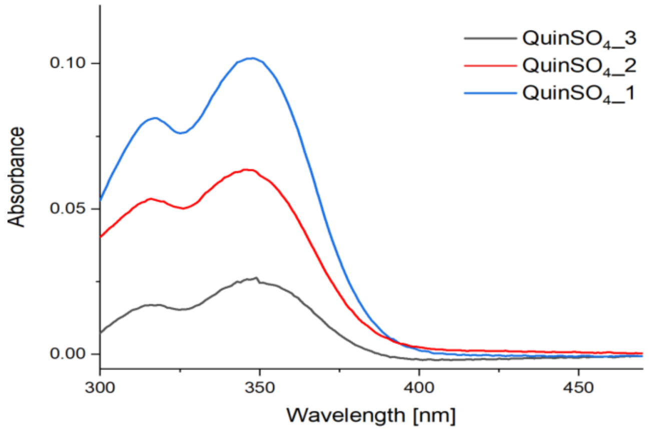

Figure S37. Absorption spectra of quinine sulphate.

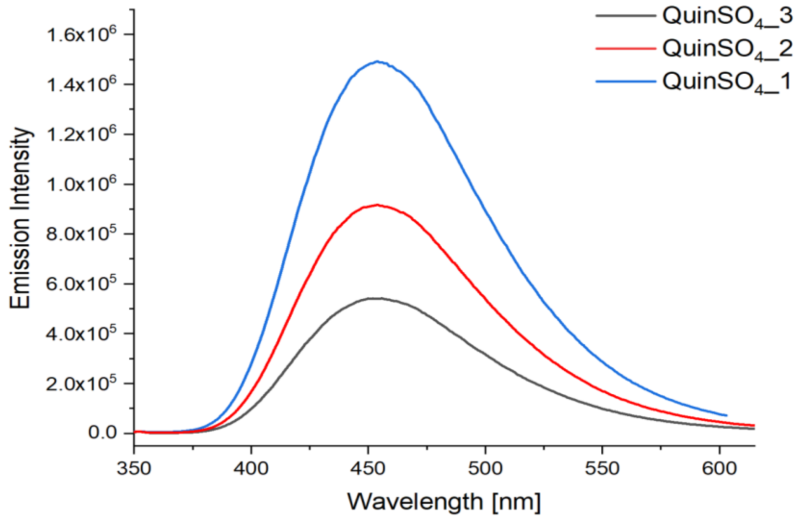

Figure S38. Emission spectra of the quinine sulphate solutions corresponding to the absorption spectra in Figure S37, λ_{ex} = 315 nm.

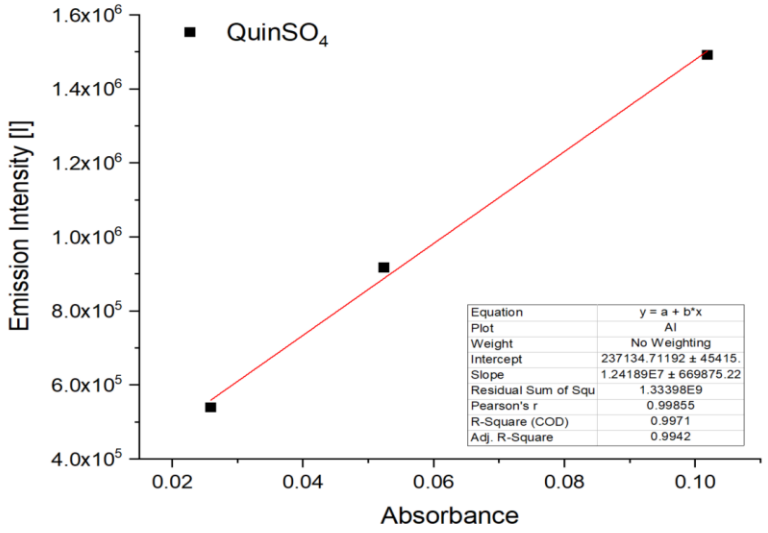

Figure S39. Plot of emission intensity as a function of absorbance of quinine sulphate in DMF.

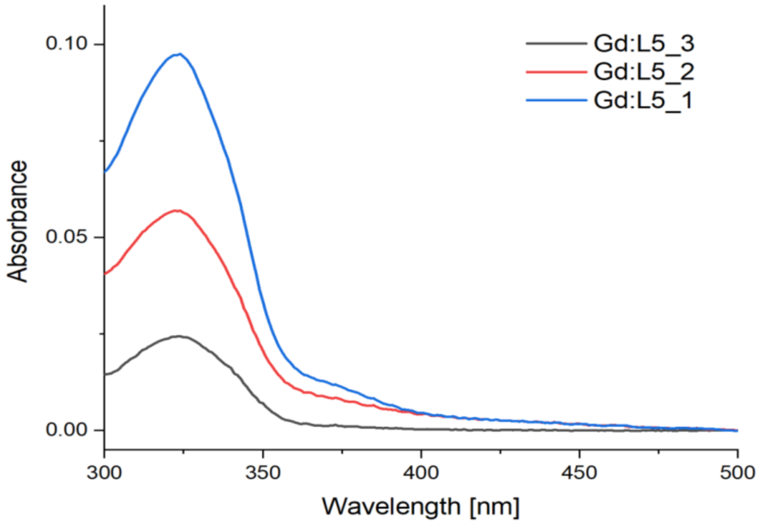

Figure S40. Absorption spectra of Gd:**L5** in DMF.

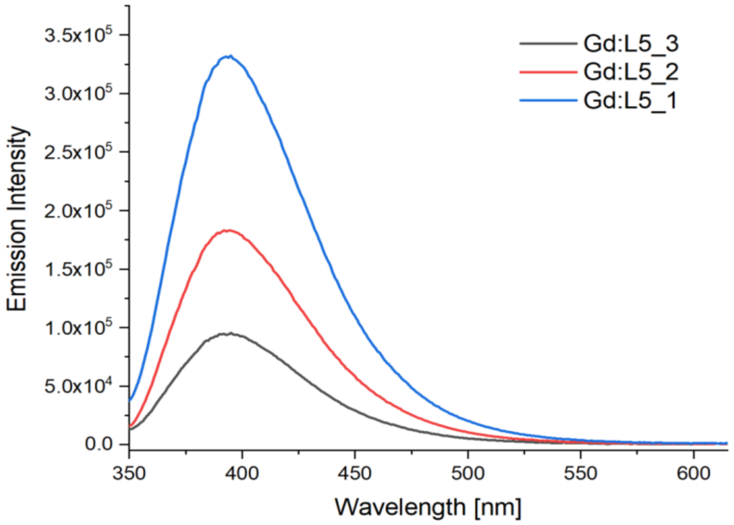

Figure S41. Emission spectra of Gd:**L5** in DMF corresponding to the absorptions in Figure S40, λ_{ex} = 315 nm.

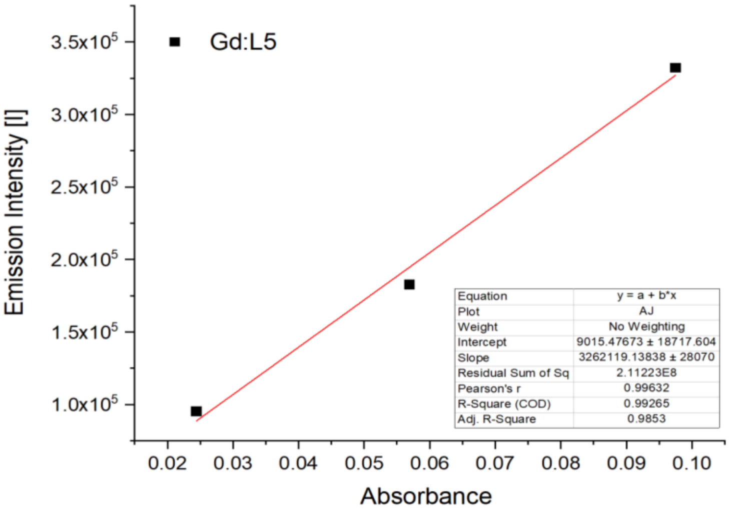

Figure S42. Plot of emission intensity as a function of absorbance of Gd:**L5** (1:1) in DMF.

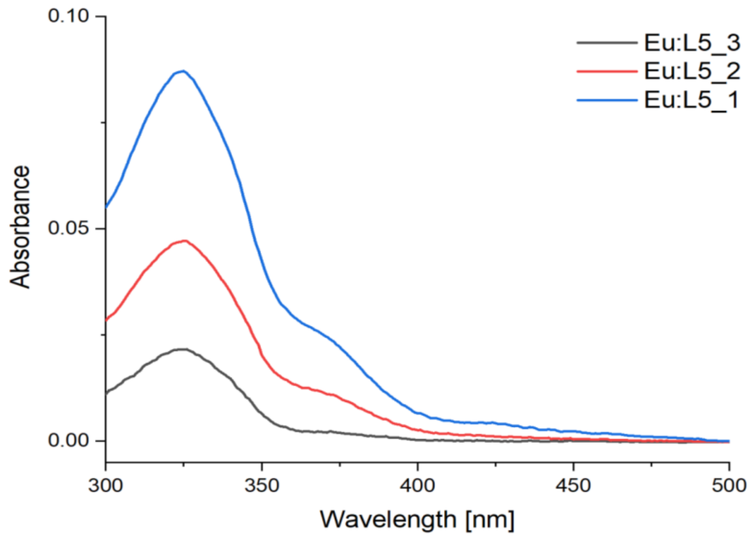

Figure S43. Absorption spectra of Eu:**L5** in DMF.

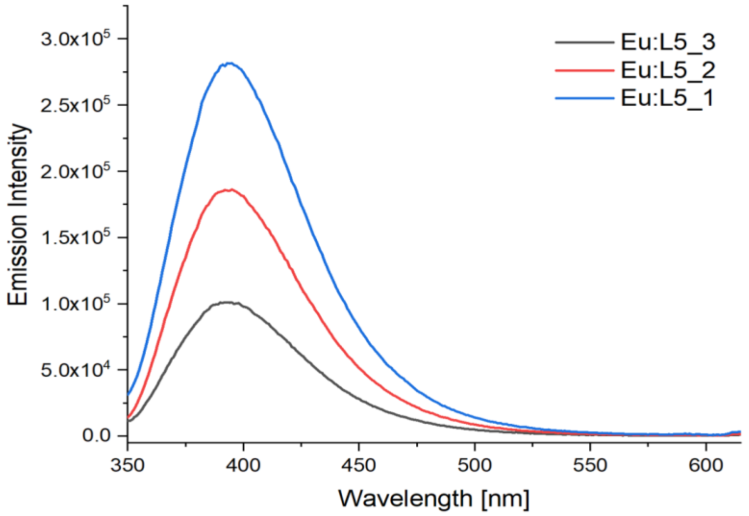

Figure S44. Emission spectra of Eu:**L5** in DMF corresponding to the absorptions in Figure S43, λ_{ex} = 315 nm.

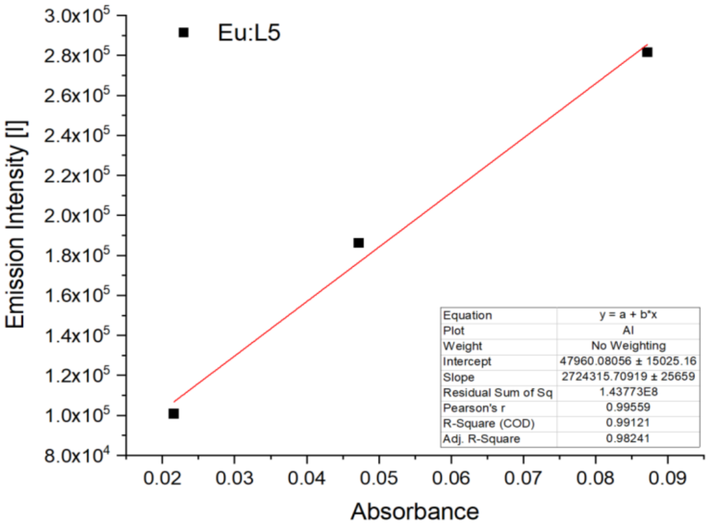

Figure S45. Plot of emission intensity as a function of absorbance of Eu:**L5** (1:1) in DMF.

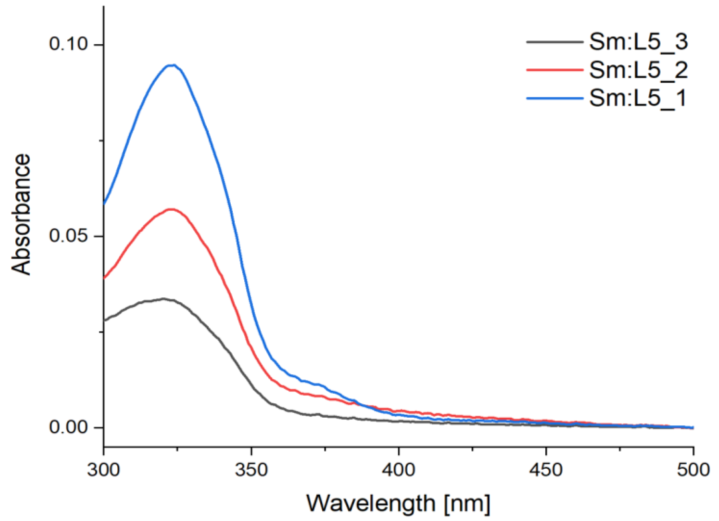

Figure S46. Absorption spectra of Sm:**L5** in DMF.

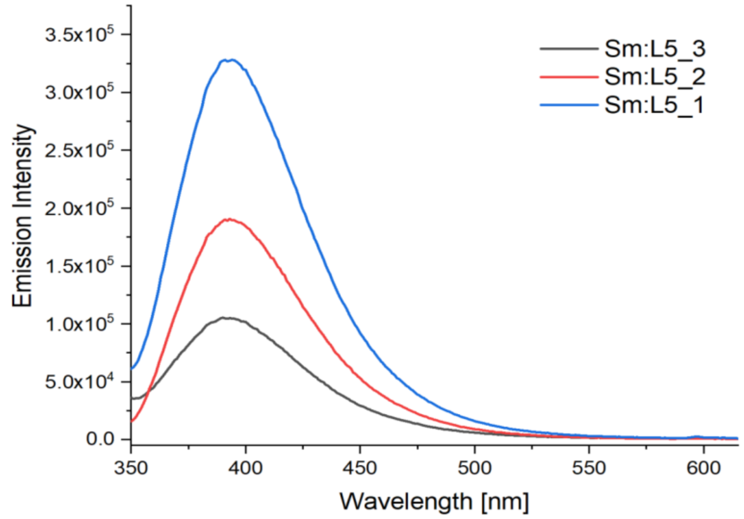

Figure S47. Emission spectra of Sm:**L5** in DMF corresponding to the absorptions in Figure S46, λ_{ex} = 315 nm.

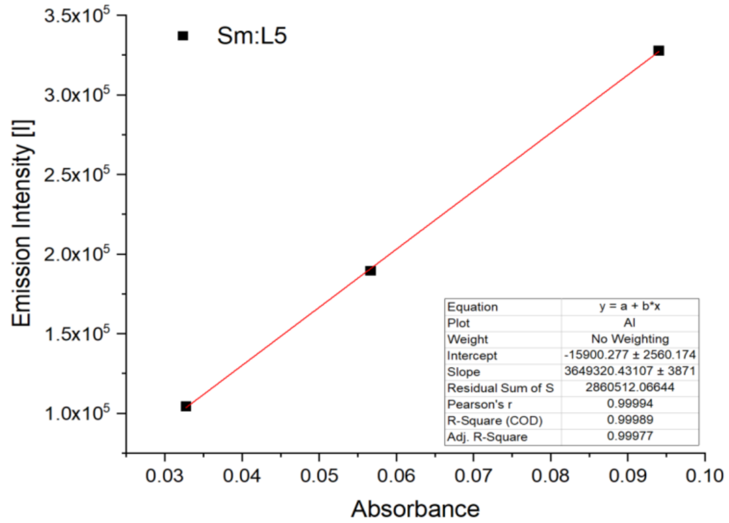

Figure S48. Plot of emission intensity as a function of absorbance of Sm:**L5** (1:1) in DMF.

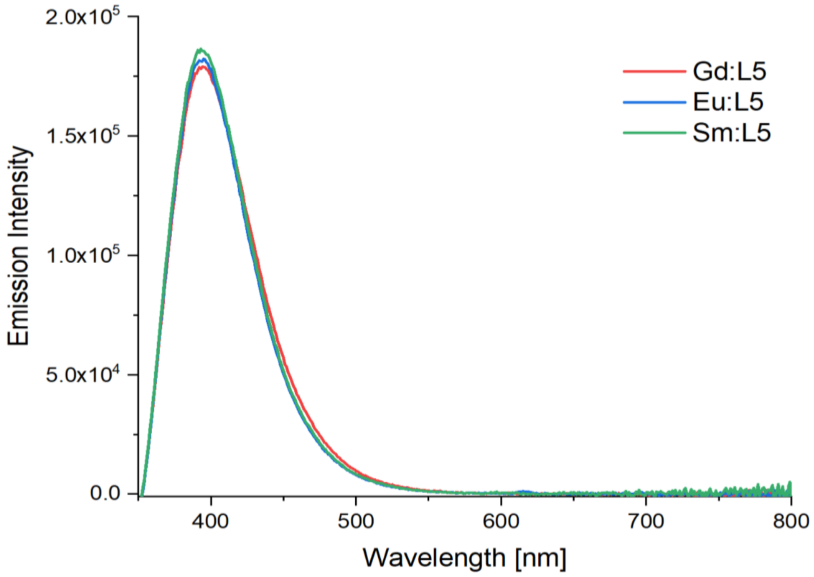

Figure S49. Emission spectra of Ln:**L5** in DMF used for quantum yield calculations, λ_{ex} = 315 nm.

Data for LnL7 without overlapping the absorbance with the reference

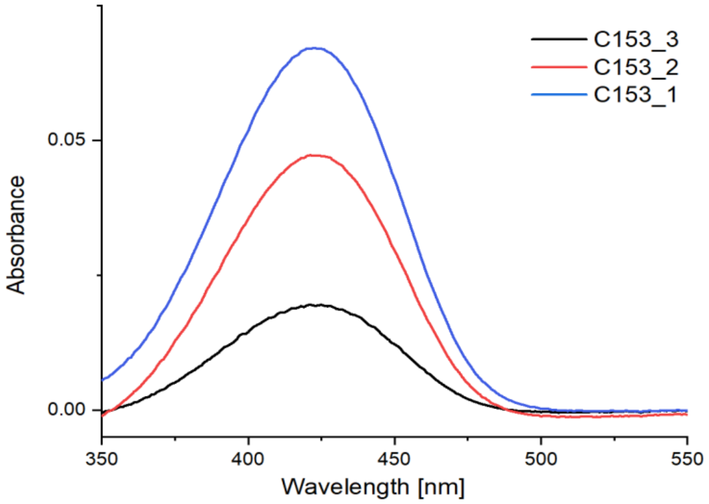

Figure S50. Absorption spectra of **C153** in ethanol.

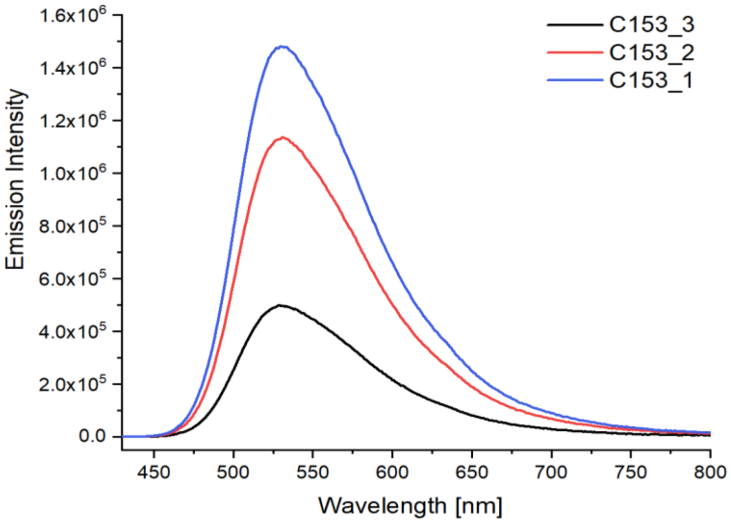

Figure S51. Emission spectra of **C153** in ethanol corresponding to the absorptions in Figure S50, λ_{ex} = 437 nm.

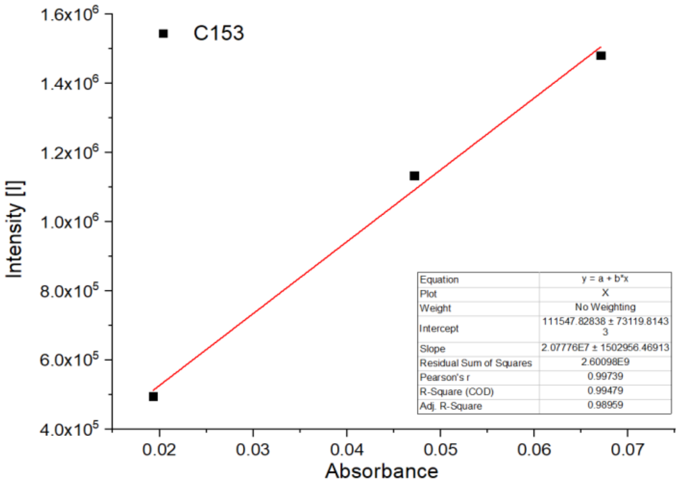

Figure S52. Plot of emission intensity as a function of absorbance of **C153** in ethanol.

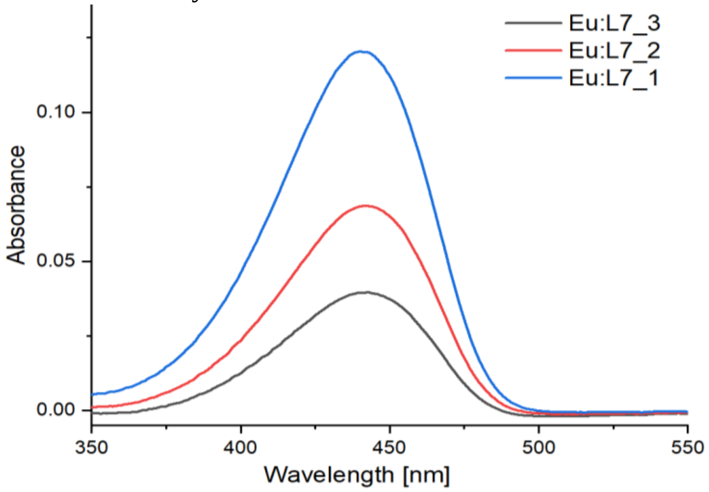

Figure S53. Absorption spectra of Eu:**L7** (1:1) in DMF.

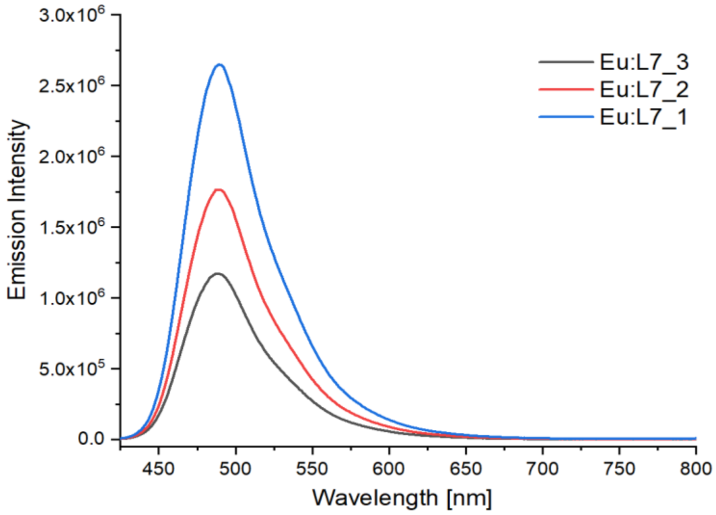

Figure S54. Emission spectra of Eu:**L7** (1:1) in DMF corresponding to the absorptions in Figure S53, λ_{ex} = 437 nm.

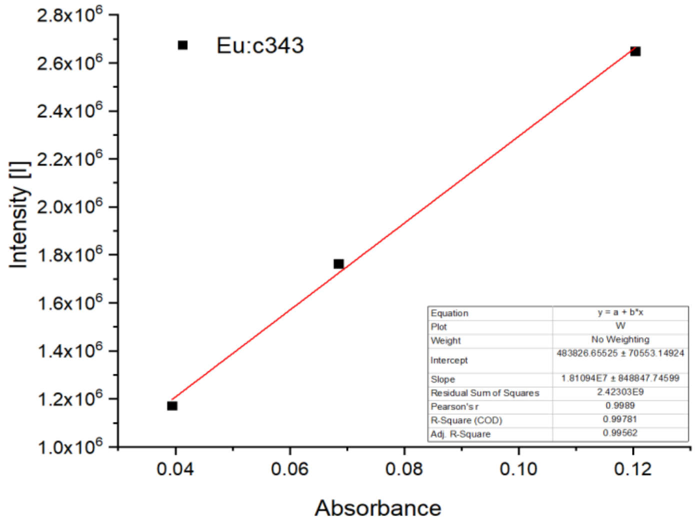

Figure S55. Plot of emission intensity as a function of absorbance of Eu:**L7** (1:1) in DMF.

Data for LnL7 with overlapping the absorbance with the reference

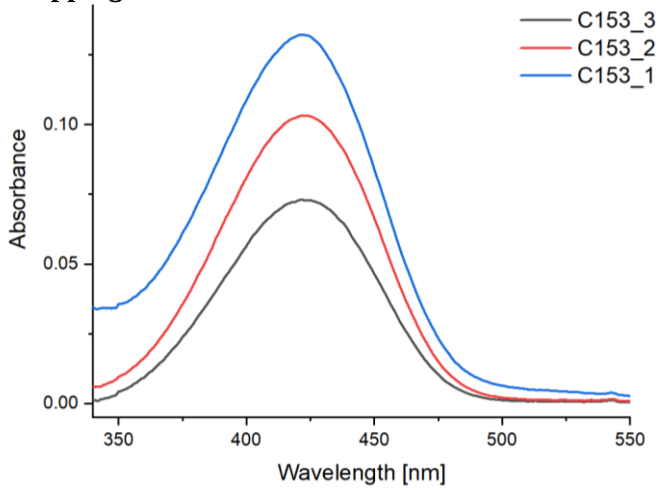

Figure S56. Absorption spectra of **C153** in ethanol.

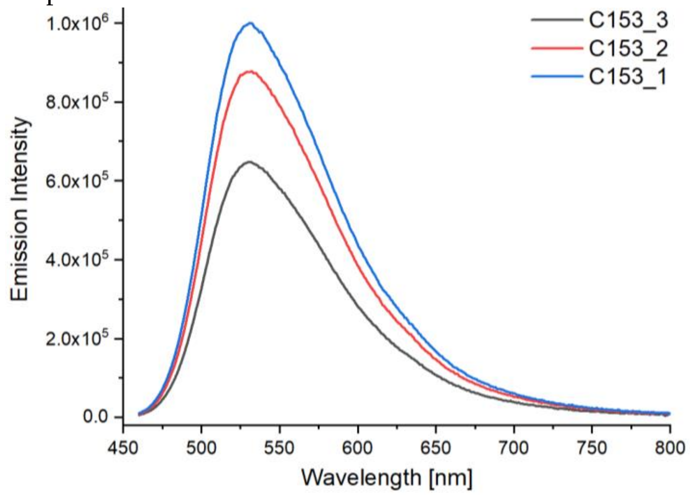

Figure S57. Emission spectra of **C153** in DMF corresponding to the absorptions in Figure S56, λ_{ex} = 447 nm.

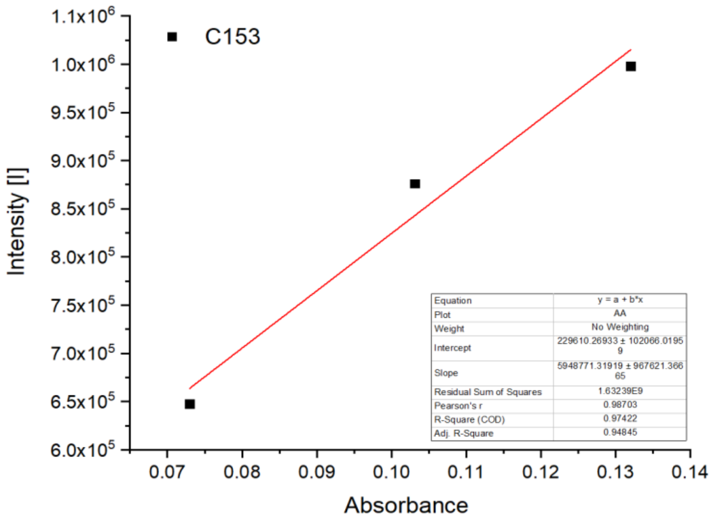

Figure S58. Plot of emission intensity as a function of absorbance of **C-153** in EtOH.

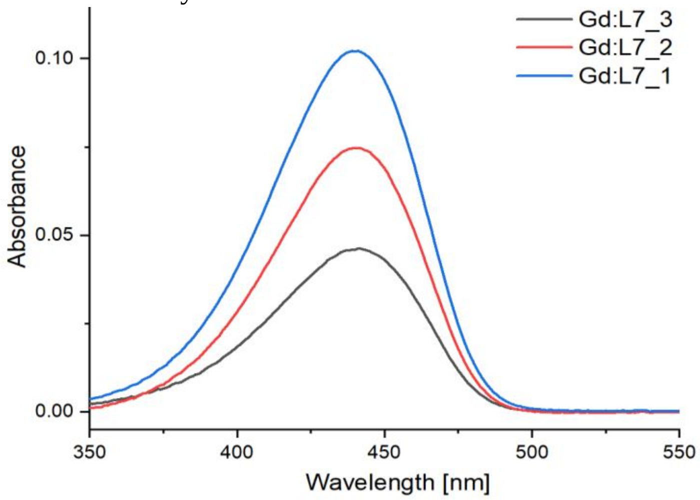

Figure S59. Absorption spectra of Gd:**L7** (1:1) in DMF.

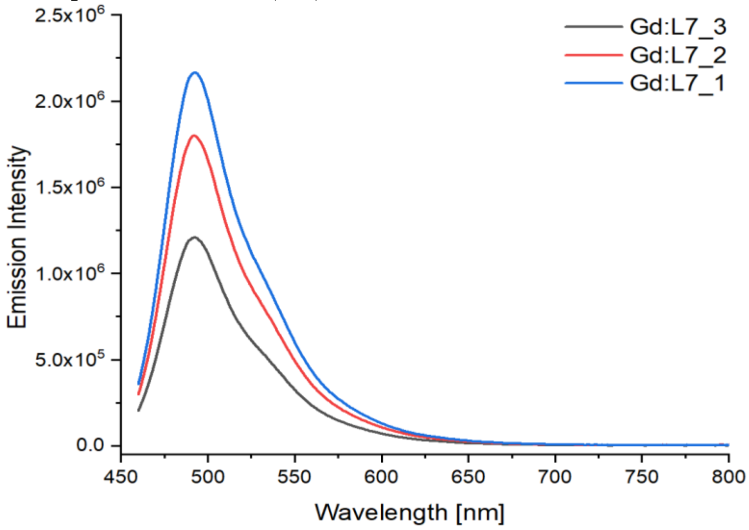

Figure S60. Emission spectra of Gd:**L7** (1:1) in DMF corresponding to the absorptions in Figure S59, λ_{ex} = 447 nm.

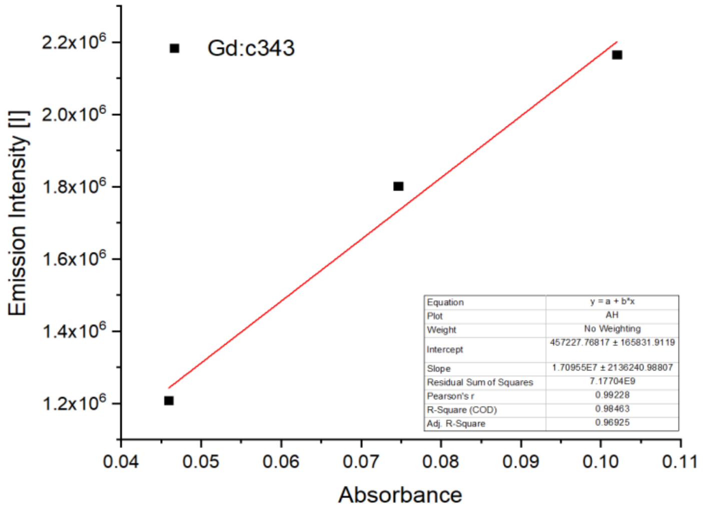

Figure S61. Plot of emission intensity as a function of absorbance of Gd:**L7** (1:1) in DMF

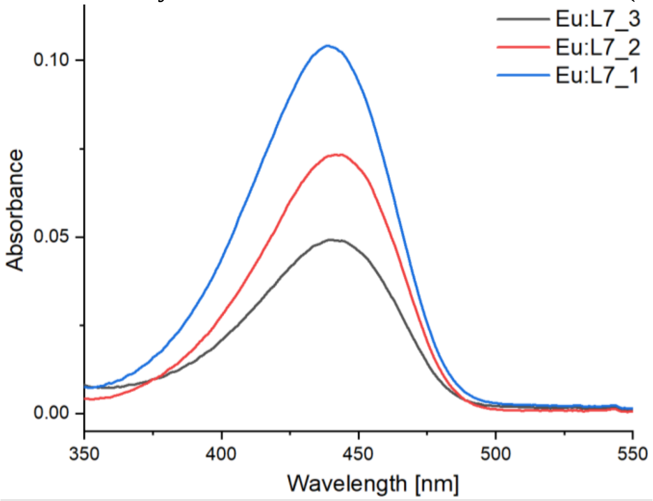

Figure S62. Absorption spectra of Eu:**L7** (1:1) in DMF.

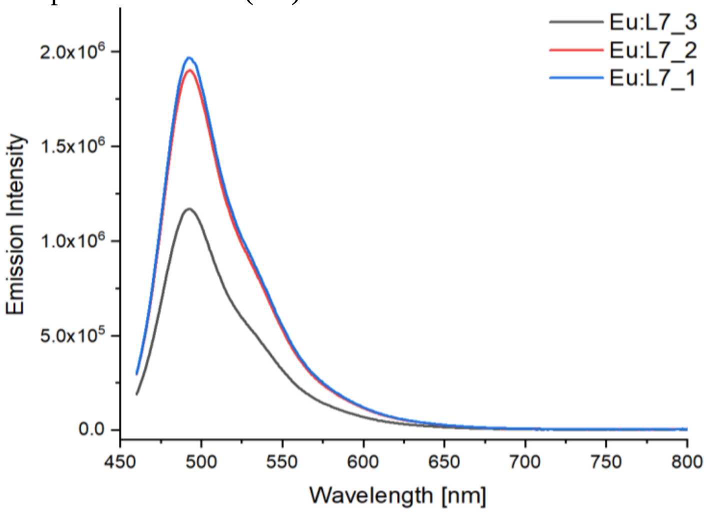

Figure S63. Emission spectra of Eu:**L7** (1:1) in DMF corresponding to the absorptions in Figure S62, λ_{ex} = 447 nm.

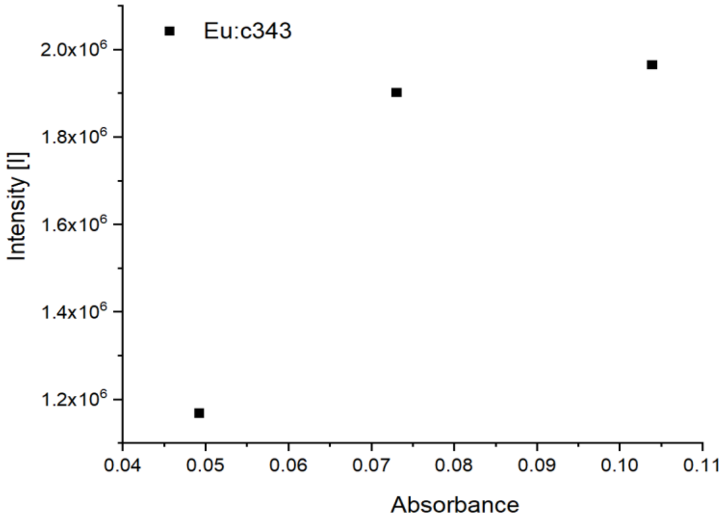

Figure S64. Plot of emission intensity as a function of absorbance of Eu:**L7** (1:1) in DMF.

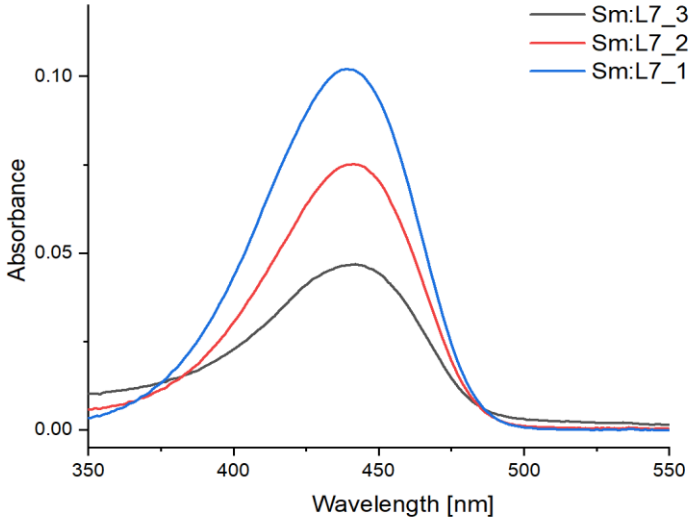

Figure S65. Absorption spectra of Sm:**L7** (1:1) in DMF.

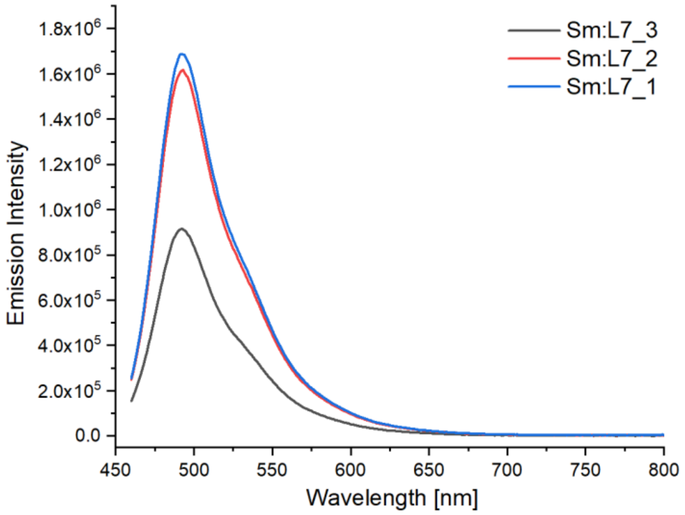

Figure S66. Emission spectra of Sm:**L7** (1:1) in DMF corresponding to the absorptions in Figure S65, λ_{ex} = 447 nm.

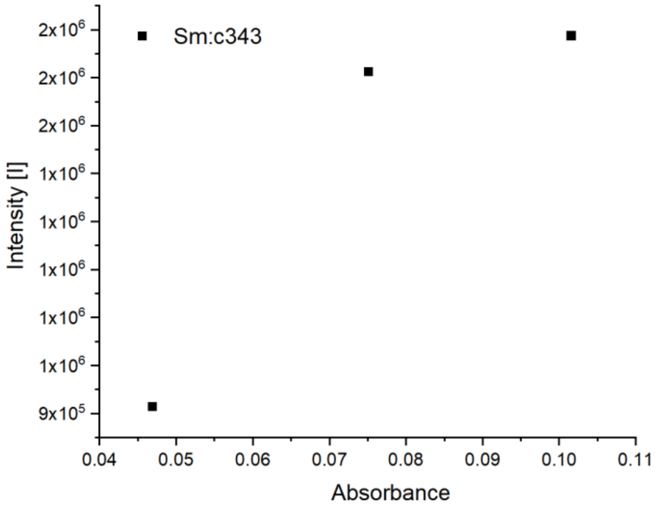

Figure S67. Plot of emission intensity as a function of absorbance of Sm:**L7** (1:1) in DMF.

Photophysical characterization of the irradiated Ln(III)-L mixtures

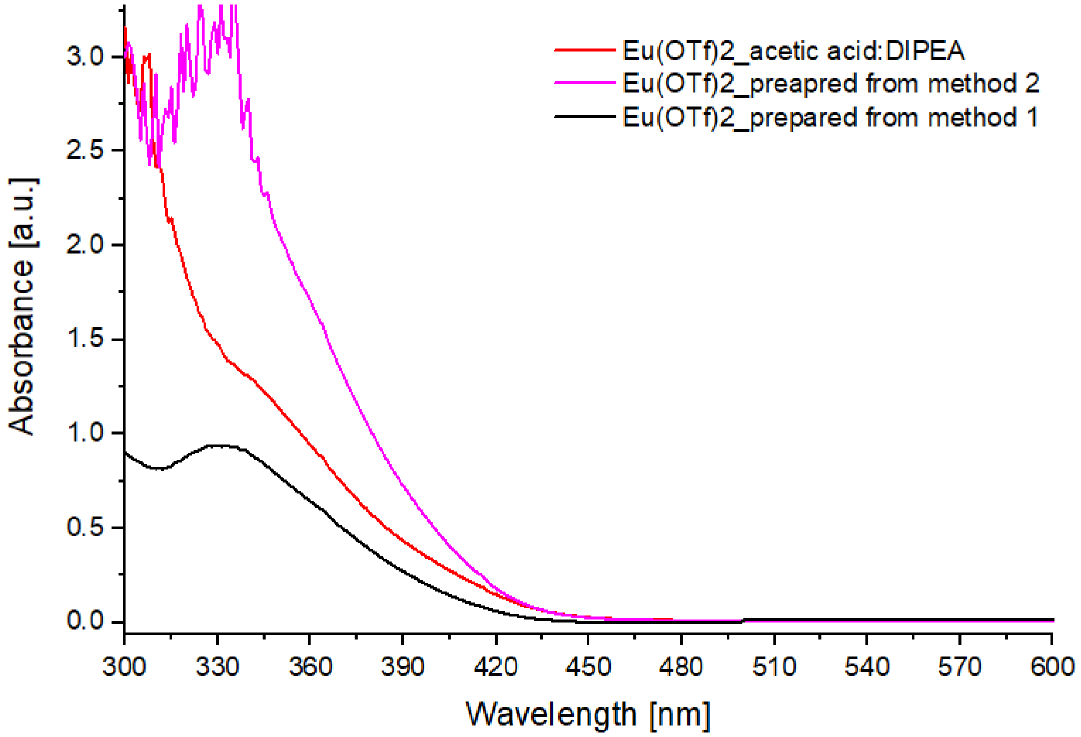

Figure S68. Absorption spectra of $Eu(OTf)_2$ prepared using two different methods (see Materials and methods, ~1 mM) in DMF.

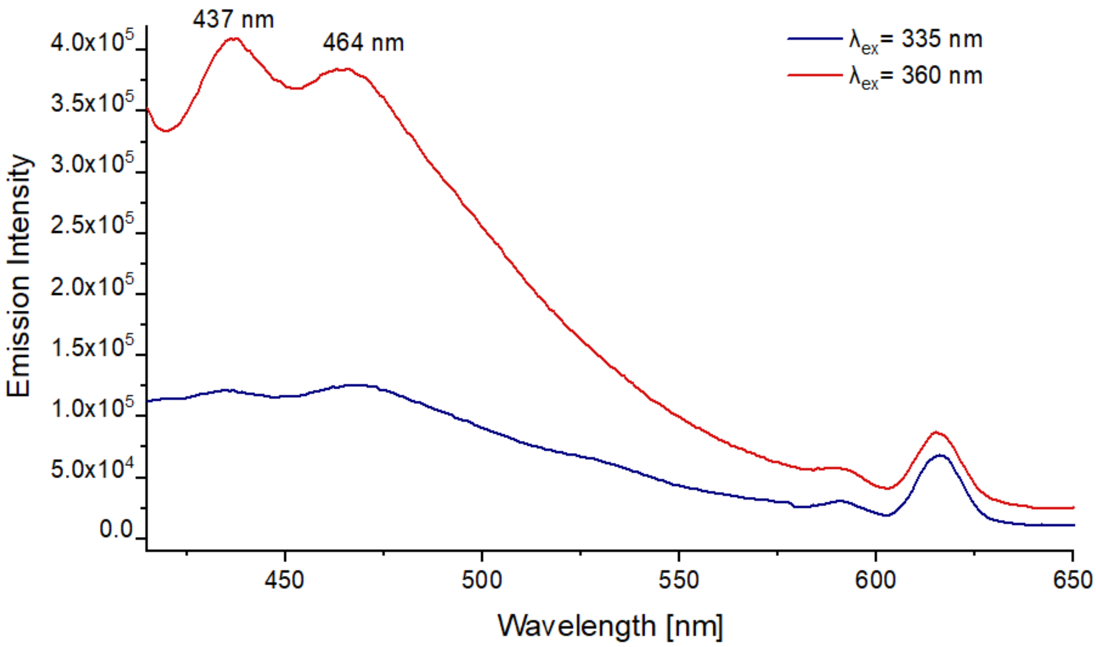

Figure S69. Emission spectra of $Eu(OTf)_2$ prepared by method 1 (~1 mM) in DMF at different excitation wavelengths.

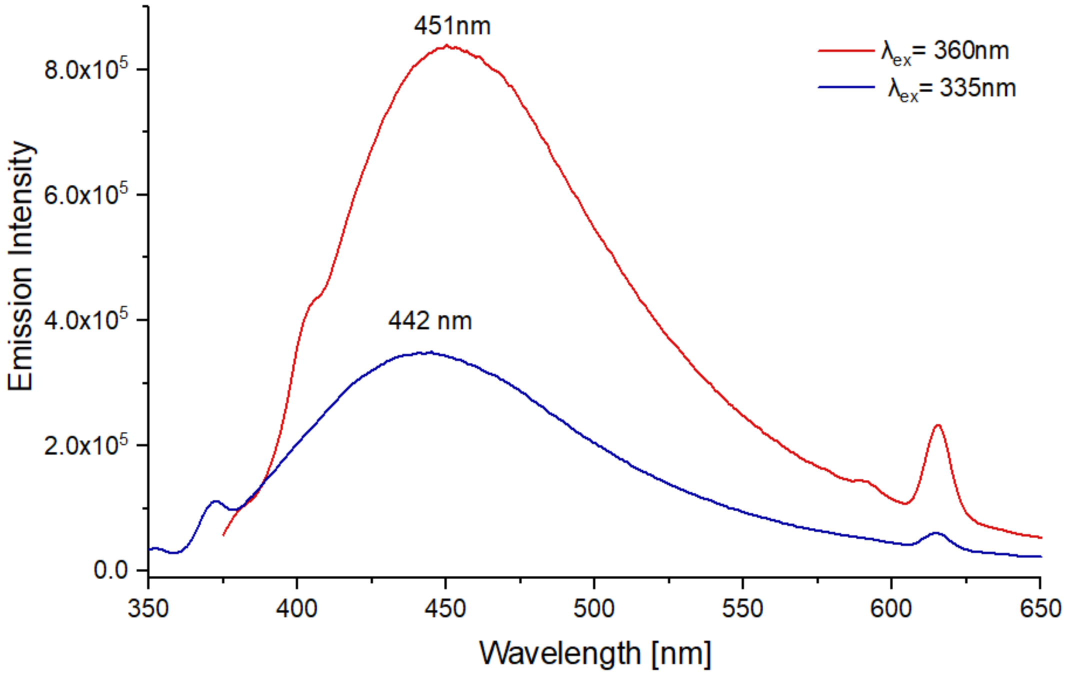

Figure S70. Emission spectra of $Eu(OTf)_2$ in the presence of acetic acid and DIPEA, prepared by method 1 (~1 mM) in DMF, at different excitation wavelengths.

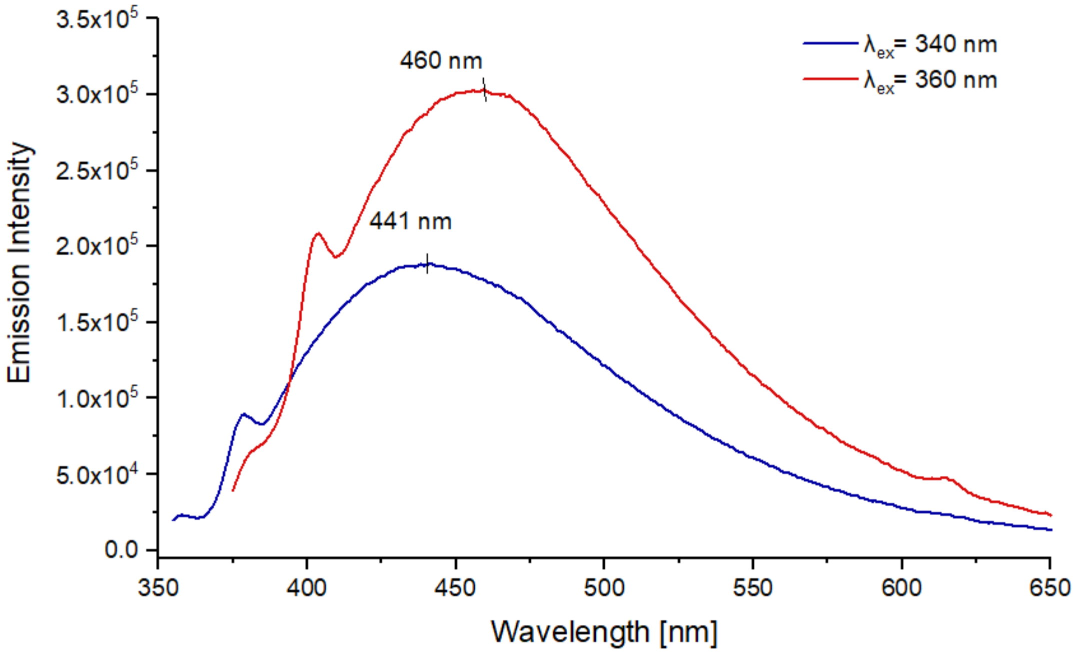

Figure S71. Emission spectra of $Eu(OTf)_2$ in presence of acetic acid and DIPEA, prepared using method 2 (~1 mM) in DMF, at different excitation wavelengths.

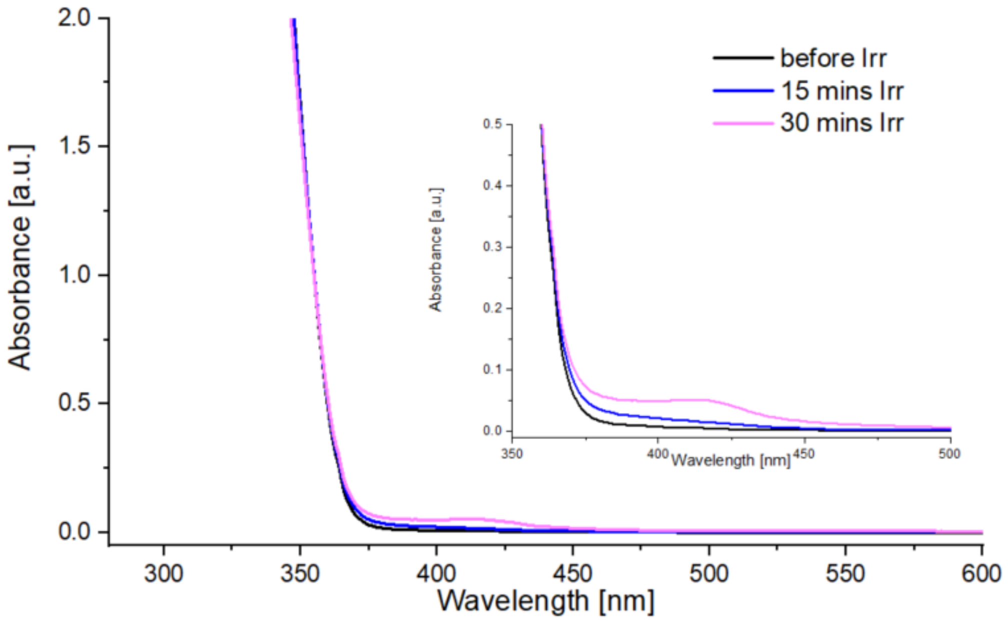

Figure S72. Absorption spectra of **L4** + $Eu(OTf)_3$ (1:1 ratio, 0.3 mM) in the presence of 5.7 mM DIPEA in DMF.

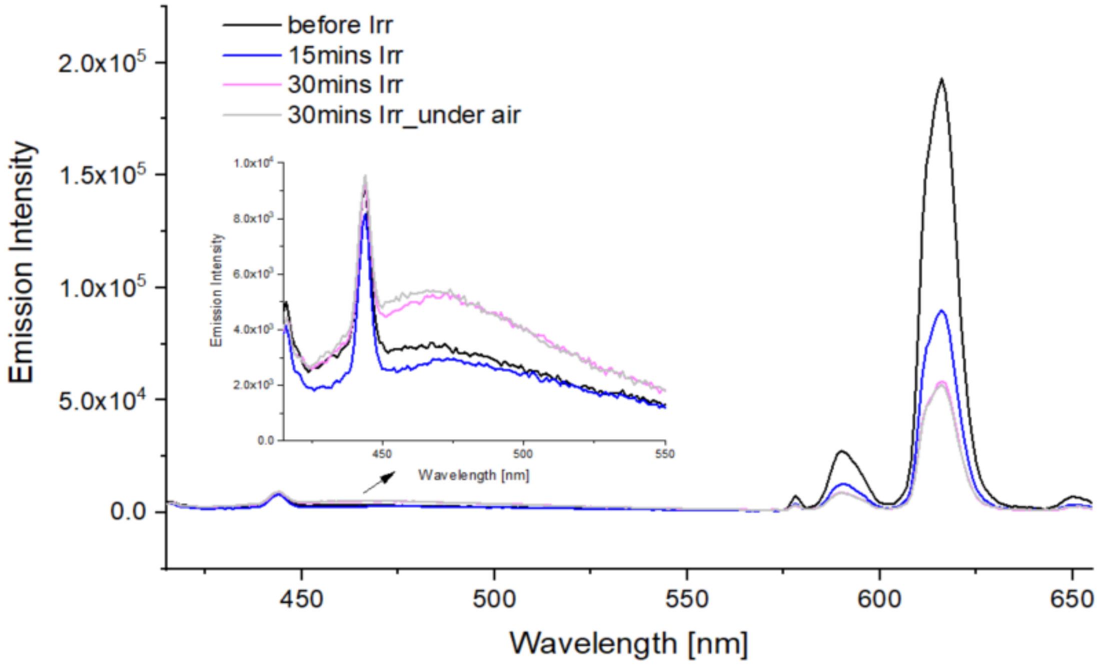

Figure S73. Emission spectra of **L4** + $Eu(OTf)_3$ (1:1 ratio, 0.3 mM) in the presence of 5.7 mM DIPEA in DMF on direct excitation, λ_{ex} = 393 nm.

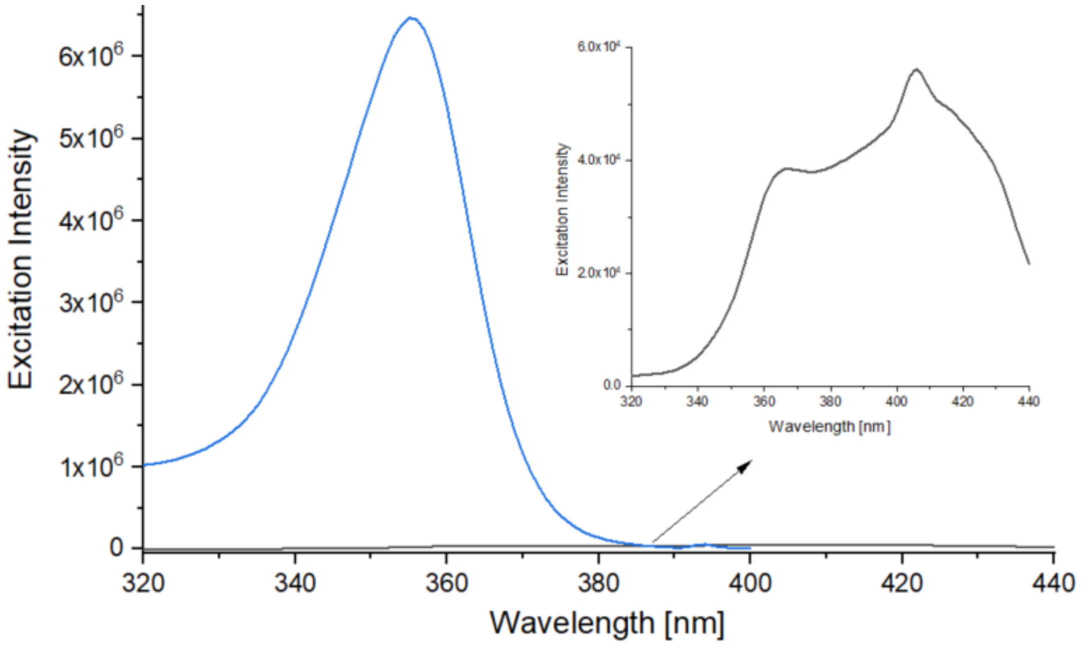

Figure S74. Excitation spectra of **L4** + Eu(OTf)$_3$ (1:1 ratio, 0.3 mM) in the presence of 5.7 mM DIPEA in DMF; blue: λ_{em} = 616 nm, black: λ_{em} = 460 nm.

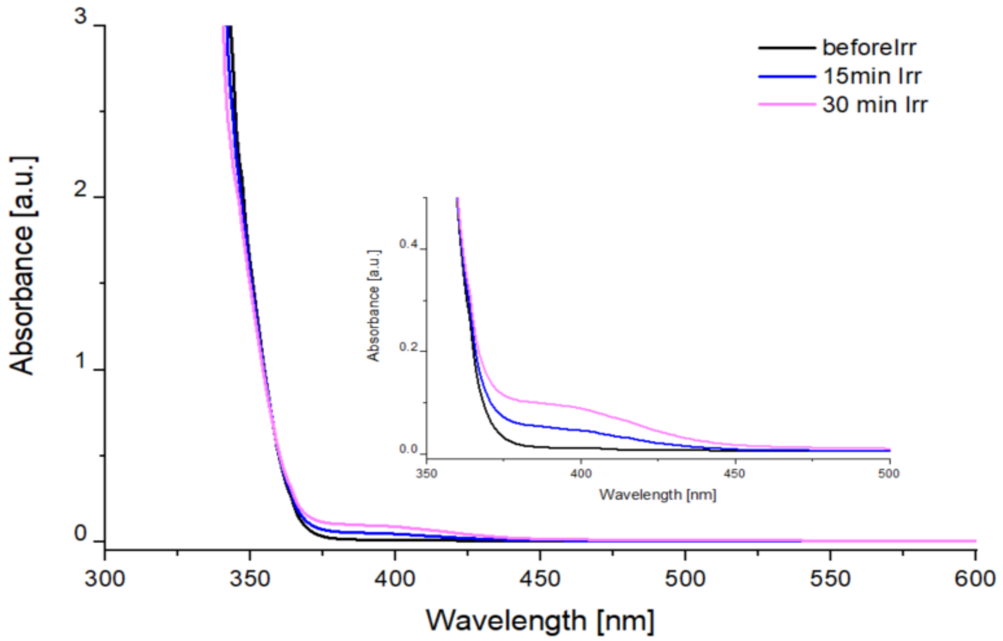

Figure S75. Absorption spectra of **L4** + Sm(OTf)$_3$ (1:1 ratio, 0.3 mM) in the presence of 5.7 mM DIPEA in DMF.

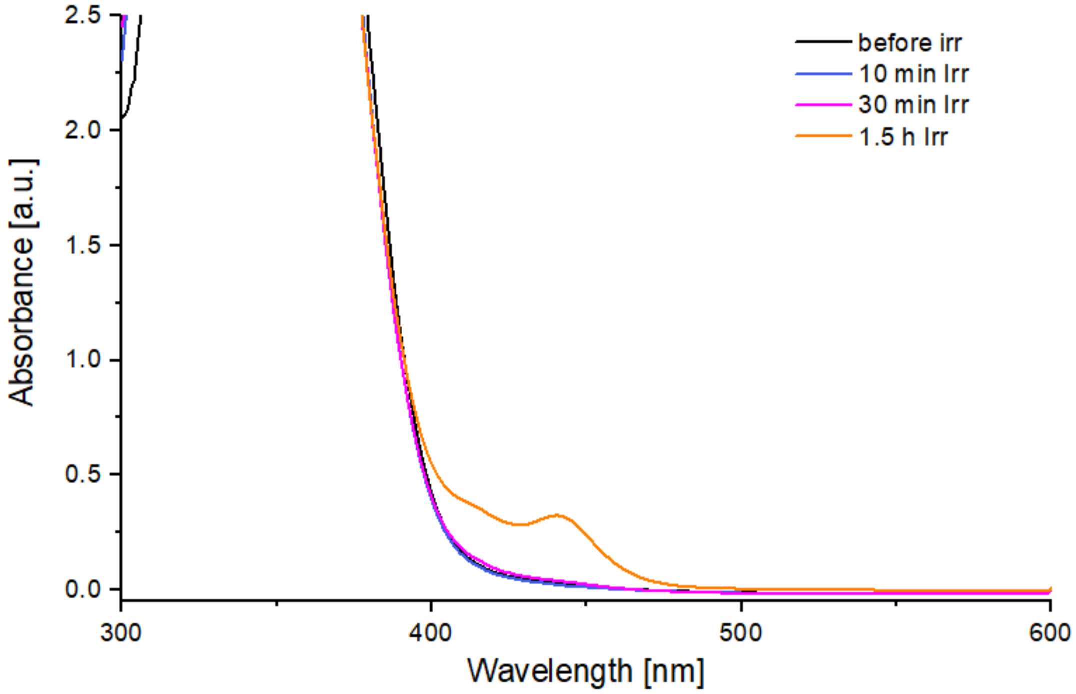

Figure S76. Absorption spectra of **L5** + Eu(OTf)$_3$ (1:1 ratio, 0.3 mM) in the presence of 5.7 mM DIPEA in DMF. The peak emerging at 440 nm after 1.5 h is irreversible.

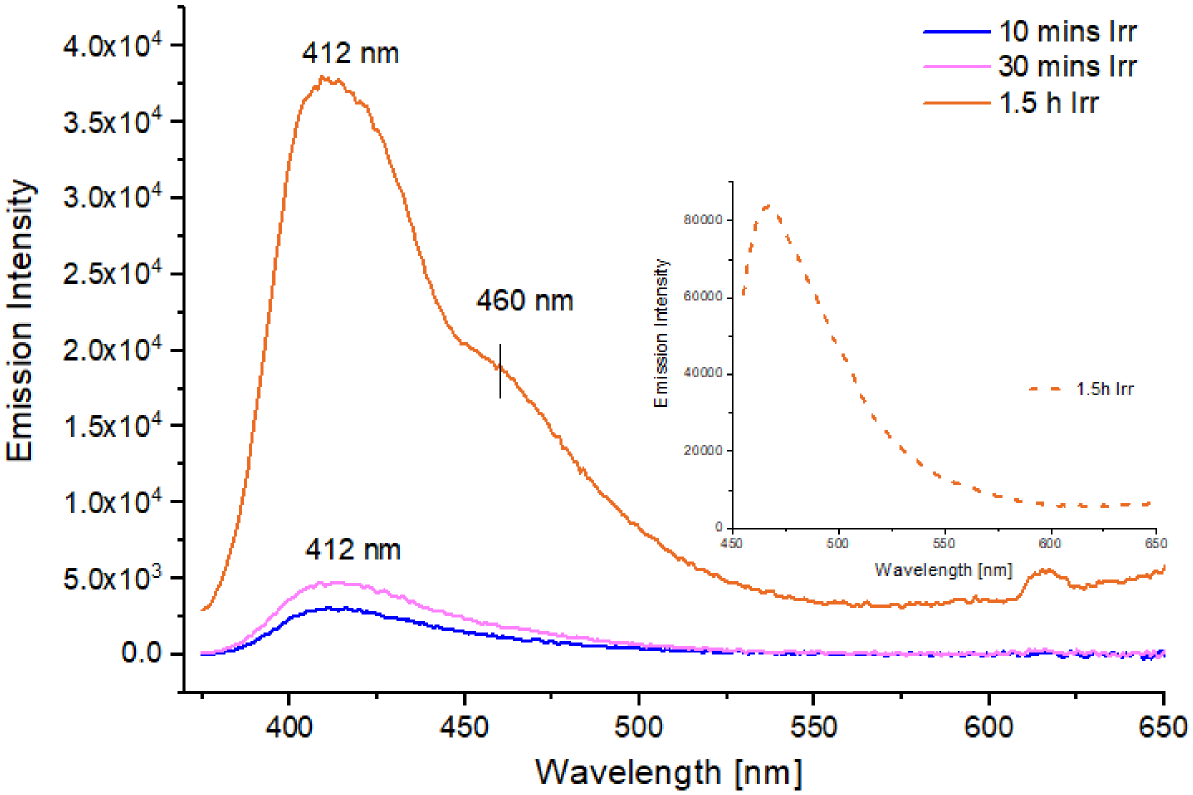

Figure S77. Emission spectra of **L5** + Eu(OTf)$_3$ (1:1 ratio, 0.3 mM) in the presence of 5.7 mM DIPEA in DMF; λ_{ex} = 360 nm; inset shows the emission excited at λ_{ex} = 440 nm.

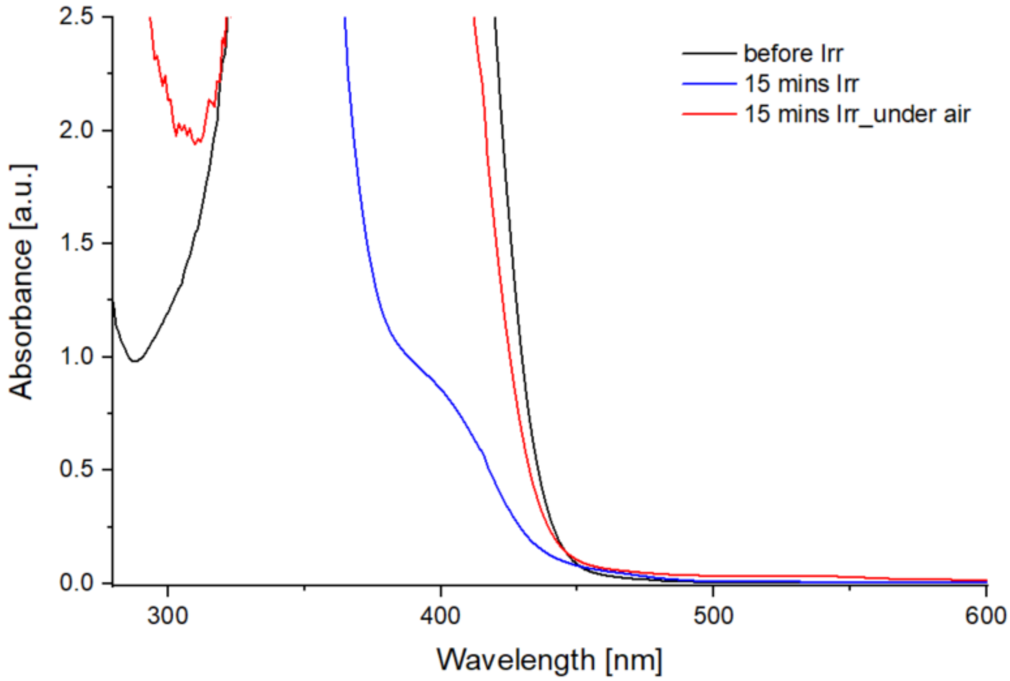

Figure S78. Absorption spectra of **L6** + $Eu(OTf)_3$ (1:1 ratio, 0.3 mM) in the presence of 5.7 mM DIPEA in DMF.

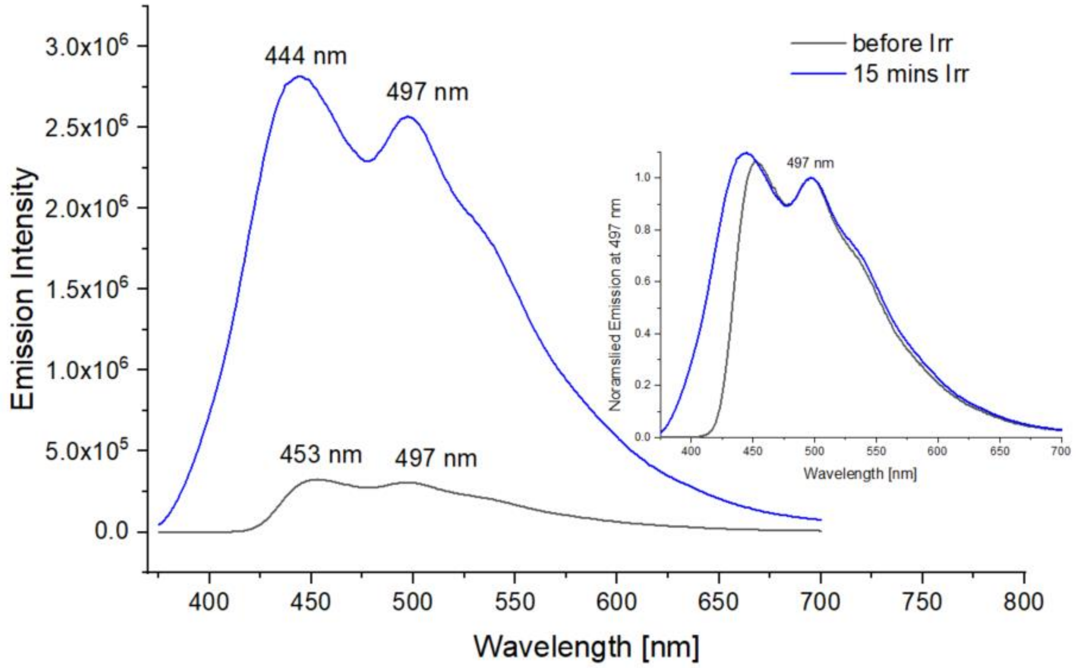

Figure S79. Emission spectra of **L6** + $Eu(OTf)_3$ (1:1 ratio, 0.3 mM) in the presence of 5.7 mM DIPEA in DMF; λ_{ex} = 360 nm; inset shows the spectra normalised at 497 nm.

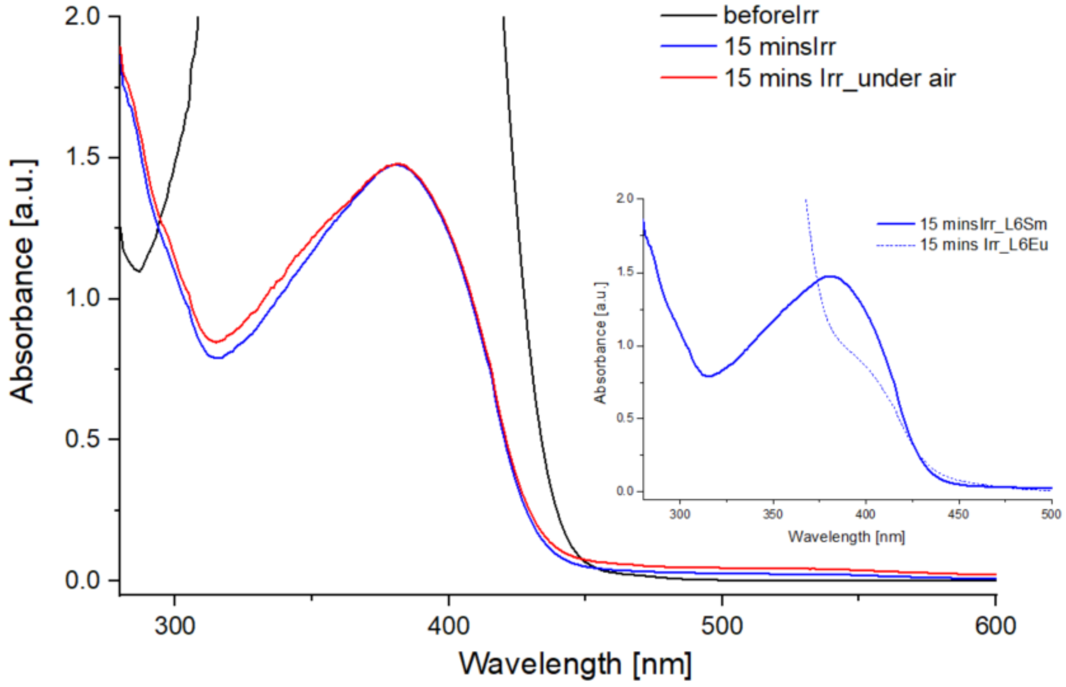

Figure S80. Absorption spectra of **L6** + $Sm(OTf)_3$ (1:1 ratio, 0.3 mM) in the presence of 5.7 mM DIPEA in DMF; inset shows the compared absorption spectra of **L6**:Ln after 15 mins of irradiation (solid blue, **L6:**Sm and dashed blue line, **L6:**Eu).

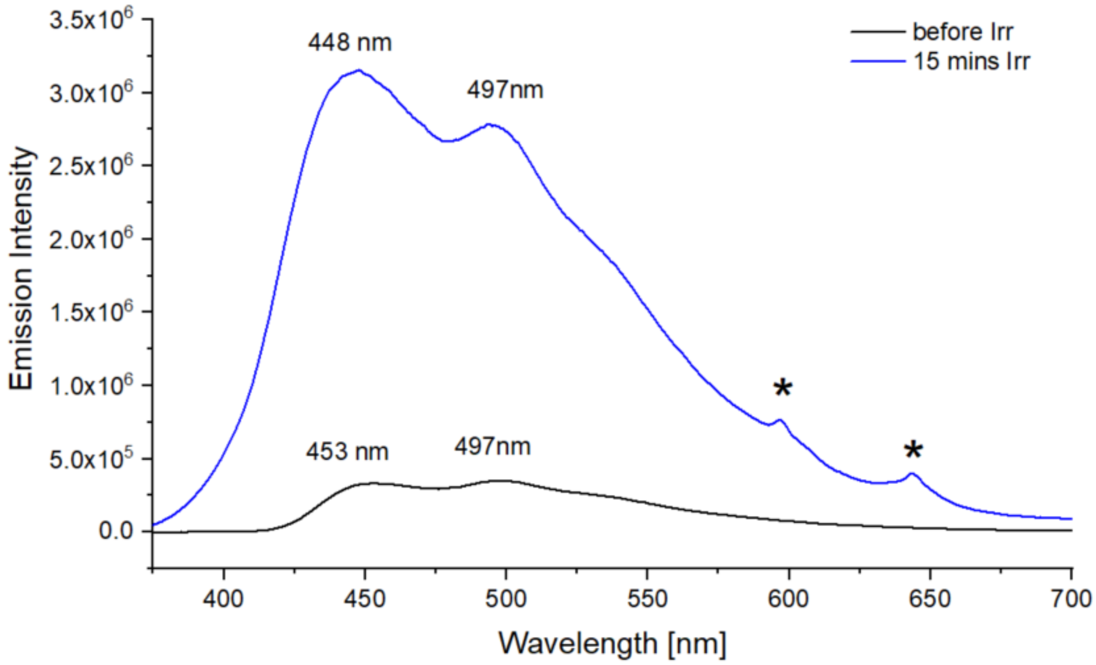

Figure S81. Emission spectra of **L6** + $Sm(OTf)_3$ (1:1 ratio, 0.3 mM) in the presence of 5.7 mM DIPEA in DMF; blue, λ_{exc} = 360 nm; * marked shows the Sm(III) emission.

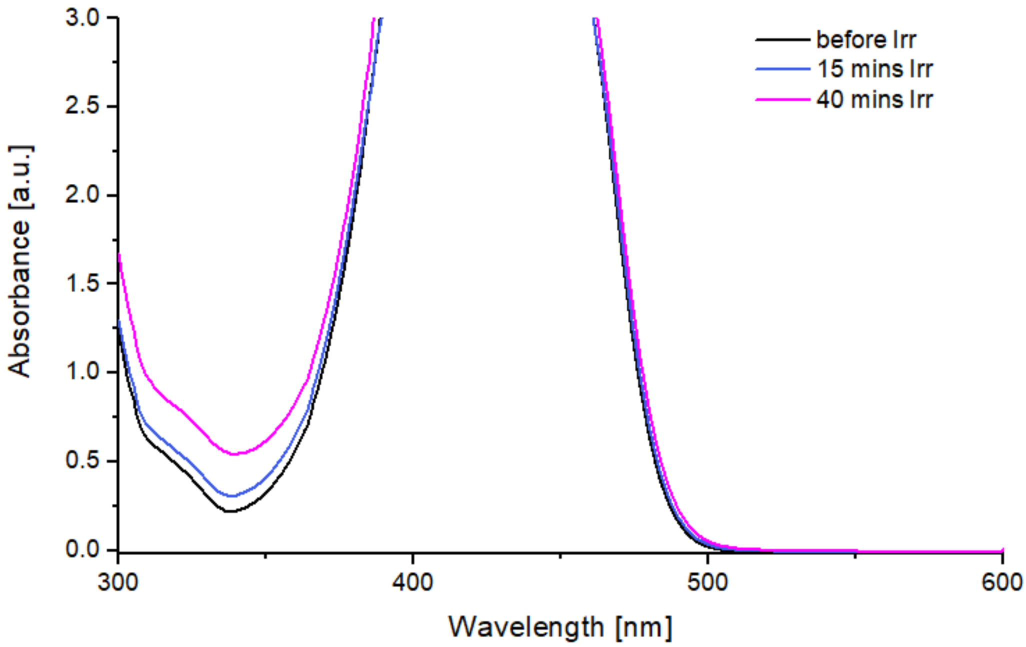

Figure S82. Absorption spectra of **L7** + Eu(OTf)$_3$ (1:1 ratio, 0.3 mM) in the presence of 5.7 mM DIPEA in DMF.

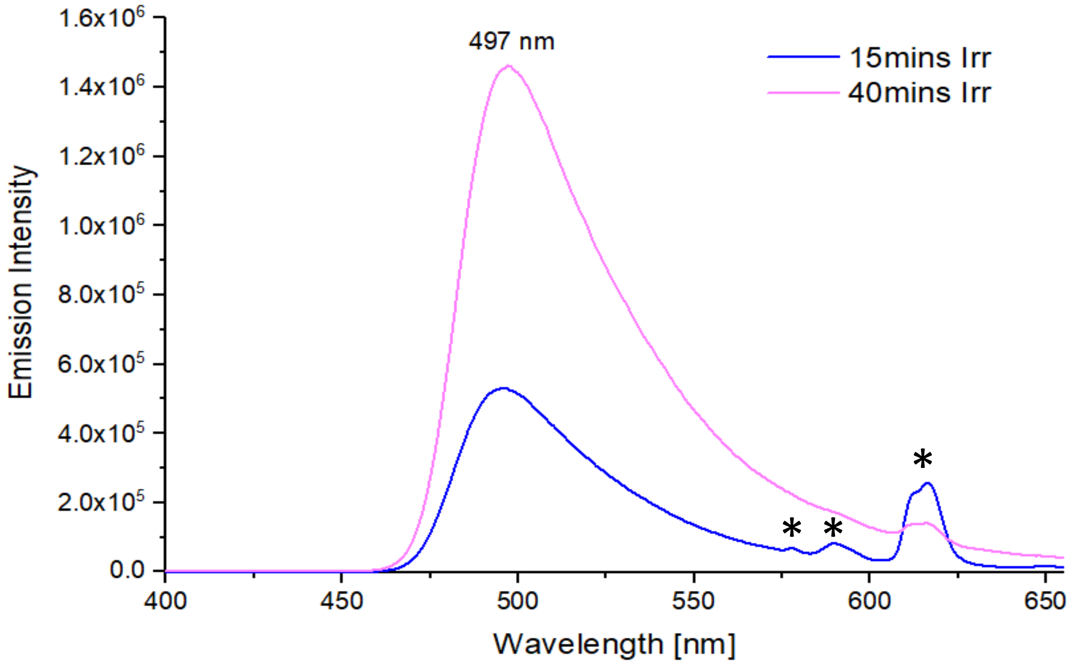

Figure S83. Emission spectra of **L7** + Eu(OTf)$_3$ (1:1 ratio, 0.3 mM) in the presence of 5.7 mM DIPEA in DMF; λ_{exc} = 360 nm; * shows Eu(III) emission.

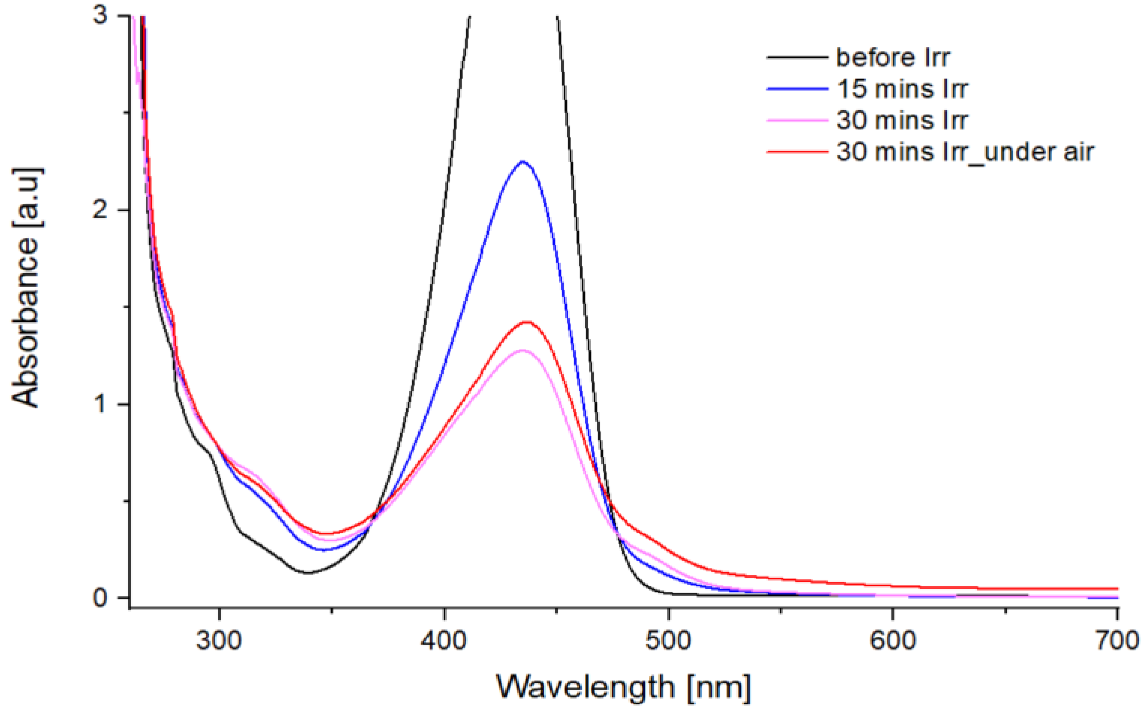

Figure S84. Absorption spectra of **L7** + $Sm(OTf)_3$ (1:1 ratio, 0.3 mM) in the presence of 5.7 mM DIPEA in DMF.

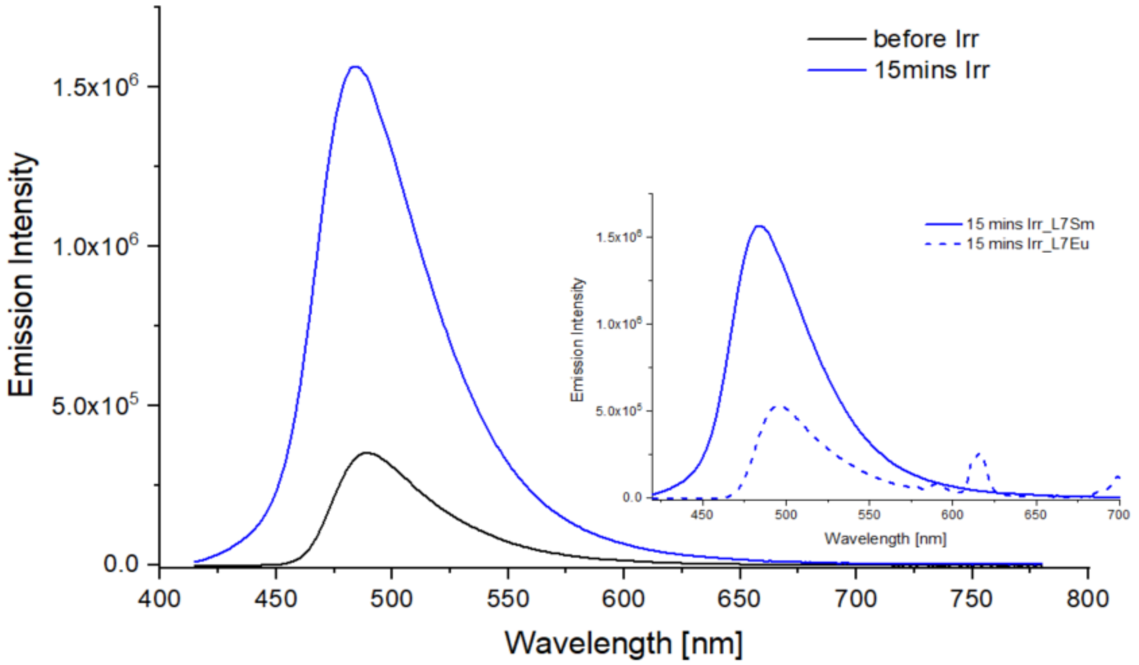

Figure S85. Emission spectra of **L7** + $Sm(OTf)_3$ (1:1 ratio, 0.3 mM) in the presence of 5.7 mM DIPEA in DMF; blue, λ_{ex} = 360 nm; inset shows the compared emission spectra of **LnL7** after 15 mins of irradiation (solid blue: Sm, dashed blue: Eu).

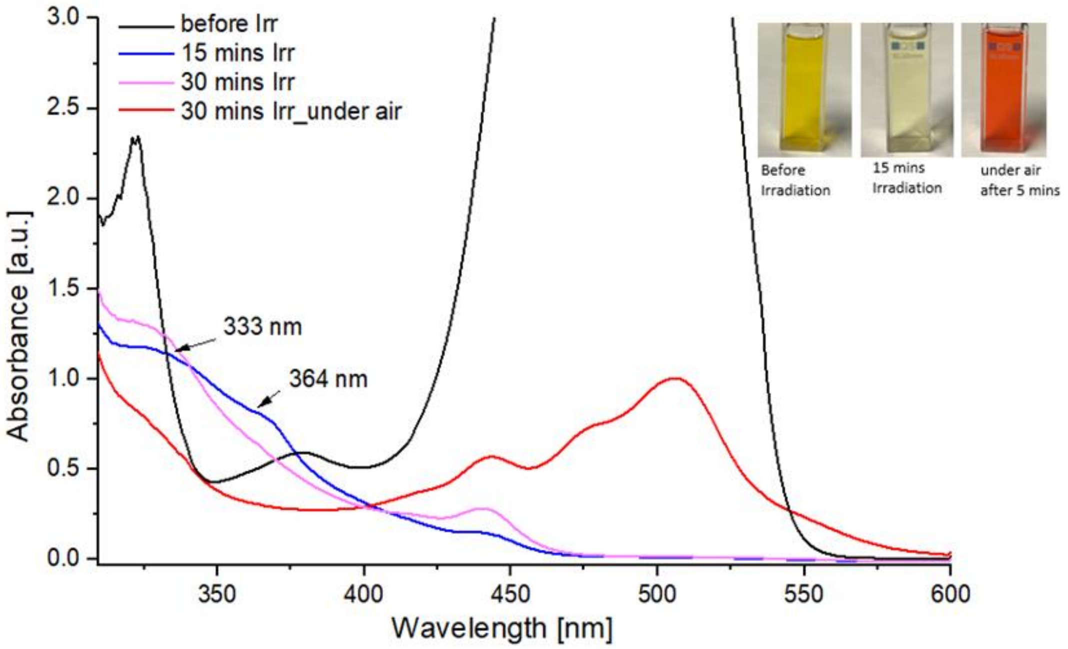

Figure S86. Absorption spectra of **L8** + Eu(OTf)$_3$ (1:1 ratio, 0.3 mM) in the presence of 5.7 mM DIPEA in DMF; pictures show the color change from before irradiation to after irradiation, and the color of the sample 5 min after exposure to air.

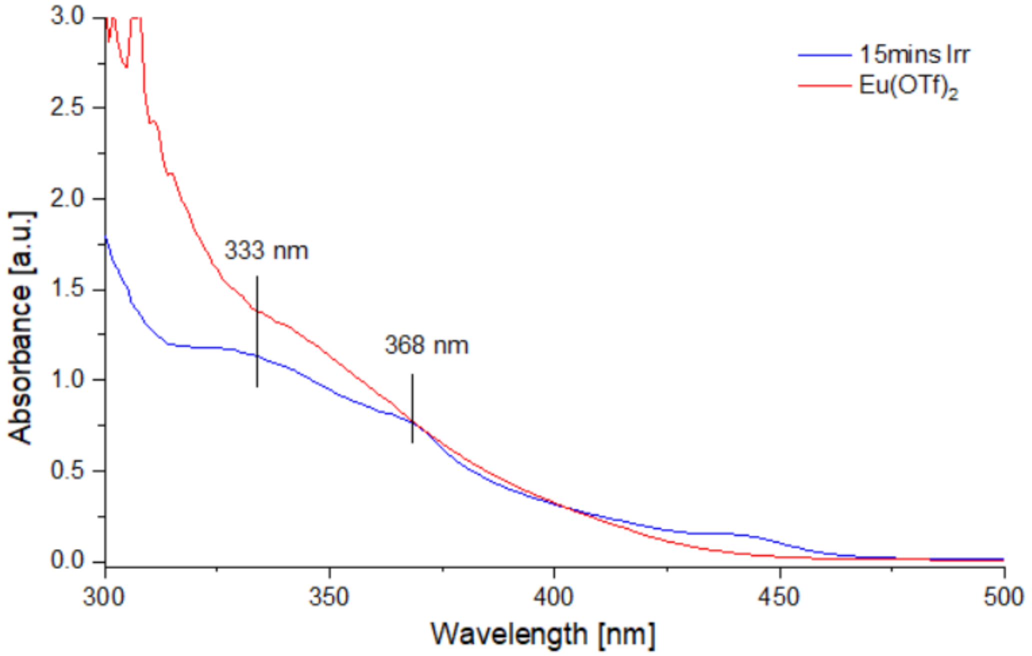

Figure S87. Absorption spectra of the irradiated mixture of **L8** and Eu(OTf)$_3$ (1:1 ratio, 0.3 mM) in the presence of 5.7 mM DIPEA in DMF (blue) compared with that of Eu(OTf)$_2$ (red).

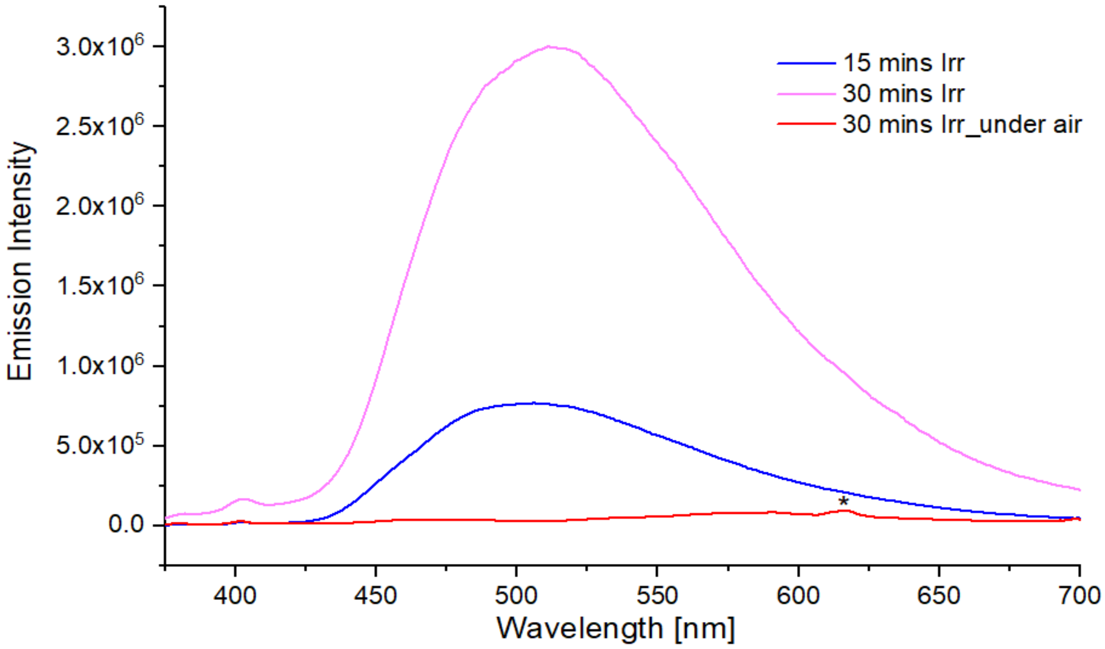

Figure S88. Emission spectra of **L8** + Eu(OTf)$_3$ (1:1 ratio, 0.3 mM) in the presence of 5.7 mM DIPEA in DMF, λ_{exc} = 360 nm; * marks Eu(III) emission.

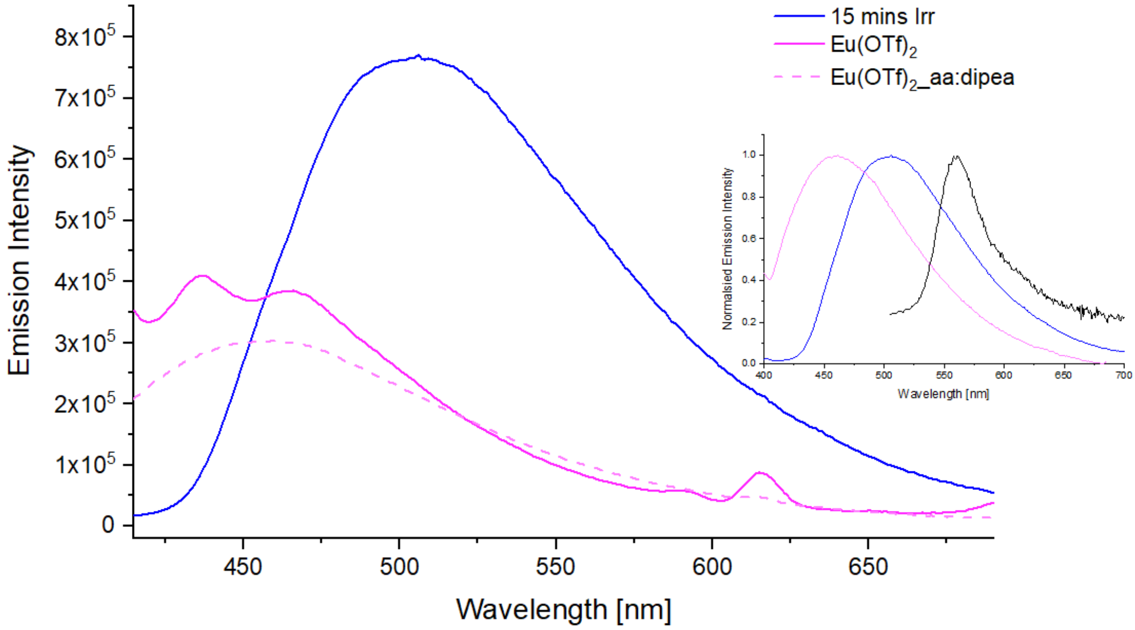

Figure S89. Comparison of the emission spectra of **L8** + Eu(OTf)$_3$ (1:1 ratio, 0.3 mM) in the presence of 5.7 mM DIPEA in DMF after 15 mins irradiation (blue) with Eu(OTf)$_2$ (pink solid line) and Eu(OTf)$_2$ in the presence of acetic acid and DIPEA (pink dashed line); inset showed the normalized emission spectra of **L8Eu** after 15 mins irradiation (λ_{ex} = 360 nm), Eu(OTf)$_2$ (λ_{ex} = 360 nm) and **L8**:Eu(OTf)$_3$ (black), λ_{ex} = 490 nm.

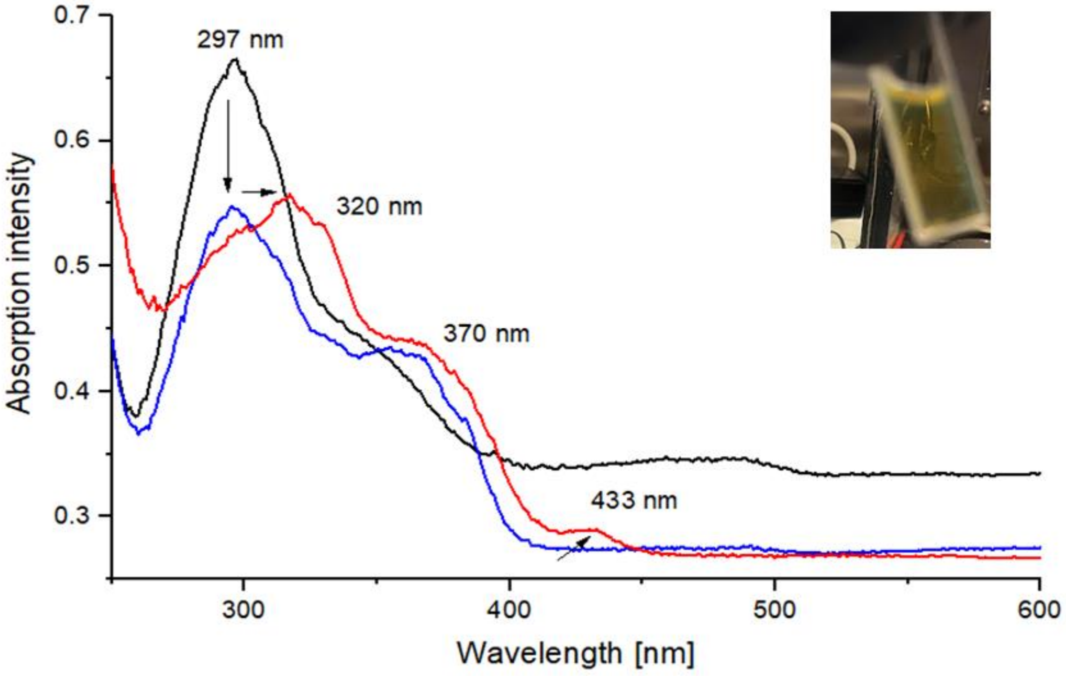

Figure S90. UV-Vis absorption spectrum of $Eu(OTf)_3$ + acetic acid (1.33 mM each) before electrolysis (black), after 15 mins (blue) and 25 mins (red) of an applied potential of –0.35V in MeCN; reference electrode, $Ag/AgNO_3$; working electrode and counter electrode, Carbon mesh. The inset shows the pale yellow solution after the application of the potential.

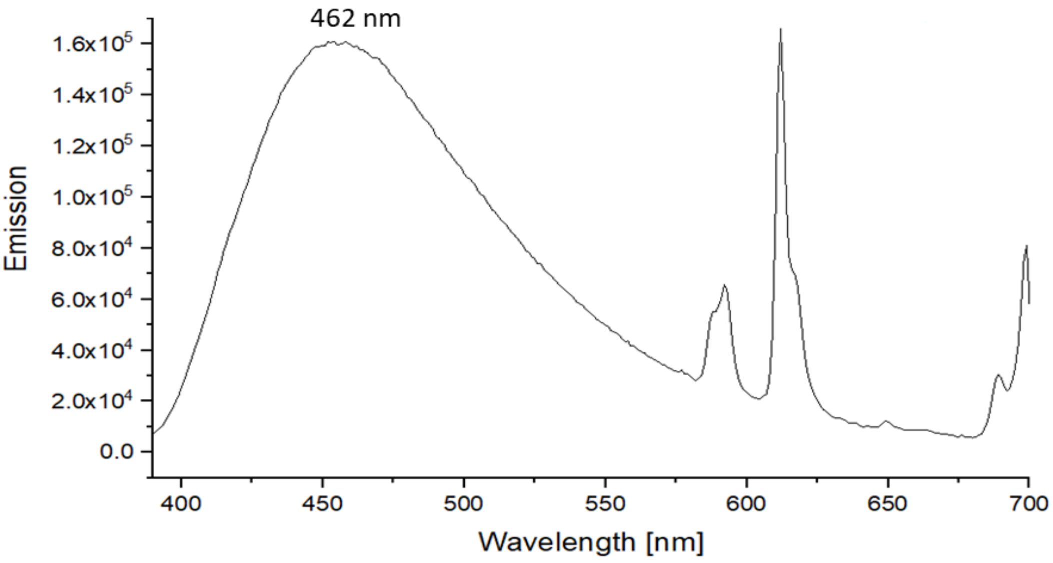

Figure S91. Steady-state emission spectra of a solution of $Eu(OTf)_3$ + acetic acid (1.33 mM); after 25 mins of applied potential of –0.35V in MeCN, λ_{ex} = 370 nm.

3. EPR spectroscopy

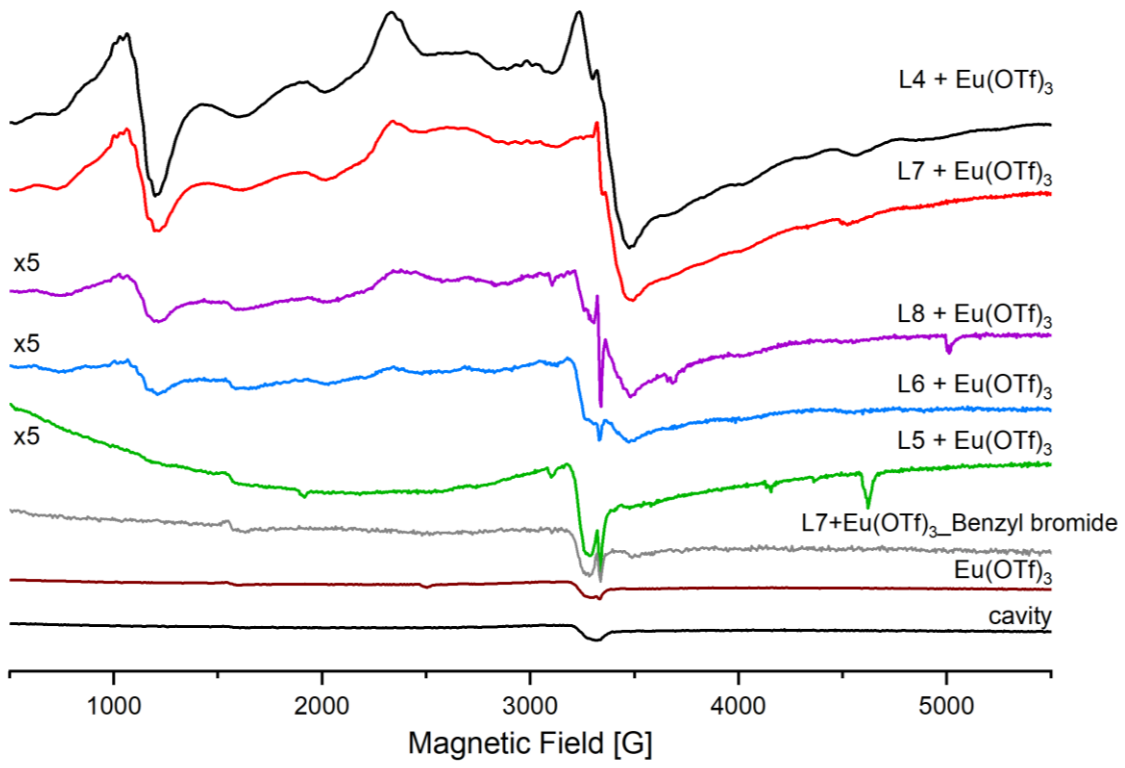

Figure S92. EPR-signals from mixtures of $Eu(OTf)_3$ and **L4–L8** ([**L**] = [$Eu(OTf)_3$] = 1 mM DMF) after irradiation for 30–60 mins with blue LED. Spectra of **L5** + $Eu(OTf)_3$, **L6** + $Eu(OTf)_3$, and **L8** + $Eu(OTf)_3$ are multiplied by 5 for clarity. Temp: 10 K, microwave power: 2 mW, modulation amplitude: 19.5 G.

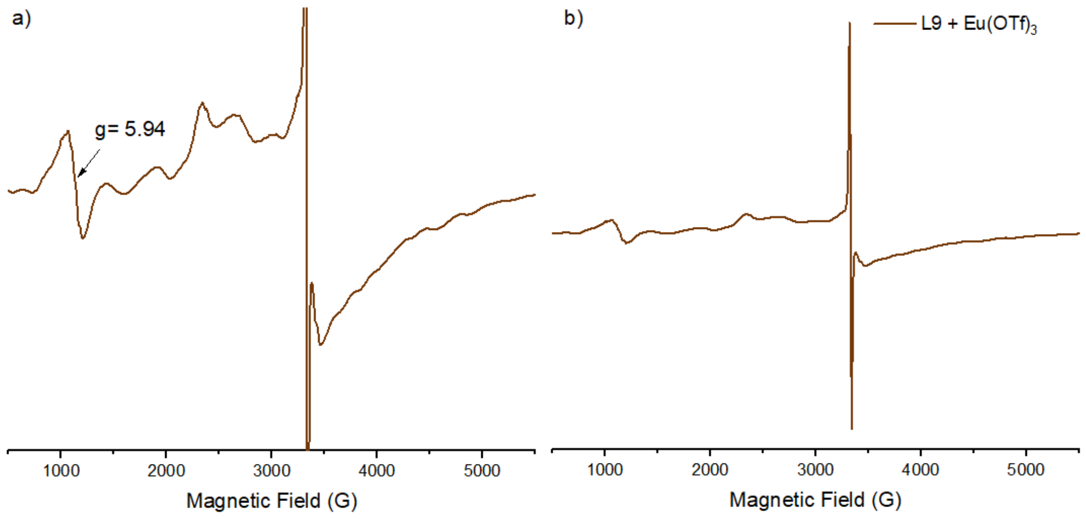

Figure S93. a) EPR-signal from a mixture of **L9** and $Eu(OTf)_3$ ([**L9**] = [$Eu(OTf)_3$] = 1 mM DMF) after irradiation for 30 mins with blue LED (organic radical cut off). b) Full EPR-spectrum with visible organic radical. Temp: 10 K, microwave power: 2 mW, modulation amplitude: 19.5 G.

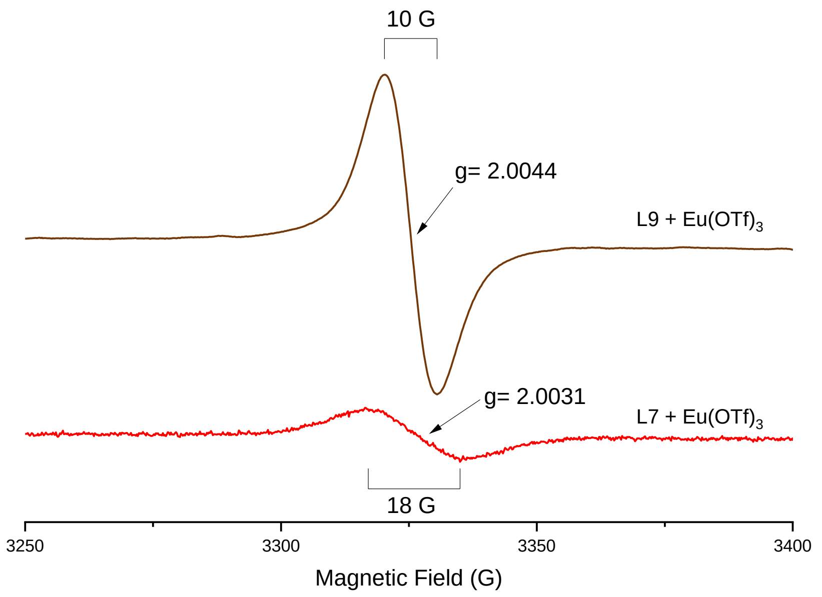

Figure S94. Organic radicals in EPR samples of **L9** and $Eu(OTf)_3$ (brown), and **L7** and $Eu(OTf)_3$ (red) recorded after irradiation for 30 mins with blue LED ([**L**] = [$Eu(OTf)_3$] = 1 mM DMF). Temp: 10 K, microwave power: 2 µW, modulation amplitude: 3 G.

4. Electrochemistry

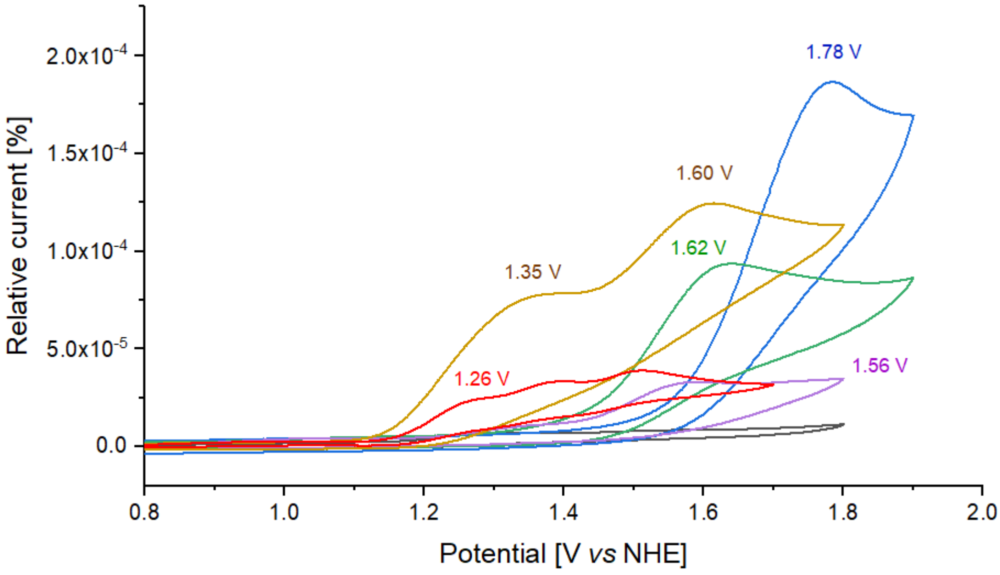

Figure S95. Cyclic voltammograms of **L** (**L4**: black; **L5**: green; **L6**: blue; **L7**: red; **L8**: purple, **L9**: mustard) in DMF (0.1 M $TBAPF_6$). [**L**] = 5 mM in DMF; reference electrode, Ag/AgCl; working electrode, GC electrode; counter electrode, Pt wire; scan rate, 0.1 V/s.

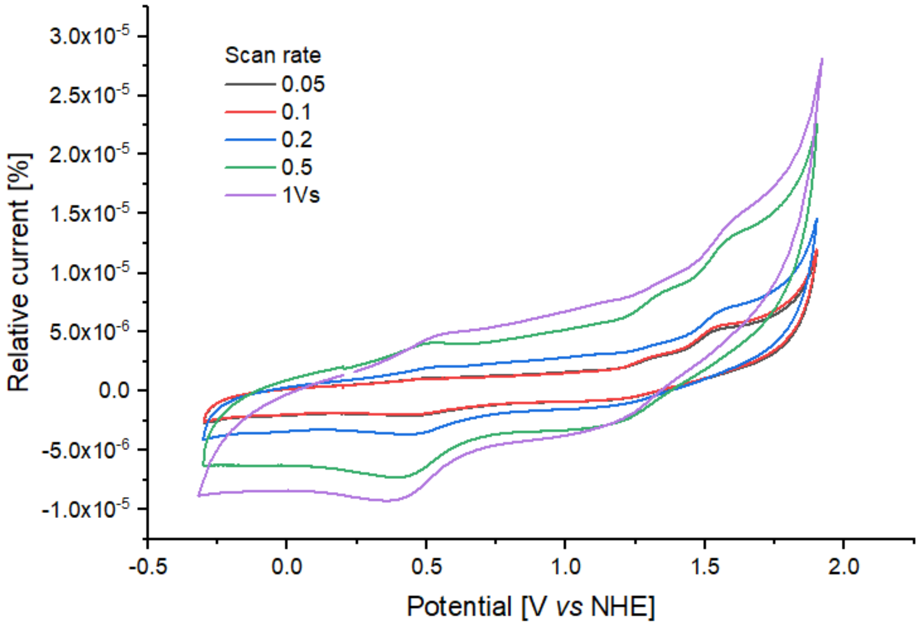

Figure S96. Cyclic voltammogram of **L4** at various scan rates in DMF (0.1 M $TBAPF_6$). [**L4**] = 5 mM; reference electrode, Ag/AgCl; working electrode, GC electrode; counter electrode, Pt wire.

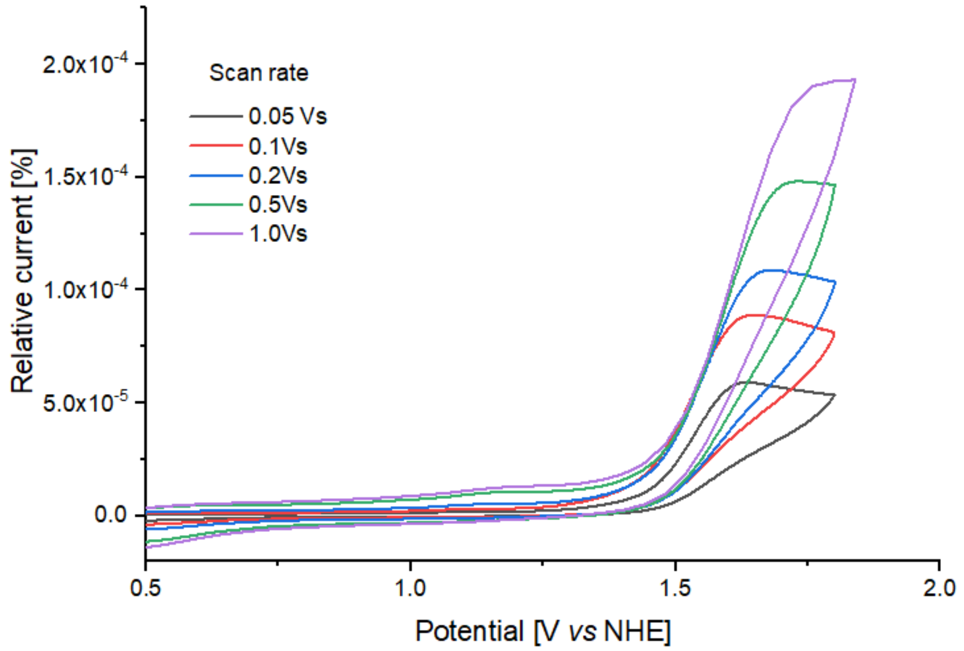

Figure S97. Cyclic voltammograms of **L5** at various scan rates in DMF (0.1 M $TBAPF_6$). [**L5**] = 5 mM; reference electrode, Ag/AgCl; working electrode, GC electrode; counter electrode, Pt wire.

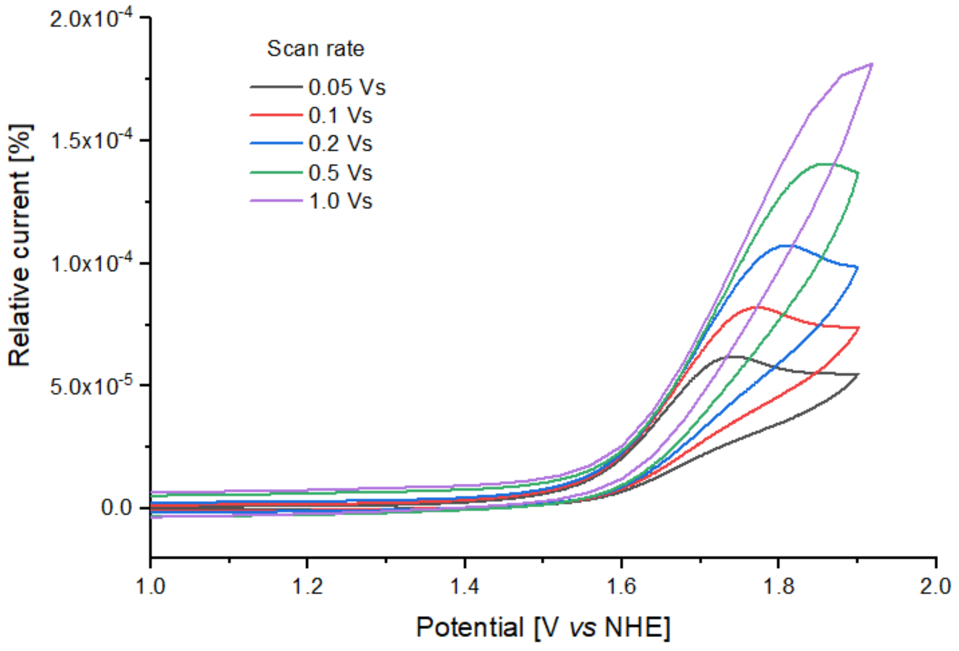

Figure S98. Cyclic voltammograms of **L6** at various scan rates in DMF (0.1 M $TBAPF_6$). [**L6**] = 5 mM; reference electrode, Ag/AgCl; working electrode, GC electrode; counter electrode, Pt wire.

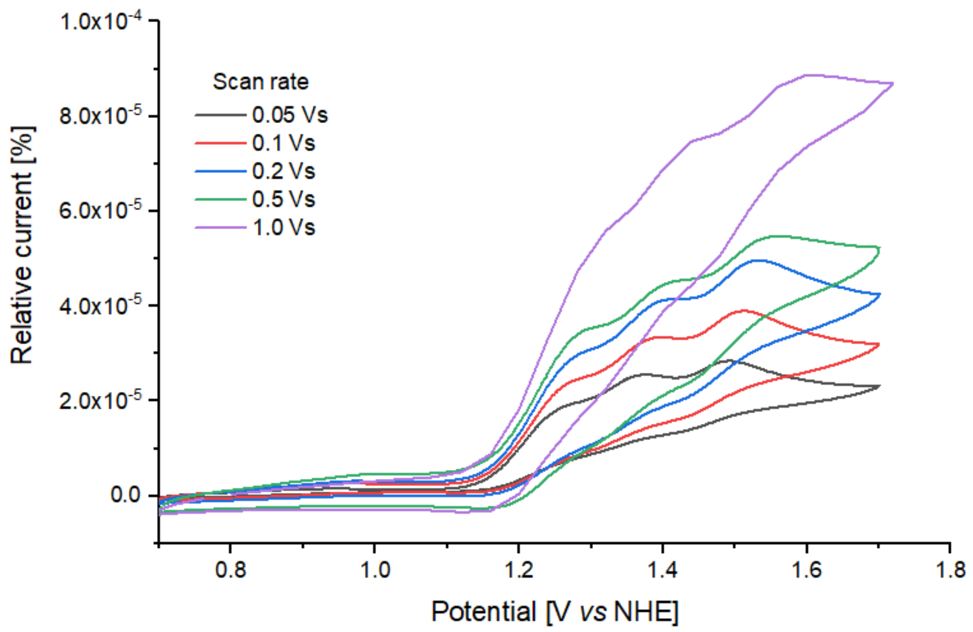

Figure S99. Cyclic voltammogram of **L7** at various scan rates in DMF (0.1 M $TBAPF_6$). [**L7**] = 5 mM; reference electrode, Ag/AgCl; working electrode, GC electrode; counter electrode, Pt wire.

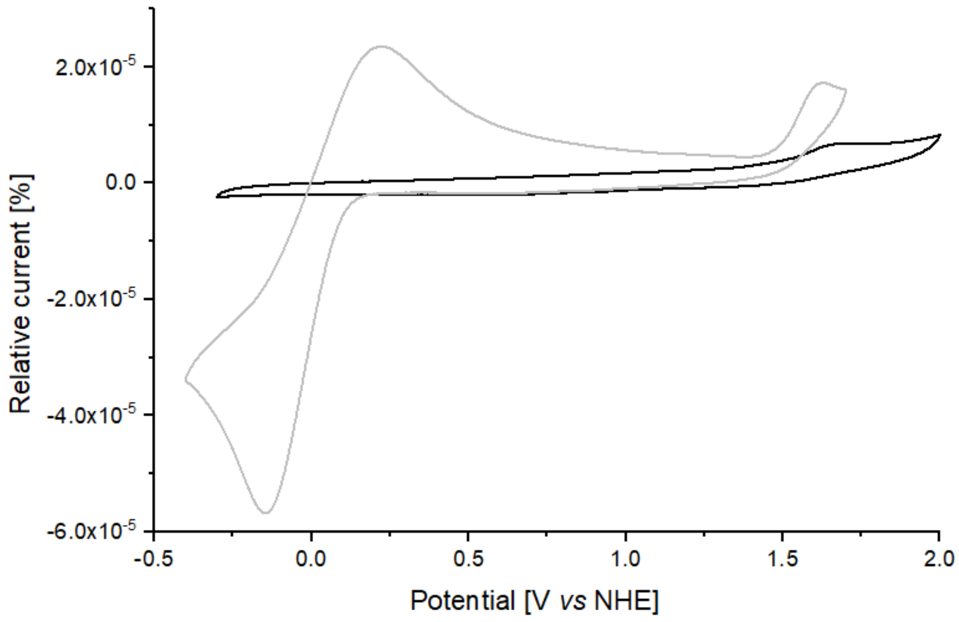

Figure S100. Cyclic voltammograms of **L4** (black), and of **L4** and $Eu(OTf)_3$ (grey). [**L4**] = 5 mM in MeCN; reference electrode, Ag/AgCl; working electrode, GC electrode; counter electrode, Pt wire; scan rate, 0.1 V/s.

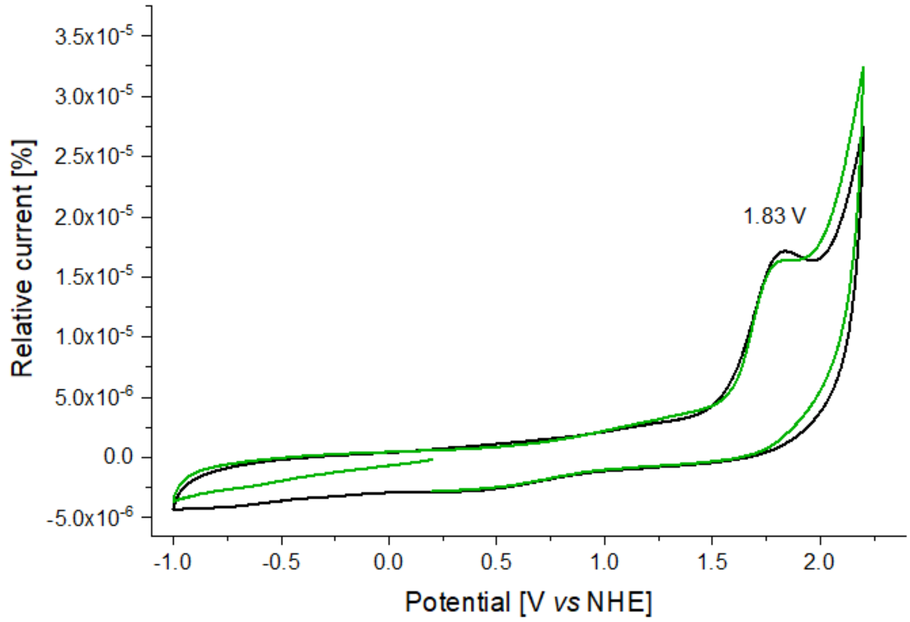

Figure S101. Cyclic voltammograms of **L5** (black), and of **L5** and Eu(OTf)$_3$ (green) [**L5**] = 5 mM in MeCN; reference electrode, Ag/AgCl; working electrode, GC electrode; counter electrode, Pt wire; scan rate, 0.1 V/s.

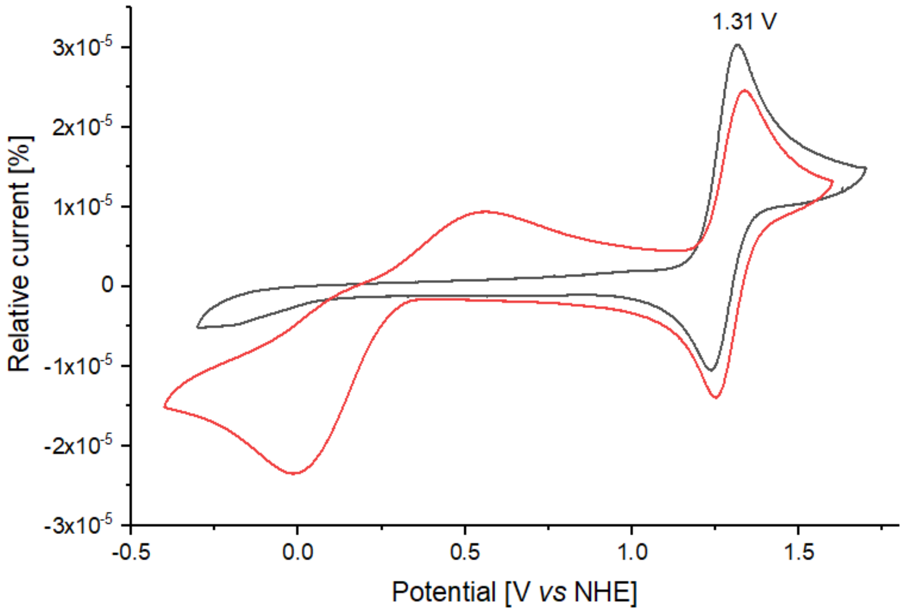

Figure S102. Cyclic voltammograms of **L7** (black), and of **L7** and Eu(OTf)$_3$ (red). [**L7**] = 5 mM in MeCN; reference electrode, Ag/AgCl; working electrode, GC electrode; counter electrode, Pt wire; scan rate, 0.1 V/s.

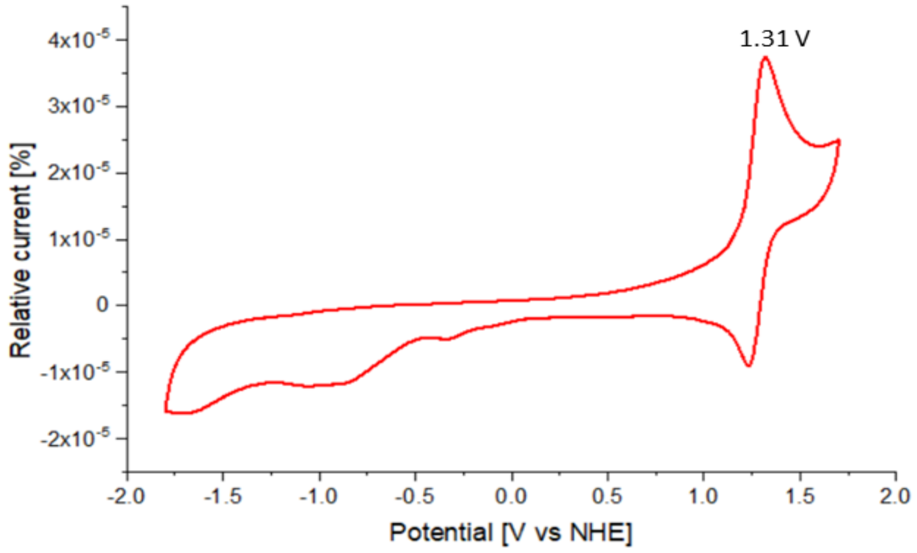

Figure S103. Cyclic voltammogram of **L7** +Sm(OTf)$_3$, 5 mM in MeCN; reference electrode, Ag/AgCl; working electrode, GC electrode; counter electrode, Pt wire; scan rate, 0.1 V/s.

5. PeT driving force calculations

The driving force for PeT from the excited ligands (**L**) was calculated according to equation S2.[10]

$$\Delta G(eT) = \left(E_{ox}^{L} - E_{red}^{Eu}\right) - E_{s}^{L} - \frac{{e_0}^2}{\varepsilon r} \qquad \text{(S2)}$$

$\Delta G(eT)$ is the free energy of electron transfer, E_{ox}^{L} the ground state oxidation potential of **L**, E_{red}^{Eu} the reduction potential of Eu(III) in a mixture of **L** and Eu(OTf)$_3$, E_{s}^{L} the singlet excited state of **L** taken from the emission spectra of **L** (Table S1), and $\frac{{e_0}^2}{\varepsilon r}$ the attraction between the radical ion pair (~0.15 eV for an exciplex, here taken as 0).[10]

$$\Delta G(eV) = \left(E_{ox}^{L5} - E_{red}^{Eu}\right) - E_{s}^{L5} - \frac{{e_0}^2}{\varepsilon r} = \left(1.62 - (-0.596)\right) - 2.66\ eV - 0.15\ eV = -1.45\ eV$$

Table S1. Data for driving force calculations and $\Delta G(eT)$ for **L4**–**L9**.

L	E_{ox}^{L} (V *vs* NHE) [a]	E_{S}^{L} (eV)	E_{T}^{L} (eV)	E_{red}^{Eu} (V *vs* NHE)	ΔG_{eT}^{S} [eV] [c]	ΔG_{eT}^{T} [eV] [c]
**L1**	1.10 (vs Fc/Fc$^+$) [b]	2.98	2.37 [b]	–1.39 (vs Fc/Fc$^+$) [b]	–0.83	–0.03
**L2**	1.10 (vs Fc/Fc$^+$) [b]	2.98	2.37 [b]	–1.27 (vs Fc/Fc$^+$) [b]	–0.92	–0.15
**L4**	1.27	2.97	2.26	–0.48	–1.37	–0.51
**L5**	1.62	2.66	2.28	–0.596	–0.594	–0.06
**L6**	1.78	2.47	2.16	–0.465	–0.375	–0.09
**L7**	1.26	2.52	2.08	–0.458	–0.952	–0.36
**L8**	1.56	2.27	1.96	–0.757	–0.103	0.36
**L9**	1.35	1.87	2.06	–0.614	–0.056	–0.10

[a] As the dye oxidations were irreversible under the experimental conditions the oxidation potential is used. [b] Data from Reference [1]. [c] ΔG_{eT}^{S} and ΔG_{eT}^{T} refer to PeT from the singlet and triplet states to Eu(III), respectively.

6. Photocatalysis

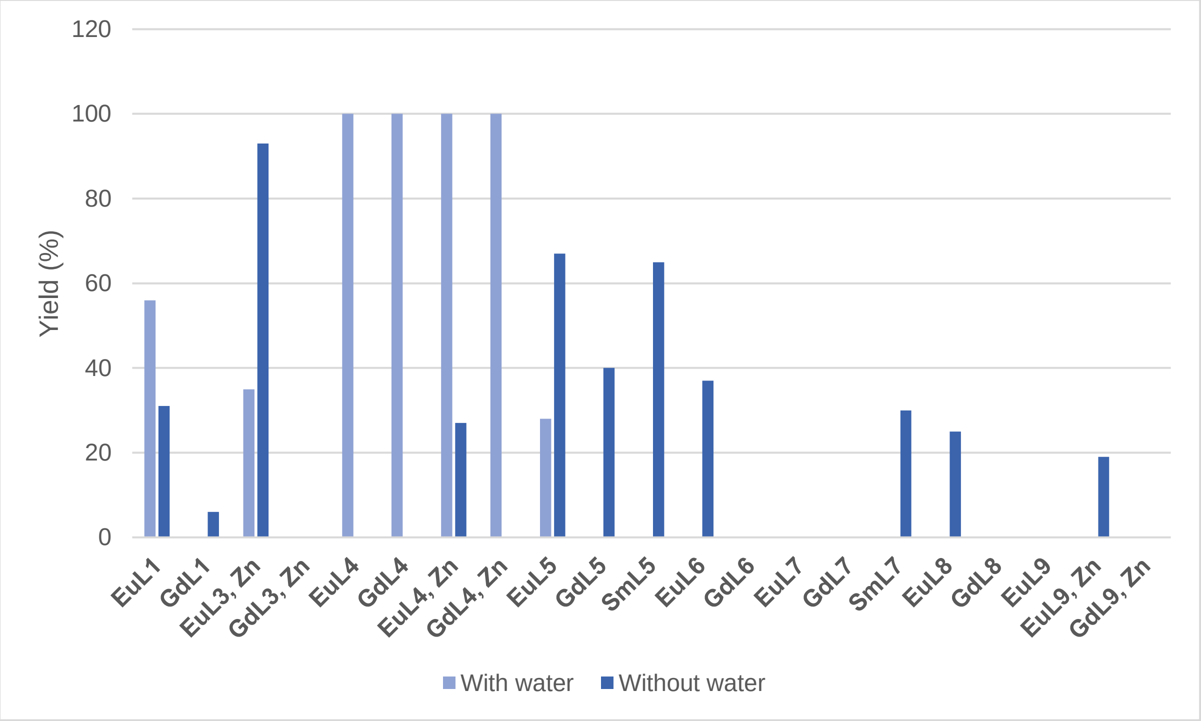

Figure S104. Yield of photocatalysis experiments. [a] Reactions were carried out using **EuL1–L9** and **GdL1–L9** in the absence of water (dark blue) and with added water (light blue bars) as described in the **Materials and methods** section. Reactions using Zn as the sacrificial reductant are marked as such.

7. IR spectra

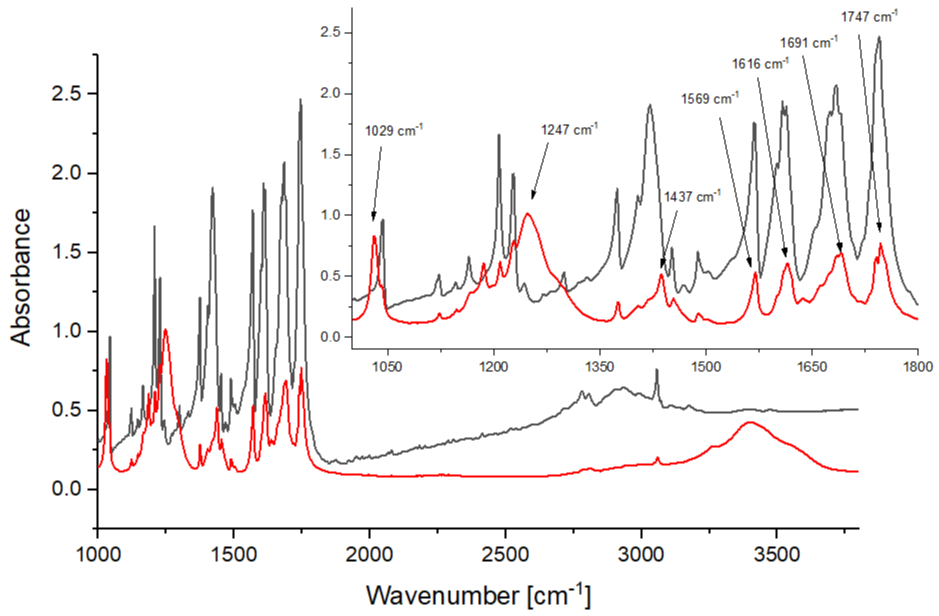

Figure S105. IR spectra of **L4** (black), and of **L4** and Eu(OTf)$_3$ (red, 1:1 ratio). The strong O-H stretches may be due to the water content of the lanthanide salt.

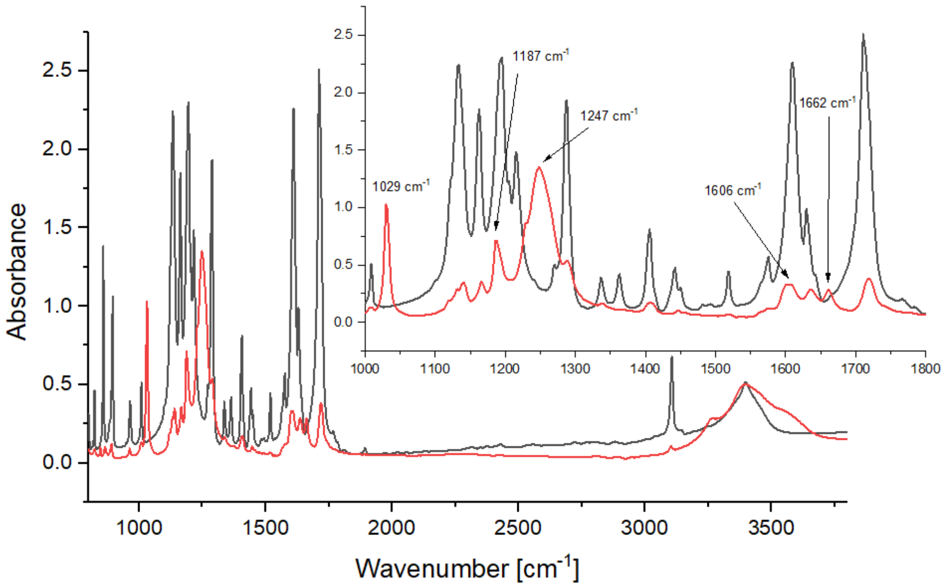

Figure S106. IR spectra of **L6** (black), and of **L6** and Eu(OTf)$_3$ (red, 1:1 ratio). The strong O-H stretches may be due to the water content of the lanthanide salt.

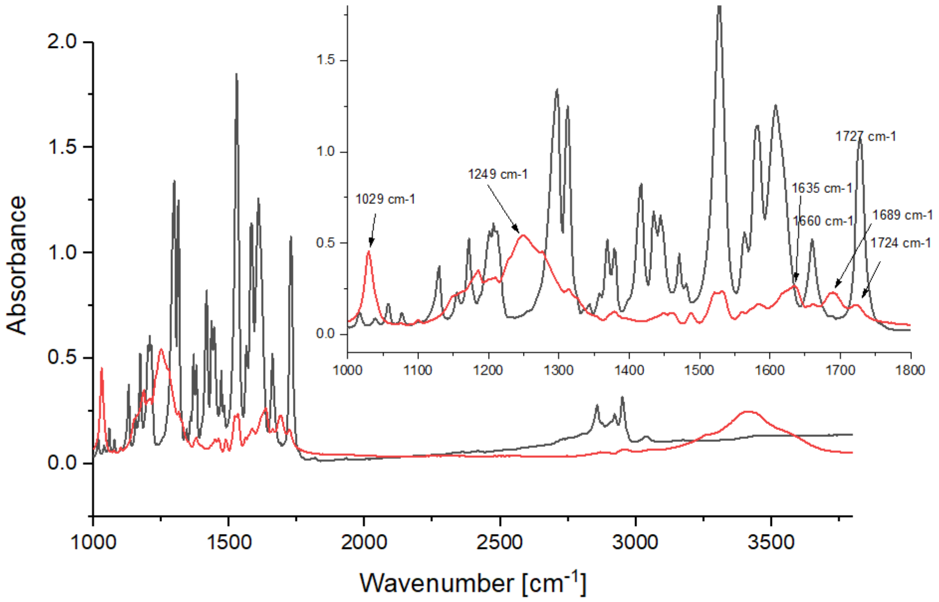

Figure S107. IR spectra of **L7** (black), and of **L7** and Eu(OTf)$_3$ (red, 1:1 ratio). The strong O-H stretches may be due to the water content of the lanthanide salt.

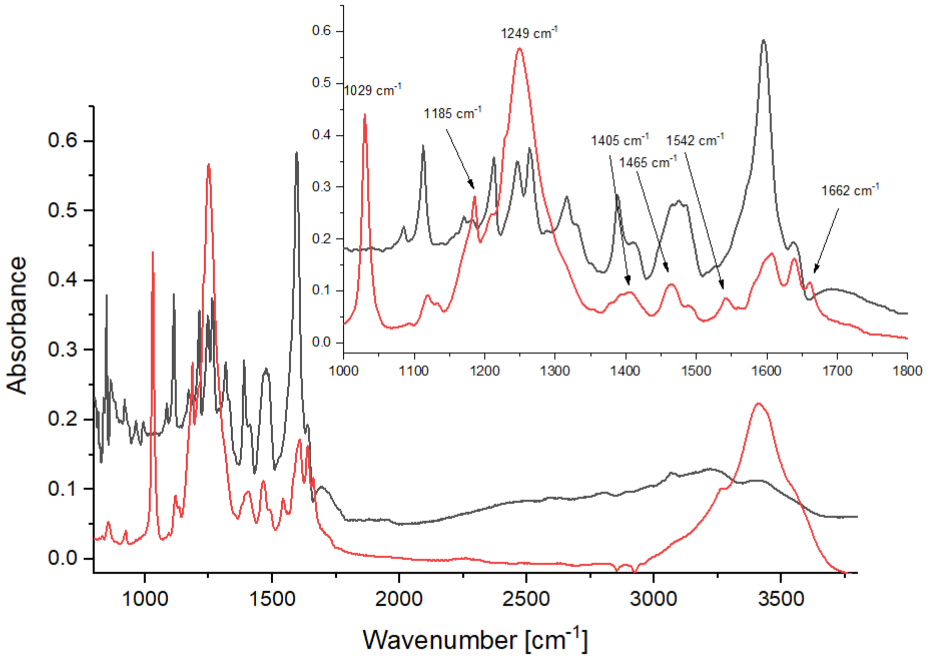

Figure S108. IR spectra of **L8** (black), and of **L8** and Eu(OTf)$_3$ (red, 1:1 ratio). The strong O-H stretches may be due to the water content of the lanthanide salt.

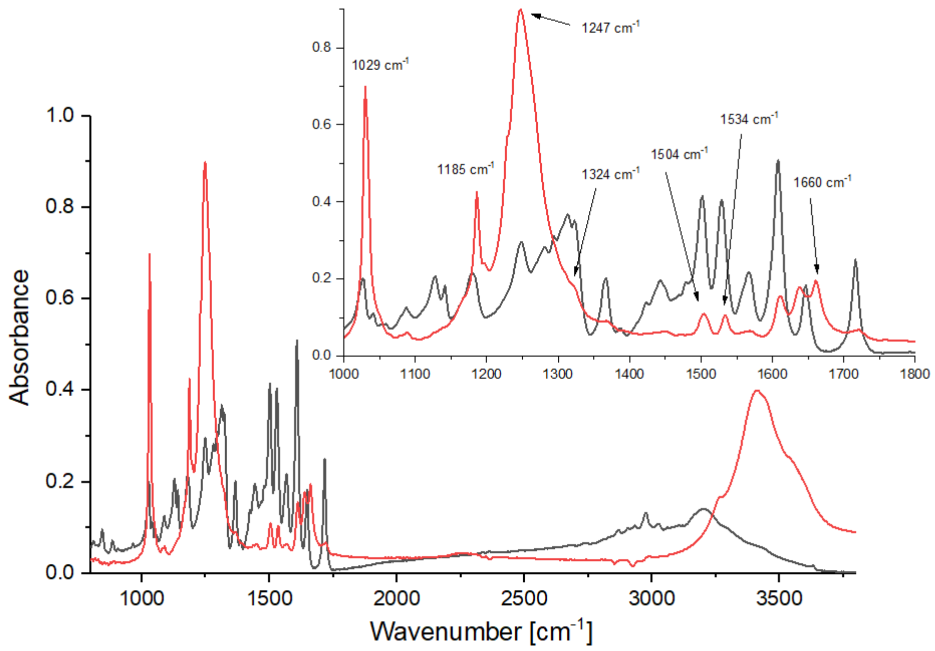

Figure S109. IR spectra of **L9** (black), and of **L9** and Eu(OTf)$_3$ (red, 1:1 ratio). The strong O-H stretches may be due to the water content of the lanthanide salt.

Table S2. Tabulated FT-IR data of **L4–L9**, and of 1:1 mixtures of **L** with Eu(OTf)$_3$.

Compound	Wavenumber (cm^{-1})
**L4**	3056, 2921, 2780, 1745, 1685, 1608, 1567, 1421, 1374, 1228, 1207, 1164, 1043
**L4 + Eu(III)**	3401, 3060, 2984, 1747, 1691, 1615, 1569, 1436, 1375, 1247, 1185, 1029
**L5**	3351, 1662, 1637, 1249, 1185, 1029
**L5 + Eu(III)**	3370, 1697, 1660, 1635, 1245, 1185, 1123, 1029
**L6**	3395, 3106, 1710, 1610, 1517, 1442, 1405, 1288, 1195, 1214, 1162, 1133, 1008
**L6 + Eu(III)**	3550, 3340, 1718, 1662, 1635, 1604, 1405, 1288, 1247, 1185, 1166, 1139, 1029
**L7**	2950, 2921, 2857, 1727, 1660, 1608, 1583, 1527, 1434, 1417, 1369, 1313, 1297, 1207, 1172, 1130
**L7 + Eu(III)**	3415, 1724, 1689, 1635, 1583, 1532, 1521, 1486, 1450, 1378, 1313, 1249, 1209, 1185, 1029
**L8**	3415, 3230, 1691, 1637, 1594, 1475, 1388, 1317, 1263, 1245, 1212, 1112
**L8 + Eu(III)**	3413, 1646, 1606, 1540, 1465, 1403, 1251, 1185, 1118, 1029
**L9**	3201, 2977, 1716, 1646, 1608, 1565, 1529, 1502, 1444, 1367, 1311, 1247, 1180, 1128, 1025
**L9 + Eu(III)**	3413, 1660, 1637, 1610, 1534, 1504, 1319, 1247, 1228, 1185, 1029

8. GC-MS traces

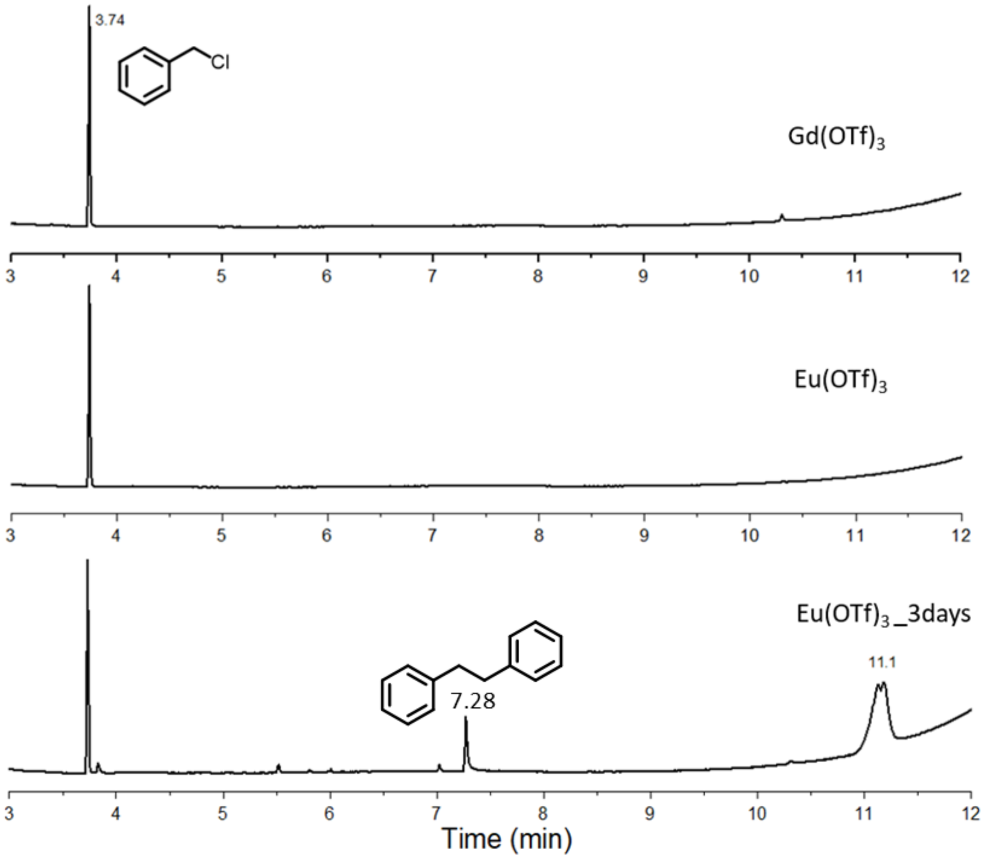

Figure S110. GC chromatograms of the reaction mixtures of **L4** with DIPEA:LiCl in DMF:H_2O (4:1).

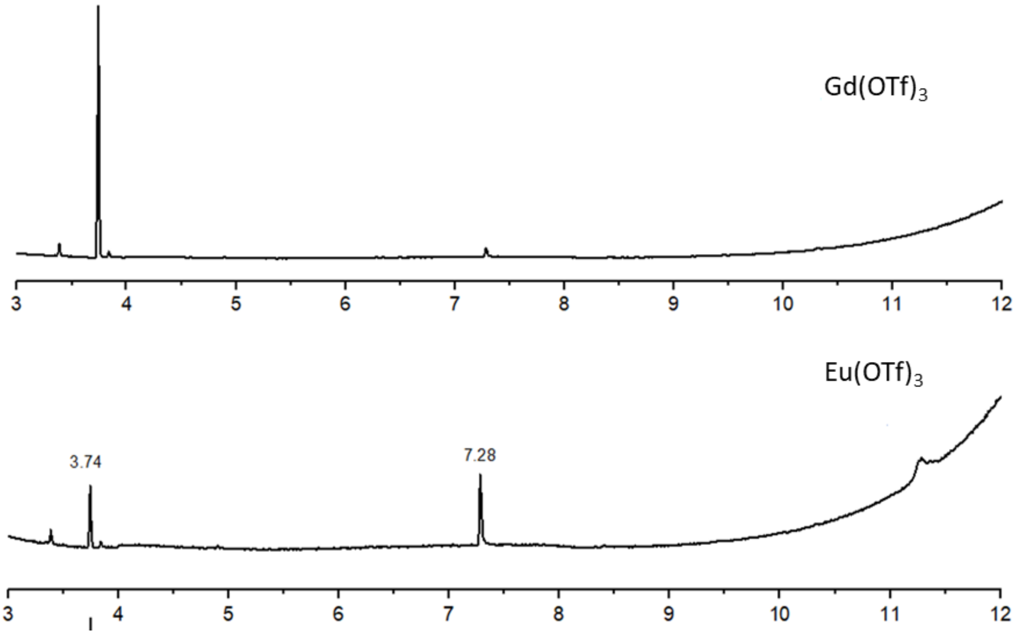

Figure S111. GC chromatograms of the reaction mixtures of **L4** with Zn in DMF.

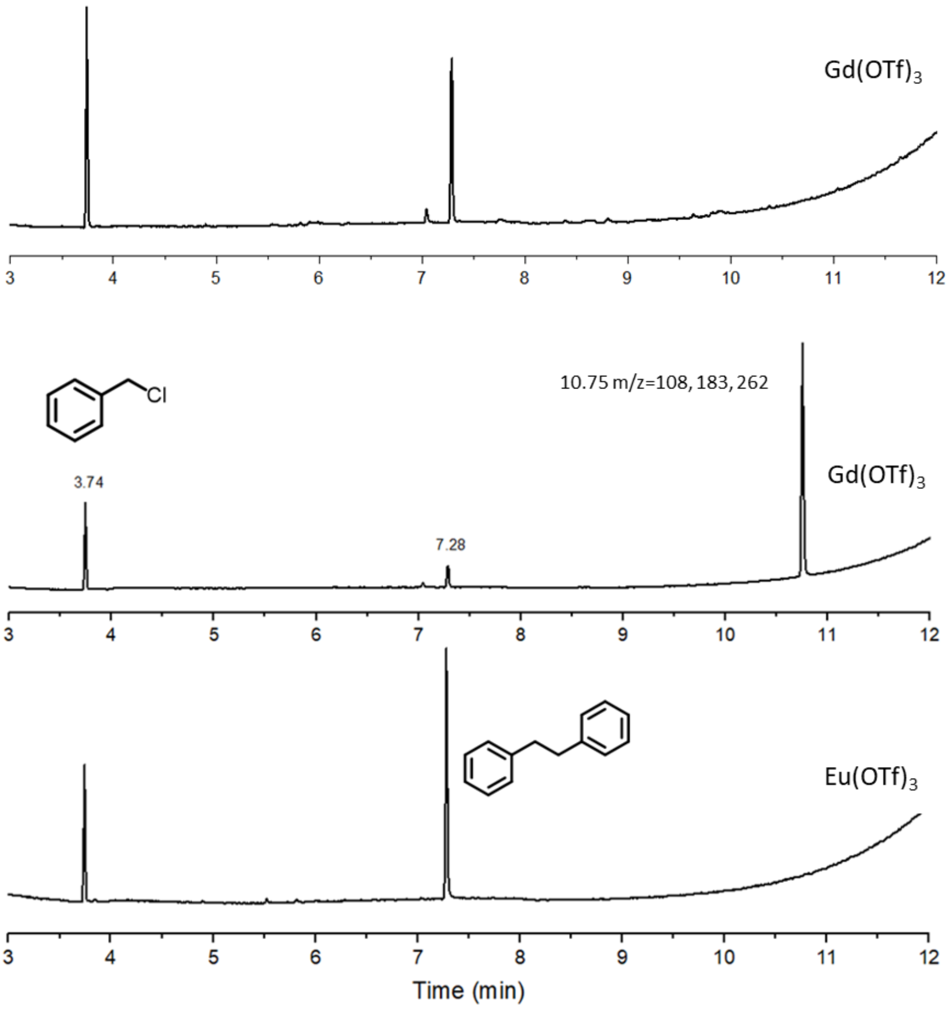

Figure S112. GC chromatograms of the reaction mixtures of **L5** with DIPEA:LiCl in DMF.

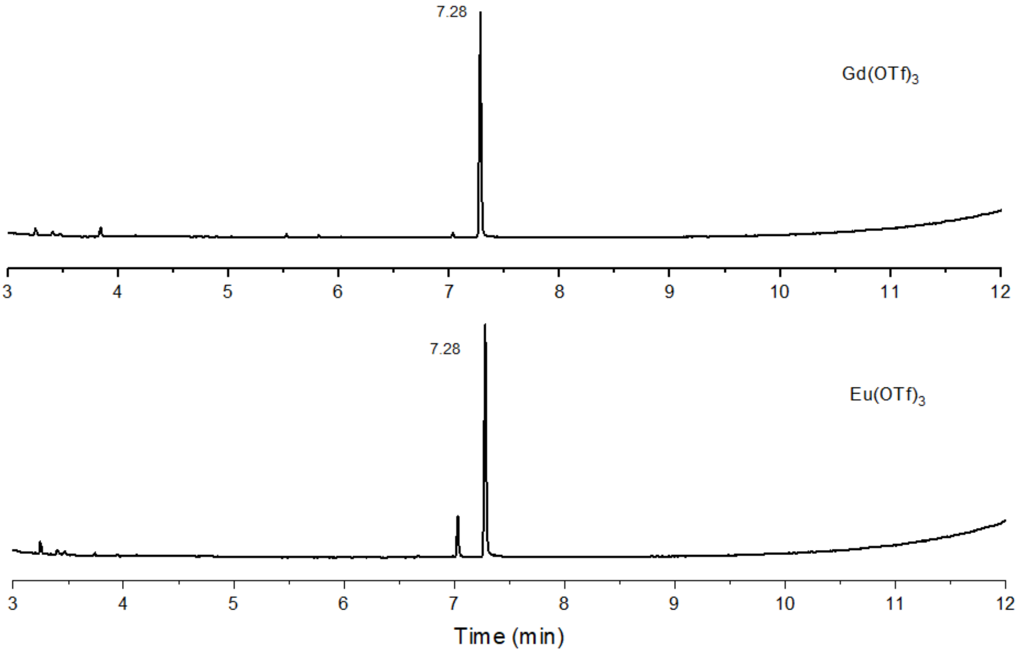

Figure S113. GC chromatograms of the reaction mixtures of **L5** with DIPEA:LiCl in DMF:H_2O (4:1).

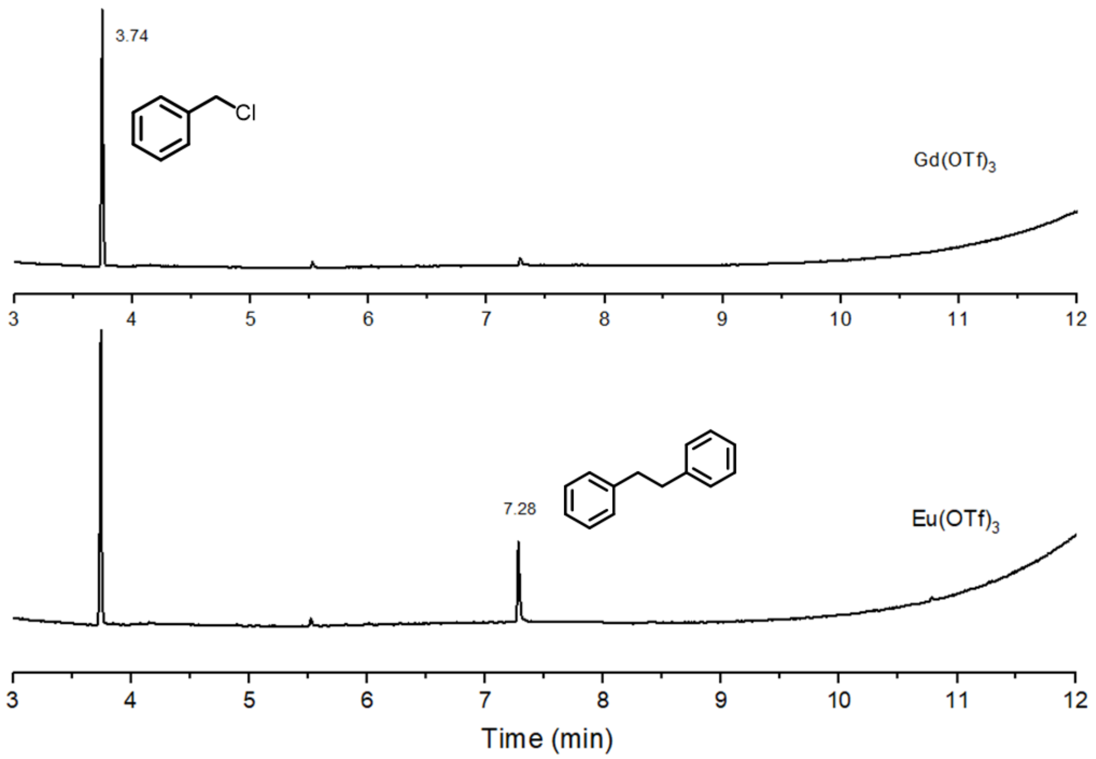

Figure S114. GC chromatograms of the reaction mixtures of **L6** with DIPEA:LiCl in DMF.

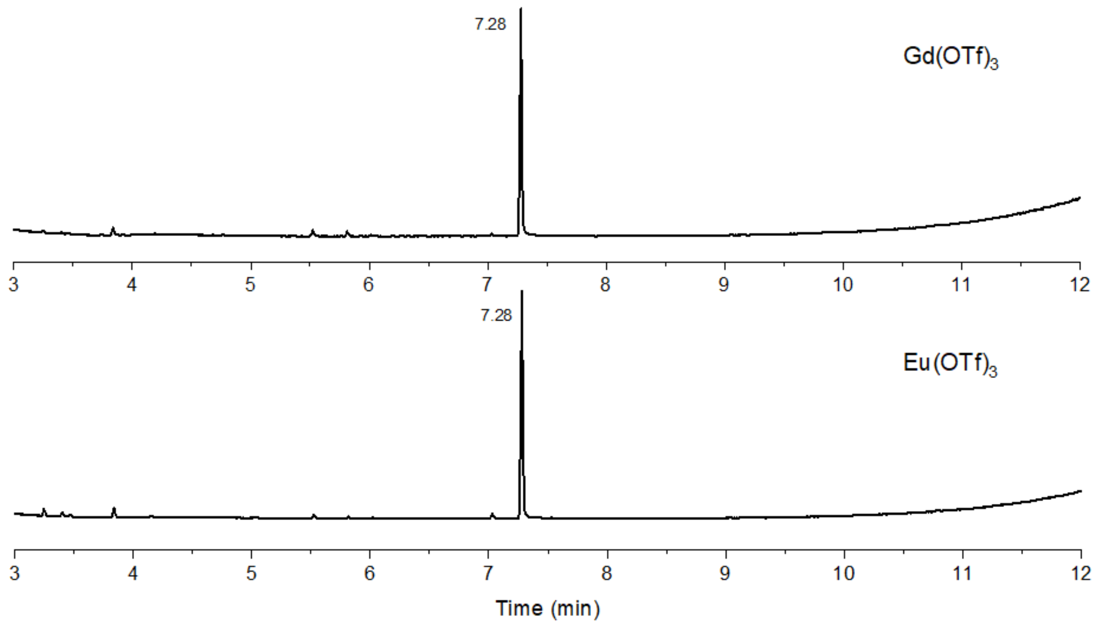

Figure S115. GC chromatograms of the reaction mixtures of **L6** with DIPEA:LiCl in DMF:H_2O (4:1).

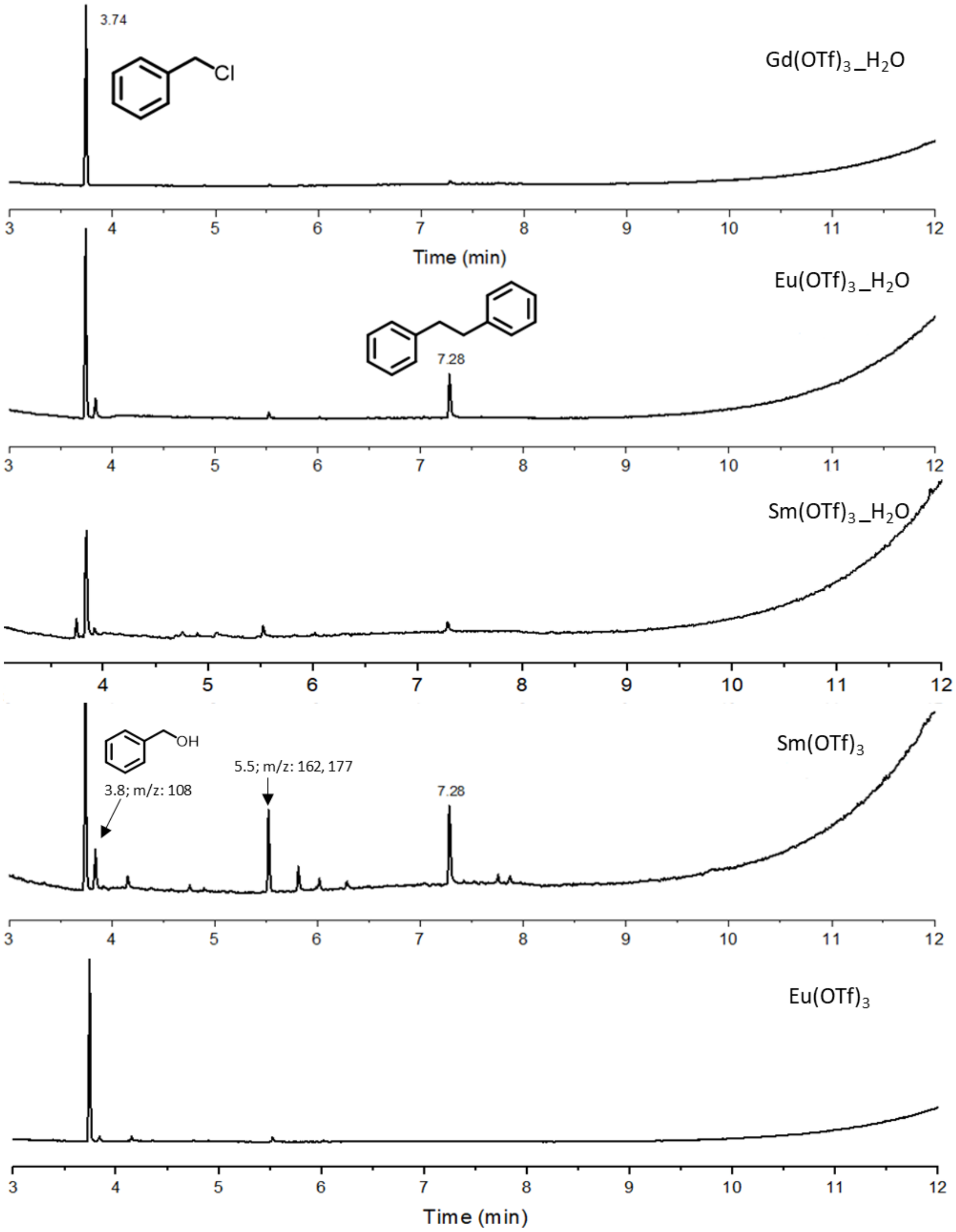

Figure S116. GC chromatograms of the reaction mixtures of **L7** with DIPEA:LiCl.

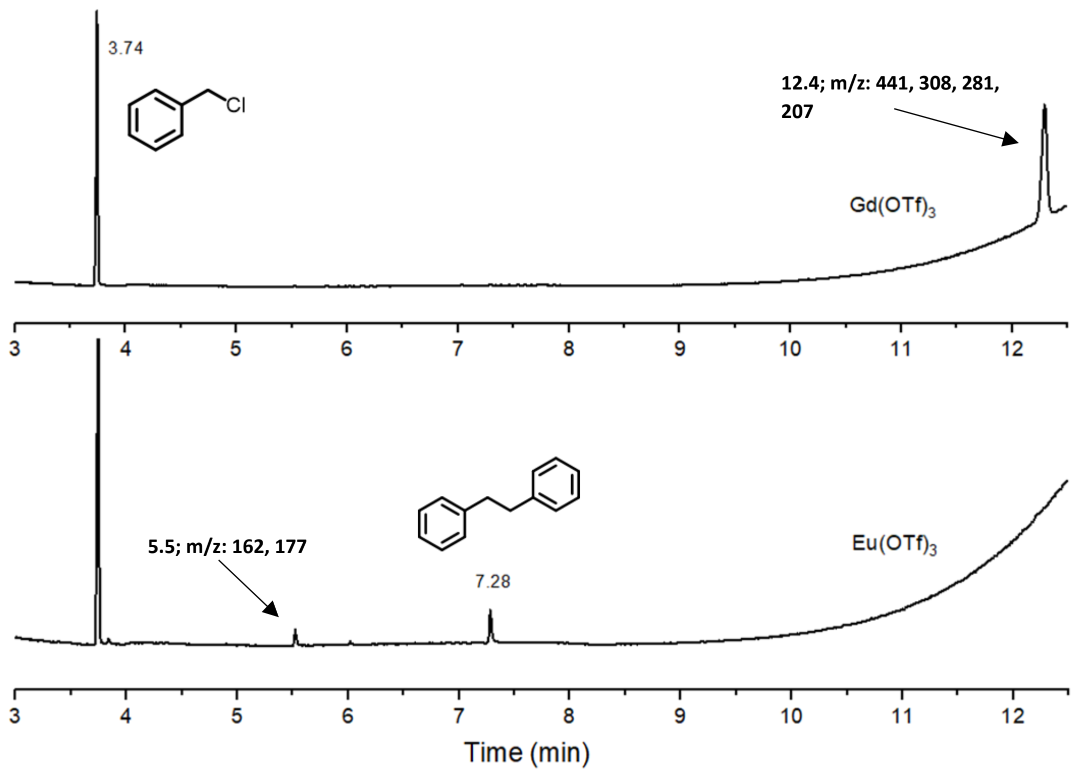

Figure S117. GC chromatograms of the reaction mixtures of **L8** with DIPEA:LiCl in DMF.

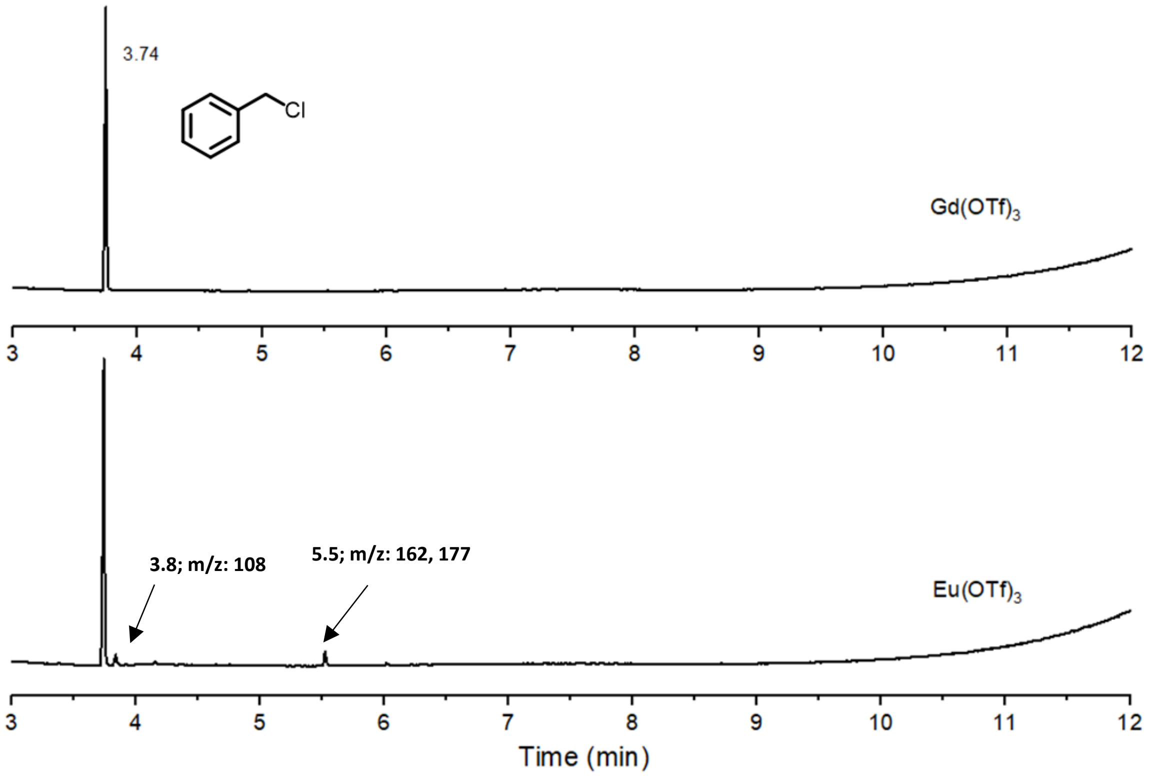

Figure S118. GC chromatograms of the reaction mixtures with **L9** with DIPEA:LiCl in DMF.

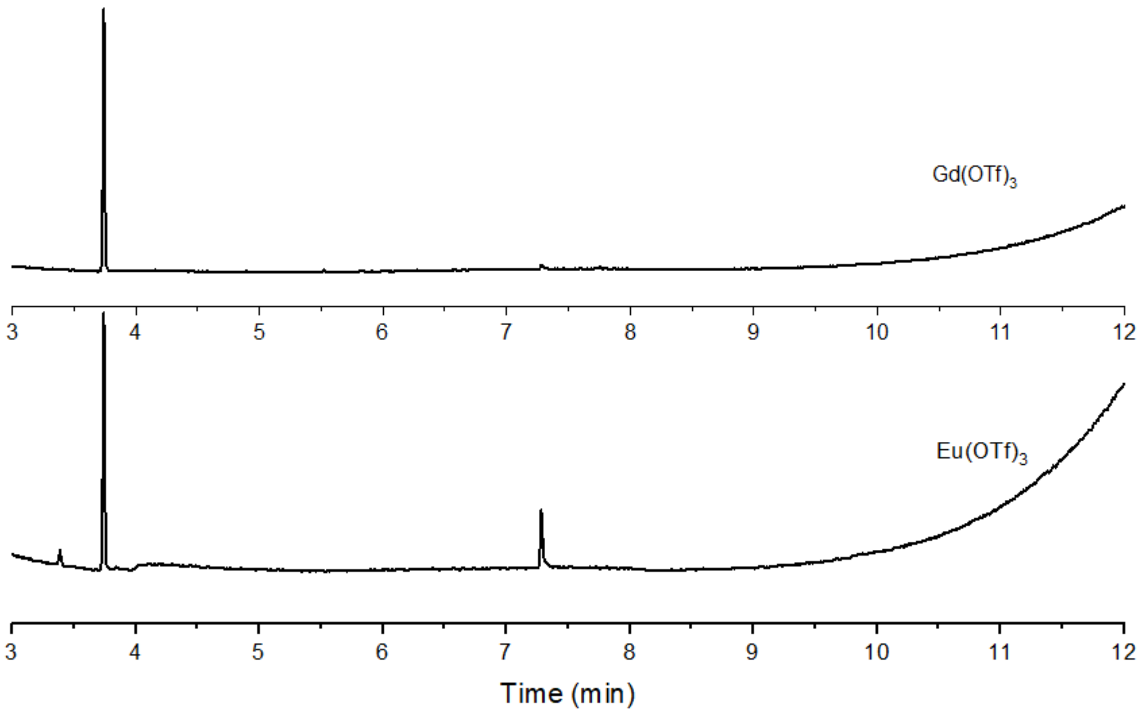

Figure S119. GC chromatograms of the reaction mixtures with **L9** with Zn in DMF.

9. ^{1}H NMR spectra

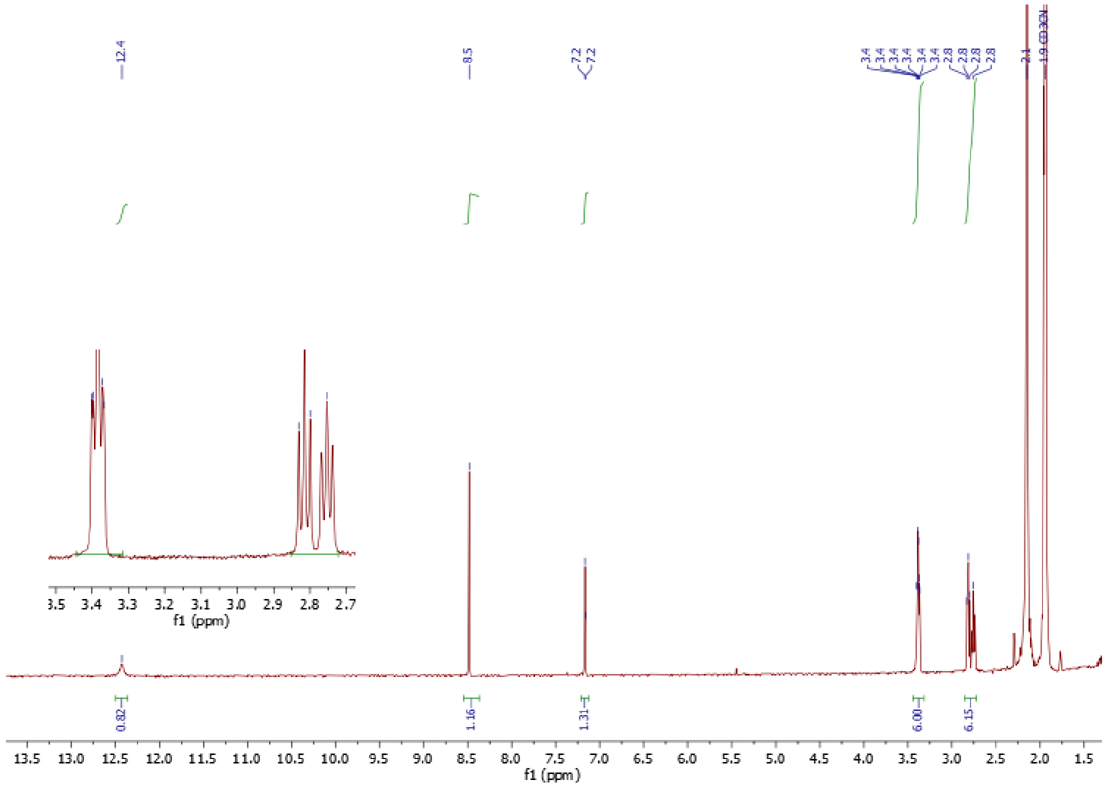

Figure S120. ^{1}H NMR spectrum of **L7** in CD_3CN.

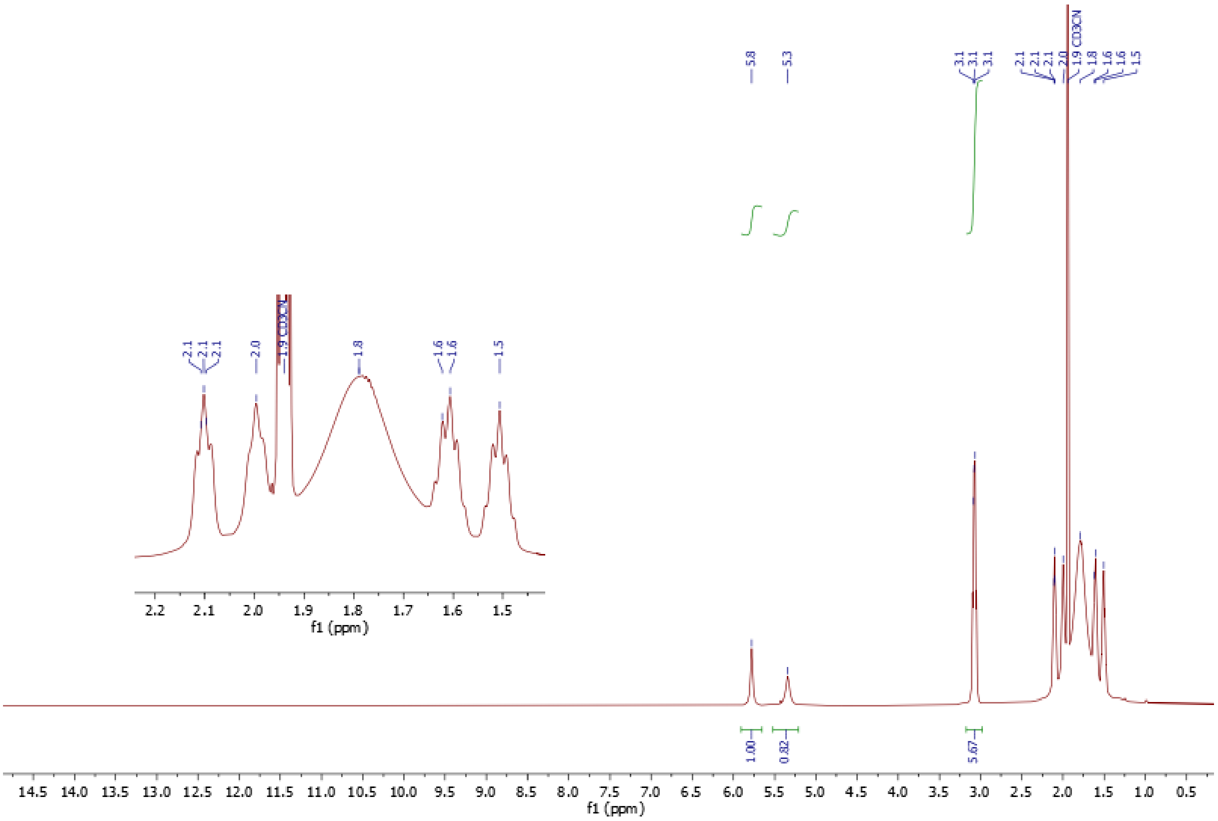

Figure S121. ^{1}H NMR spectrum of an equimolar mixture of **L7** and $Eu(OTf)_3$ in CD_3CN.

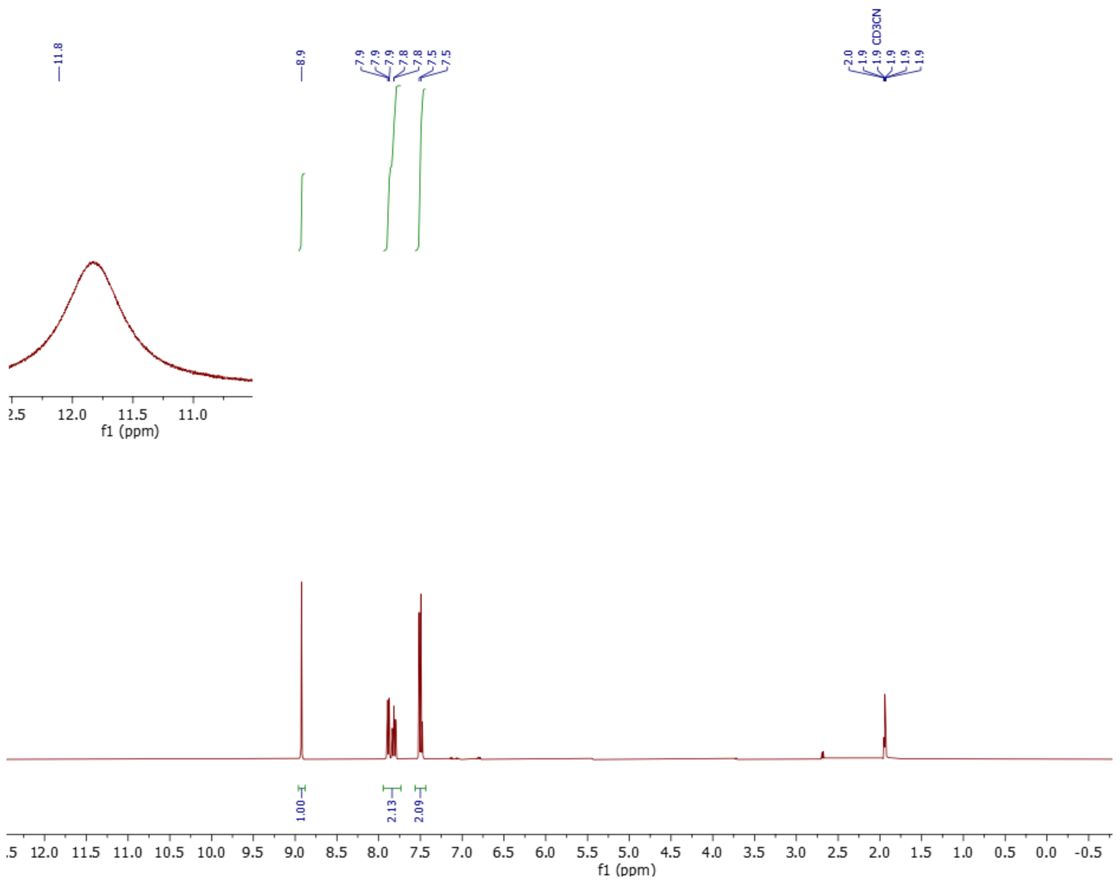

Figure S122. ^{1}H NMR spectrum of **L4** in CD_3CN.

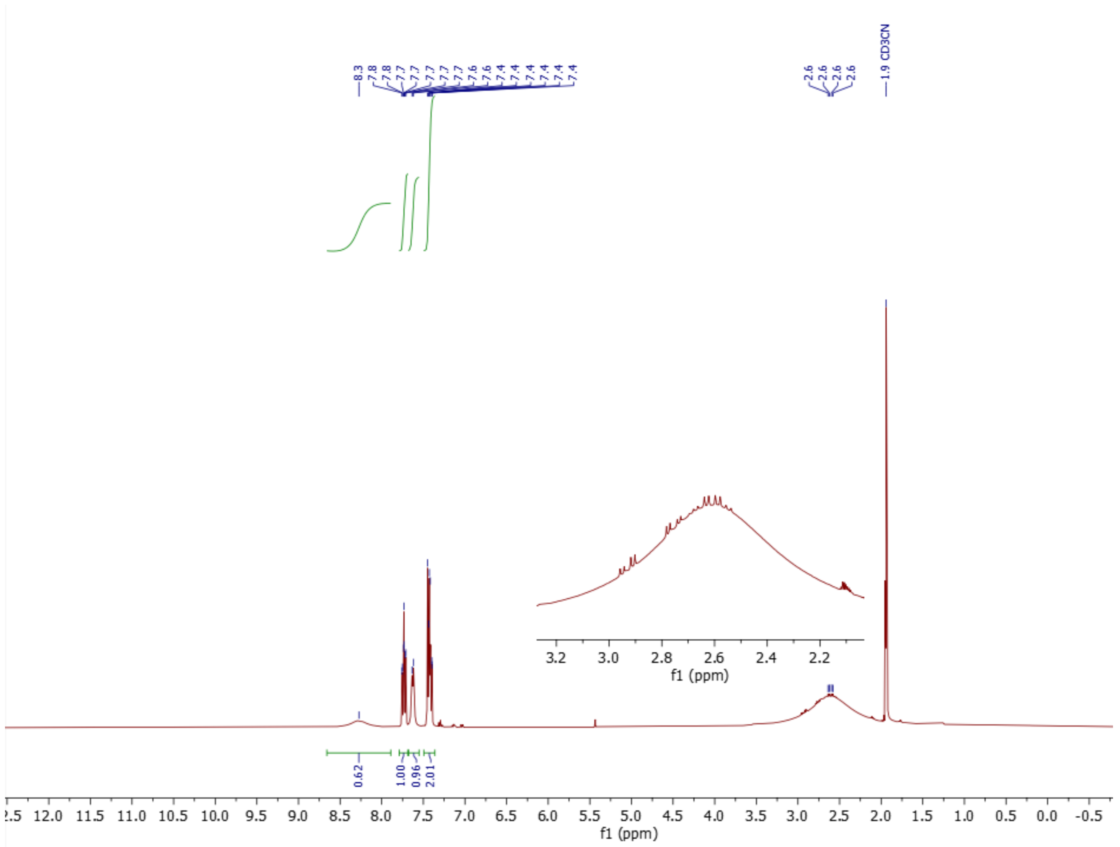

Figure S123. ^{1}H NMR spectrum of an equimolar mixture of **L4** and $Eu(OTf)_3$ in CD_3CN.

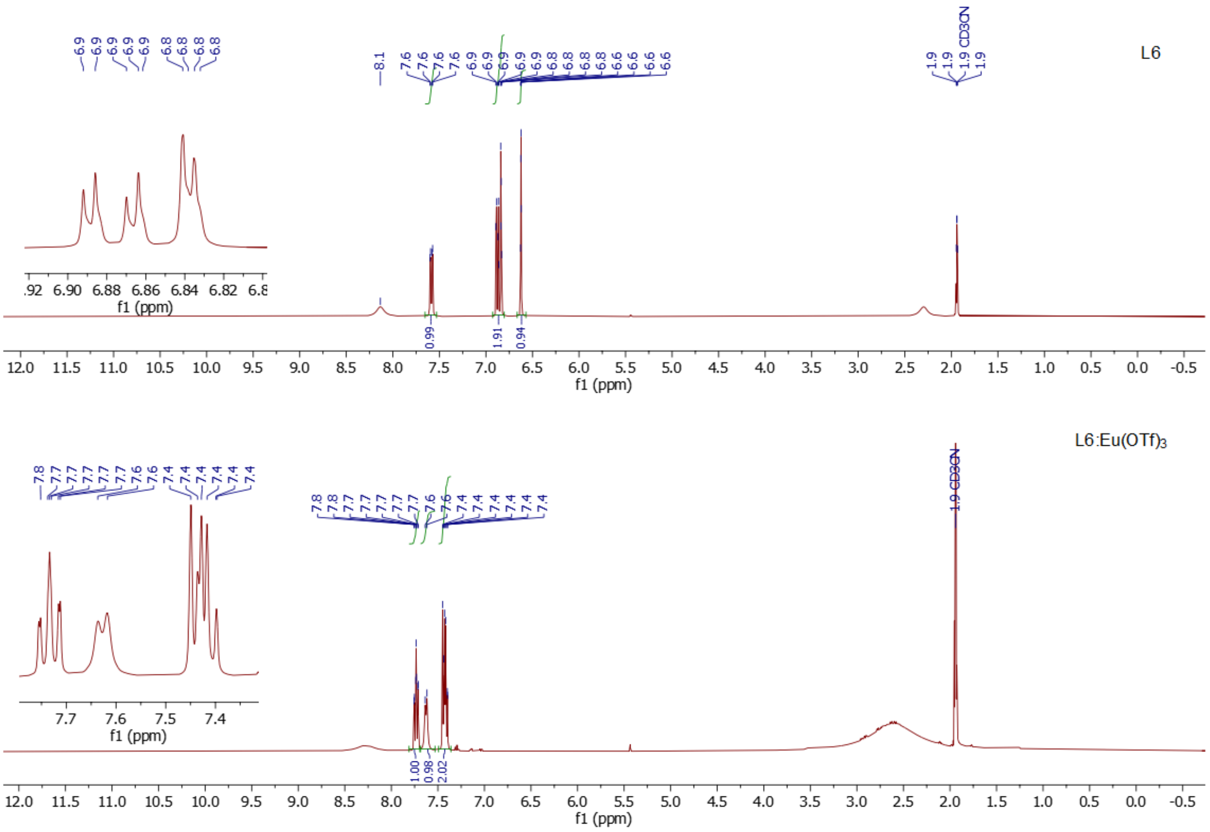

Figure S124. ^{1}H NMR spectrum of **L6** (top) and an equimolar mixture of **L6** and $Eu(OTf)_3$ in CD_3CN (bottom).

10. X-ray crystallography

Table S3 Crystal data and structure refinement for **L4:**Eu(III).

CCDC no.	2401537
Empirical formula	$C_{30}H_{23}EuO_{16}$
Formula weight	791.44
Temperature/K	100.00(10)
Crystal system	triclinic
Space group	P-1
a/Å	6.6817(2)
b/Å	14.3941(4)
c/Å	16.3388(2)
α/°	115.218(2)
β/°	94.084(2)
γ/°	94.266(2)
Volume/Å^3	1408.46(6)
Z	2
ρ_{calc}g/cm^3	1.866
μ/mm^{-1}	16.696
F(000)	788.0
Crystal size/mm^3	0.5 × 0.03 × 0.01
Radiation	Cu Kα (λ = 1.54184)
2Θ range for data collection/°	6.018 to 159.338
Index ranges	$-8 \le h \le 8$, $-17 \le k \le 18$, $-16 \le l \le 20$
Reflections collected	20158
Independent reflections	5956 [R_{int} = 0.0626, R_{sigma} = 0.0510]
Data/restraints/parameters	5956/4/441
Goodness-of-fit on F^2	1.028
Final R indexes [I>=2σ (I)]	R_1 = 0.0502, wR_2 = 0.1373
Final R indexes [all data]	R_1 = 0.0519, wR_2 = 0.1387
Largest diff. peak/hole / e Å^{-3}	2.54/–2.47

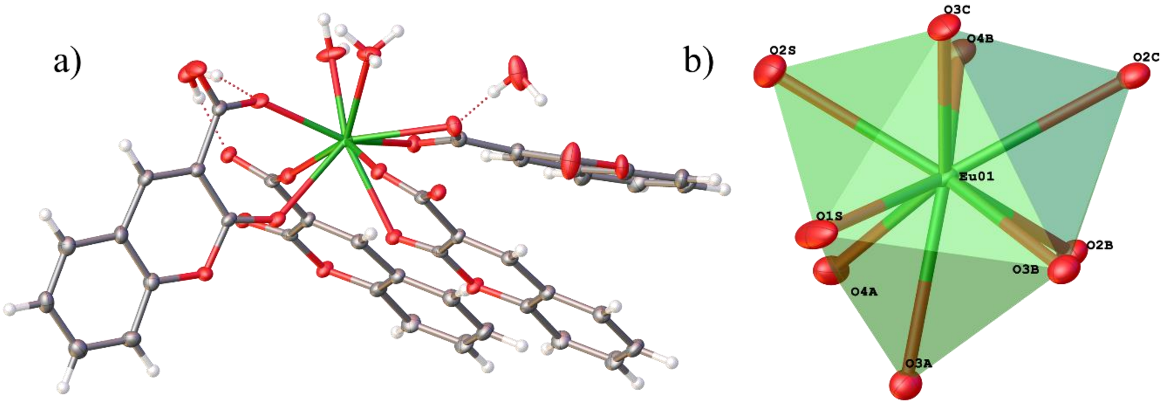

Figure 125. a) Representation of the solid state structure of **L4**:Eu(III). Ellipsoids plotted at a 50% probability level. b) Tricapped trigonal prismatic coordination environment of the Eu(III) centre.

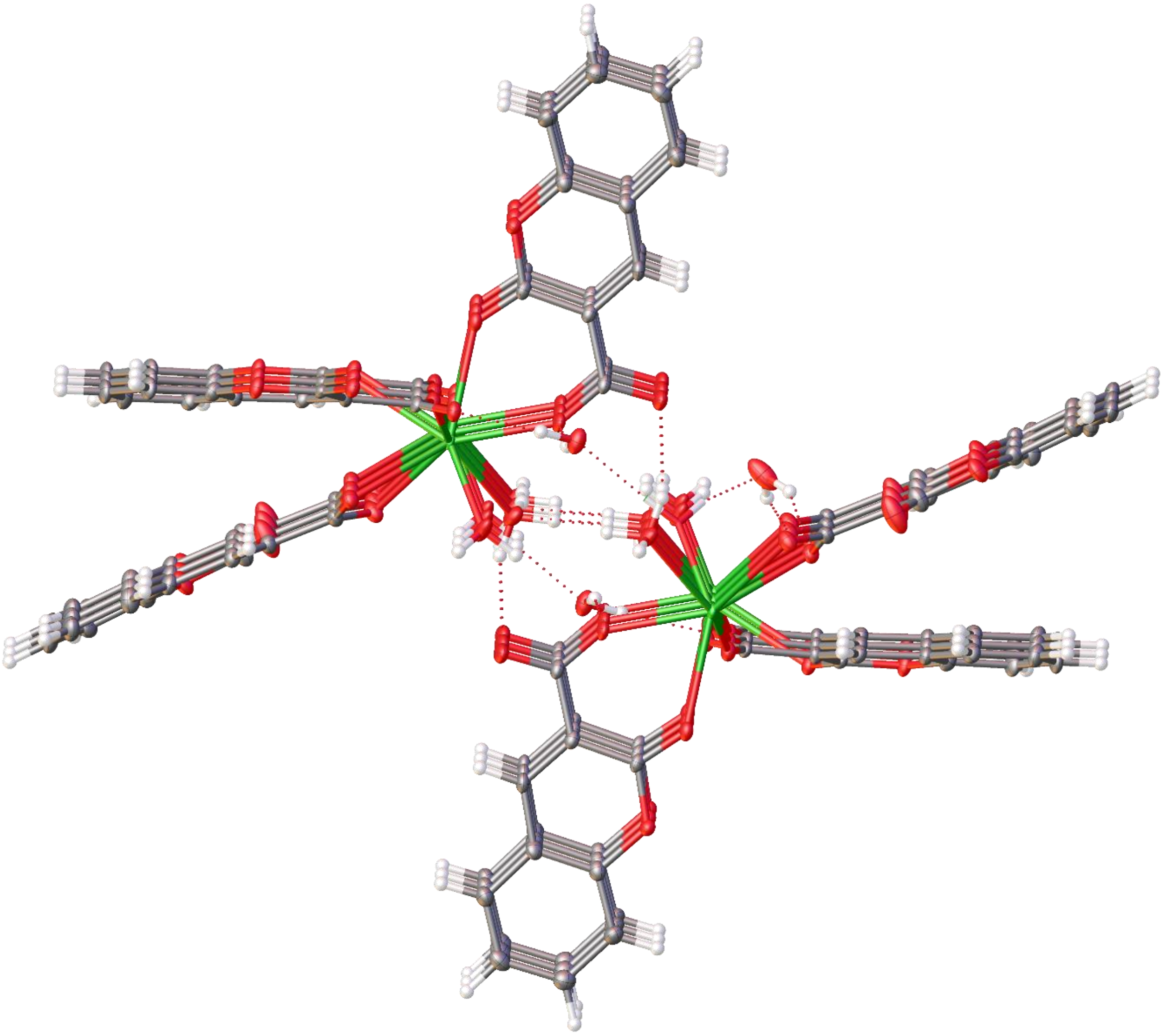

Figure 126. View along the double stranded 1D-coordination polymer highlighting the extensive hydrogen bonded network.

11. References

(1) Tomar, M.; Bhimpuria, R.; Kocsi, D.; Thapper, A.; Borbas, K. E. Photocatalytic Generation of Divalent Lanthanide Reducing Agents. *J. Am. Chem. Soc.* **2023**, *145* (41), 22555-22562.
(2) Suzuki, K.; Kobayashi, A.; Kaneko, S.; Takehira, K.; Yoshihara, T.; Ishida, H.; Shiina, Y.; Oishi, S.; Tobita, S. Reevaluation of absolute luminescence quantum yields of standard solutions using a spectrometer with an integrating sphere and a back-thinned CCD detector. *Phys. Chem. Chem. Phys.* **2009**, *11* (42), 9850-9860.
(3) Gundorff Nielsen, L.; Ravnsborg Hansen, A. K.; Stachelek, P.; Pal, R.; Just Sørensen, T. 1-Azathioxanthone Appended Lanthanide(III) DO3A Complexes That Luminesce Following Excitation at 405 nm. *Eur. J. Inorg. Chem.* **2023**, *26* (24), e202300245.
(4) Brouwer, A. M. Standards for photoluminescence quantum yield measurements in solution (IUPAC Technical Report). *Pure Appl. Chem.* **2011**, *83* (12), 2213-2228.
(5) Mutra, M. R.; Li, J.; Wang, J.-J. Light-mediated sulfonyl-iodination of ynamides and internal alkynes. *Chem. Commun.* **2023**, *59* (43), 6584-6587.
(6) Bokouende, S. S.; Ward, C. L.; Allen, M. J. Understanding the Coordination Chemistry and Structural and Photophysical Properties of Eu^{II}- and Sm^{II}-Containing Complexes of Hexamethylhexacyclen and Noncyclic Tetradentate Amines. *Inorg. Chem.* **2024**, *63*(37), 16991-17004.
(7) Basal, L. A.; Bailey, M. D.; Romero, J.; Ali, Meser M.; Kurenbekova, L.; Yustein, J.; Pautler, R. G.; Allen, M. J. Fluorinated Eu^{II} -based multimodal contrast agent for temperature- and redox-responsive magnetic resonance imaging. *Chem. Sci.* **2017**, *8*(12), 8345-8350.
(8) Dolomanov, O. V.; Bourhis, L. J.; Gildea, R. J.; Howard, J. A. K.; Puschmann, H. OLEX2: a complete structure solution, refinement and analysis program. *J. Appl. Cryst.* **2009**, *42*(2), 339-341.
(9) Sheldrick, G. SHELXT - Integrated space-group and crystal-structure determination. *Acta Cryst. A* **2015**, *71*(1), 3-8.
(10) Beeby, A.; Faulkner, S.; Williams, J. A. G. pH Dependence of the energy transfer mechanism in a phenanthridine-appended ytterbium complex. *J. Chem. Soc., Dalton Trans.* **2002**, (9), 1918-1922.
